# Quantifying cortex-wide traveling brain waves of complex patterns with a graph-based algorithm

**DOI:** 10.3389/fncom.2026.1844662

**Published:** 2026-08-24

**Authors:** Kuan-Ting Ho, Hirotaka Onoe, Tadashi Isa, Chih-Yang Chen

**Affiliations:** 1 Division of Physiology and Neurobiology, Department of Neuroscience, Graduate School of Medicine, Kyoto University, Kyoto, Japan; 2 Evolutionary Neuroscience Group, Institute for the Advanced Study of Human Biology (WPI-ASHBi), Kyoto University, Kyoto, Japan; 3 Human Brain Research Center, Graduate School of Medicine, Kyoto University, Kyoto, Japan; 4 Laboratory of Neuropsychopharmacology, Faculty of Pharmaceutical Science, Kobe Gakuin University, Kobe, Japan; 5 National Institute for Physiological Sciences, Okazaki, Japan; 6 Graduate School of Medicine, Tohoku University, Sendai, Japan

**Keywords:** electrocorticography, graph theory, marmoset, saccade, traveling wave

## Abstract

Traveling brain waves (TWs) are neural oscillations that propagate across the nervous tissue. Recently, wave detection algorithms have successfully linked TWs to various brain functions, including perception and movement. However, most existing approaches are not well-suited for large-scale recording systems that span multiple areas on highly curved surfaces. Here, we developed a framework that combines generalized phase analysis and a graph-based algorithm for detection of cortical traveling waves, enabling the decomposition of complex large-scale phase patterns into multiple simultaneously propagating TWs and the quantification of their properties in single-trial data. We applied the algorithm to hemispheric electrocorticogram recordings (82 and 122 channels) from two marmosets performing a visually-guided saccade task. We found that the 20–50 Hz activity in the occipital cortex during the first 100 ms following saccade offset forms a macroscopic TW. The wave originated in the primary visual cortex (V1) and propagated rostrally toward temporal and parietal areas. Moreover, the post-saccadic TWs originated from specific locations within V1 that were consistent with the retinotopic representations of the saccade targets. Wave parameters, including latency, duration, spatial coverage, and amplitude also depended on saccade direction. Combined with large-scale neural recordings, the graph-based framework introduced here provides a general approach for elucidating highly dynamic inter-areal communication and coordination mediated by TWs.

## 1 Introduction

Group activities of neurons cause measurable voltage alterations in the extracellular space, known as the local field potential (LFP) (Buzsáki et al., 2012). Rhythmic fluctuations in LFP form the basis of brain waves. Traditionally, synchronized brain waves within or between cortical areas have been considered to be the predominant mechanism of intra- and inter-areal communication (Fries, 2005; Uhlhaas et al., 2009). However, LFP phases can also be desynchronized across space, with increasing temporal delay along the cortical surface. This gives rise to traveling waves (TWs). Over the past two decades, TWs have become a major focus for neuroscientists, and have been associated with a variety of brain functions, including visual perception (Townsend et al., 2017; Davis et al., 2020), forelimb movement (Rubino et al., 2006; Takahashi et al., 2015), and working memory (Bhattacharya et al., 2022; Das et al., 2026).

To quantitatively characterize TWs, several methods have been developed based on the analytic signal of LFP. Wave direction at each sensor can be determined by, for example, calculating the phase velocity field (Townsend et al., 2015, 2017; Townsend and Gong, 2018; Liang et al., 2021, 2023), the phase gradient field (Muller et al., 2014, 2016; Davis et al., 2020), optical flow (Gutzen et al., 2024), or local circular-linear fitting (Das et al., 2023, 2026) of the Hilbert-based phase. These derived field of wave direction can then be used for TW detection or classification. However, these methods were primarily designed for two-dimensional planar data, such as calcium imaging, voltage-sensitive dye signals, microelectrode arrays, or local electrocorticography (ECoG) recordings. Extending them to non-planar datasets, including electroencephalography (EEG), magnetoencephalography (MEG), or large-scale ECoG arrays spanning highly curved surfaces, can be challenging.

Several properties of non-planar datasets complicate the application of existing TW detection methods. First, sensors are usually unevenly distributed across the cortical surface, making methods based on numerical gradient (Muller et al., 2014, 2016; Townsend et al., 2015, 2017; Townsend and Gong, 2018; Davis et al., 2020; Liang et al., 2021, 2023) more difficult to be applied directly. Although not explored in previous studies, a potential workaround is to perform surface fitting and analytically calculate the gradient, albeit at substantial computational cost. Second, because the recorded areas are typically much larger in these datasets, TW patterns may extend beyond simple planar, radial, or rotational forms. Methods that classify TWs using pre-defined models of these patterns may therefore struggle to capture more complex propagation dynamics (Muller et al., 2014, 2016; Zanos et al., 2015; Zhang et al., 2018; Davis et al., 2020; Bhattacharya et al., 2022). Exhaustive model fitting for all possible patterns is computationally impractical. Consequently, a sufficiently general and flexible framework is required to capture diverse TW patterns. Third, complex TW patterns in large-scale recordings may be composed of multiple simultaneously occurring waves. The ability to decompose a global phase pattern into individual TWs prior to classification is crucial for reducing analytical complexity and enhancing interpretability.

To address these challenges, we incorporated Hilbert-derived phase information and its direction of propagation into a graph-based representation (Supplementary Figure S1). Graph representations have been widely adopted in neuroscience to characterize anatomical or functional connectivity between brain regions (Bullmore and Sporns, 2009; Vecchio et al., 2017; Farahani et al., 2019), although physical proximity on the cortical surface is not always explicitly incorporated into the graph structure (but see Davis et al., 2021; Bolt et al., 2022; Budzinski et al., 2023). We focused on detecting TWs with distance-dependent phase delays propagating across spatial neighborhoods. This type of TW is consistent with the class of waves recently termed “first-order TWs” (Dugué and Chavane, 2025), which is adopted in most of the studies. By constraining graph connectivity according to physical proximity, we developed an algorithm that efficiently identifies large-scale phase-propagation patterns and decomposes them into individual TWs. We verified the algorithm by simulated data, demonstrating its robustness against moderate noise and its accuracy in parameter estimation.

Finally, we applied the algorithm to ECoG recordings collected from two marmosets performing a visually-guided saccade task. Following saccade offset, a large TW emerged from the primary visual cortex (V1) and propagated rostrally. The algorithm successfully isolated this TW from other activities and quantified its wave parameters.

## 2 Results

### 2.1 The graph-based algorithm

**Figure 1:**
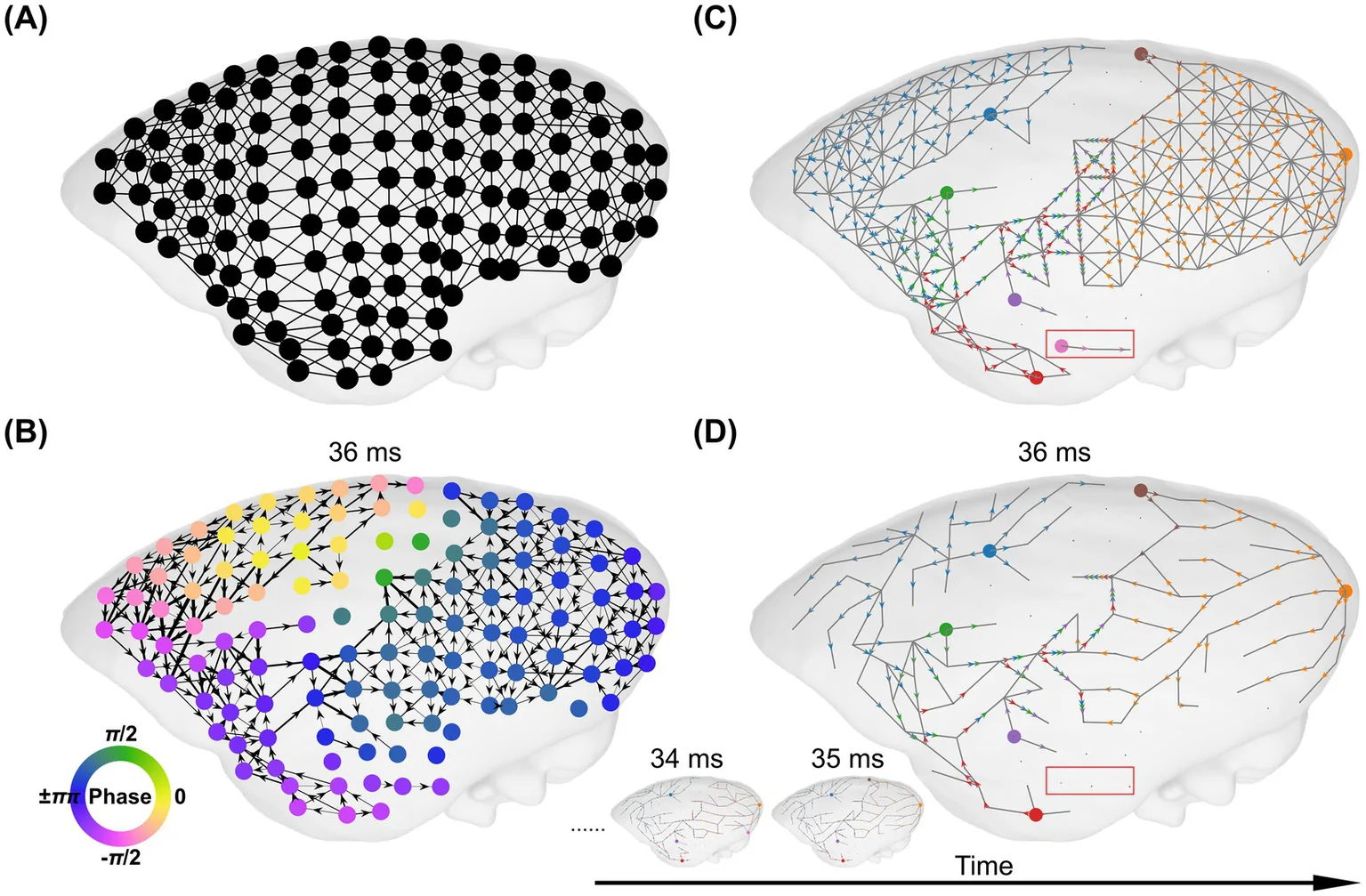
Step-by-step illustration of representative results of the graph-based algorithm. **(A)** Neighbor graph. Nodes are based on the electrode map of Marmoset L. **(B)** Phase gradient graph plotted on a phase map (from Figure 5, Marmoset L, 36 ms after saccade offset). Node color indicates phase. Arrows indicate the existence of wave flow. Edge width indicates phase gradient. All nodes present in **(A)** were retained in the phase gradient graph. Example data shown here is from 36 ms of Marmoset L in Figure 5, which contains multiple TWs, suited for demonstrating purpose. **(C)** Subgraphs of the phase gradient graph. Colored circles indicate the root nodes of the subgraphs. Edges reachable by the roots are marked with arrows in the same color. The red rectangle highlights a subgraph to be removed in **(D)** because of poor phase-distance correlation. **(D)** Maximum directed spanning tree (MDST) of the remaining subgraphs. Subgraph MDSTs of previous time points (34, 35 ms; sampling frequency of LFP data: 1,000 Hz) are also shown at the bottom left corner for comparison. The red rectangle indicates where the removed subgraph was.

To identify TWs in our large-scale recording system, we developed an algorithm based on the analytic signal (Smith, 2007) and graph-based representations. To constrain the search space, we modeled TW propagation as a gradual, monotonic phase change across neighboring electrodes over a period of time, potentially mediated by horizontal fibers with distance-dependent connection probabilities (Davis et al., 2021). Propagation mediated by distance-independent, long-range association fibers is beyond the scope of the current work. Taking this constraint into account, a graph containing all possible TW propagation paths can be constructed for a given electrode map (Figure 1A). Specifically, we constructed a weighted “neighbor graph,” in which nodes represent electrodes and edges connect immediately neighboring nodes (see Materials and method 5.9.1 for definition), weighted by the geodesic distance between the two nodes on the cortical surface mesh.

To determine existence of wave flow along each edge of the neighbor graph, the instantaneous phase (IP) and period at each node were calculated from the corresponding analytic signals (Figure 1B, node color). Node pairs not sharing a similar period are excluded. In addition, spatial changes in IP were required to be sufficiently gradual—meaning the phase gradient could not be too high—to ensure sufficient spatial sampling. For a given temporal frequency, the maximally allowed phase gradient was determined by assuming a minimum propagation velocity of 500 mm/s, substantially lower than the empirical velocity (1,000–10,000 mm/s) of macroscopic TWs (Hughes, 1995; Muller et al., 2018). By contrast, setting a minimum phase gradient (i.e., a maximum velocity) was unnecessary, as noise usually disrupts linear phase changes in extremely low phase gradient case, allowing such case to be rejected during later processing steps. These two criteria substantially reduced the number of edges to be searched.

The next step is to assess the temporal similarity between the two signals of each remaining pair, around the timepoint of interest. For each pair, fragments of the two IP time series were extracted. Each fragment was centered at the timepoint of interest and spanned approximately one quarter of a period, over which wave dynamics were assumed to be stationary. The central timepoint of either fragment was then shifted to minimize their mean absolute phase difference (MAPD). The minimum MAPD serves as a non-parametric and computationally efficient indicator of similarity between temporally shifted signals. If both minimum MAPDs were sufficiently small (i.e., below a given threshold) and the two optimal time shifts (which minimize the MAPDs) were symmetric around zero, we considered this pair to exhibit wave flow. Wave direction was then determined by the signs of the optimal time shifts (negative to positive, see Supplementary Figure S2).

A directed graph was subsequently constructed from the neighbor graph, containing only edges with confirmed wave flow (Figure 1B, arrows). The edge weights were replaced by the phase gradient between node pairs. Higher phase gradients indicate that the edges are closely aligned with the propagating direction of waves, facilitating estimation of wave directions in subsequent steps. Nodes containing only outgoing edges were designated as “root nodes” (Figure 1C, colored circles), representing tentative wave sources. To determine the spatial extent of each TW, graph searches were initiated from the root nodes to identify all the reachable nodes and edges, thereby forming induced subgraphs derived from the parent phase gradient graph (Figure 1C). Each subgraph represented the spatial extent of a TW at a given moment. While the above procedure detects source waves, such as radial-out or spiral-out waves, sink waves can be detected by reversing either the temporal sequence of the LFP data or the edge directions in the phase gradient graph.

Edges of the phase gradient subgraphs were retained as long as they pointed to approximately the wave direction. Consequently, not all edges equally reflected the actual wave direction. To approximate the true propagation path, edges with low phase gradients need to be eliminated without disconnecting any node in the subgraph. This is equivalent to finding the maximum directed spanning tree (MDST) of the subgraph (Figure 1D). For each MDST, the linear-linear correlation between cumulative geodesic distance and cumulative phase difference measured from the root node was used as a criterion to eliminate statistical insignificant subgraphs (Figures 1C,D, red box). Unlike some existing methods for detection of radial waves that estimate linearity based on the shortest distance and phase difference between each electrode and the source, our approach follows the actual propagation trajectory of the wave, making it effective for curved patterns such as spiral waves.

Finally, to determine the temporal extent of a TW, subgraphs identified at the current timepoint were compared with those at previous timepoints (Figure 1D, bottom left). Subgraphs from consecutive timepoints were concatenated if they share the same or neighboring root nodes, forming the spatiotemporal representation of a TW. Allowing root to be succeeded by a neighboring nodes provided additional flexibility in cases where a wave source was located extremely close to the center of two electrodes or drifted over time. At each timepoint, wave properties such as amplitude and frequency were calculated for each node, and velocity was calculated for each edge. Pseudocode and a complete mathematical description of the algorithm and selection of parameters are provided in Materials and method 5.9 and Supplementary materials 1.1, 1.2.

### 2.2 Benchmark with simulated waves

**Figure 2:**
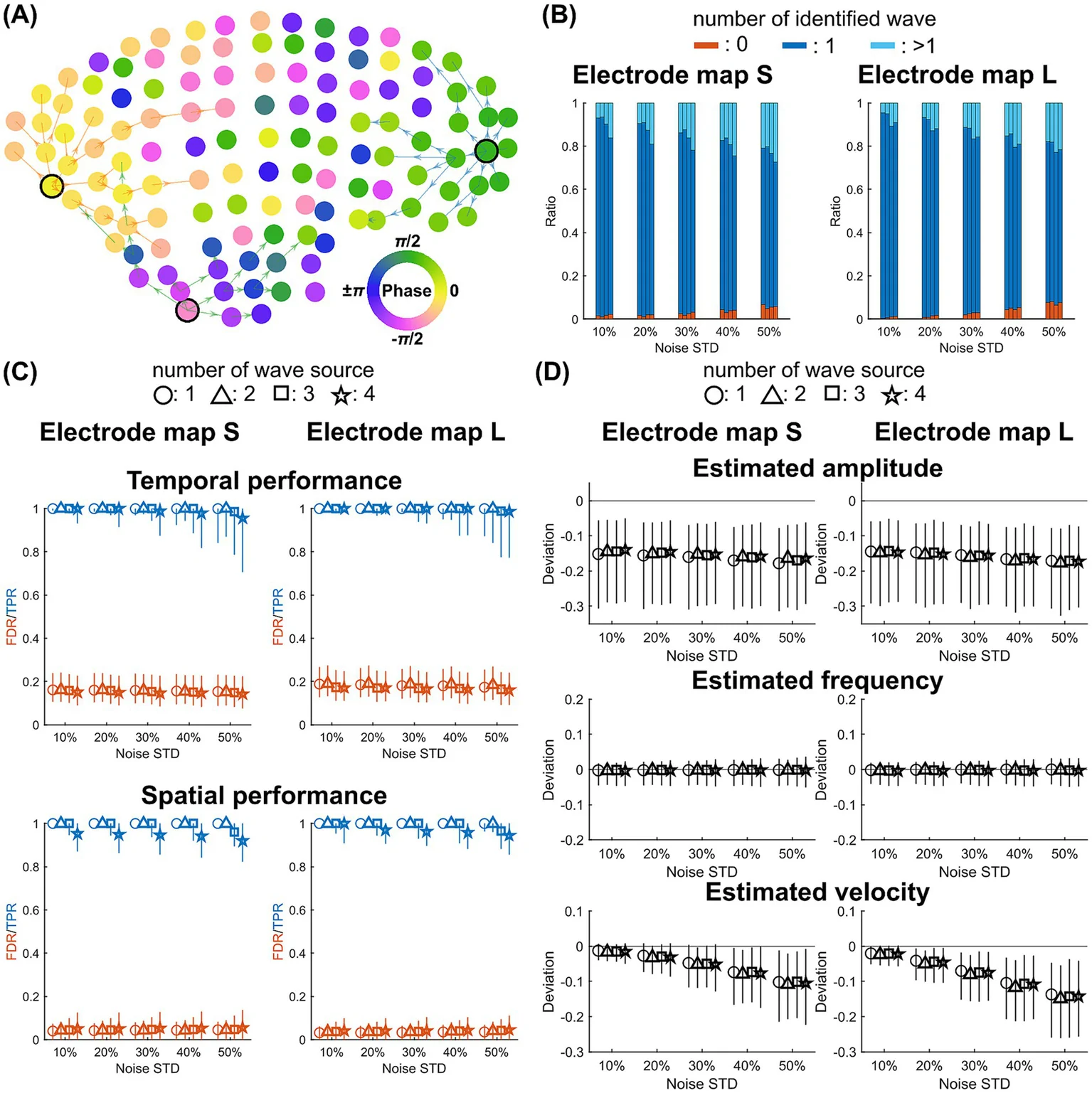
Simulated traveling waves. **(A)** Snapshot of an example simulation with 3 wave sources on Electrode map L. Node colors indicate instantaneous phase. Black circles indicate wave sources. Arrows indicate simulated wave flow. **(B)** In each bar group, from left to right are 1 to 4 wave source conditions. In conditions with multiple waves, each wave was analyzed independently. The ratio of miss (0 identified wave, orange), exact match (1 identified wave, blue), and fragmented wave (>1 identified wave, cyan) are shown for each condition. For each combination of wave source number and noise level, 10,000 waves were generated. Noise STDs are relative to unit Gaussian noise. **(C)** Top row: Temporal true positive rate (TPR, blue lines) and false discovery rate (FDR, orange lines). For fragmented waves, the temporal scope was calculated as the union of all fragments. Bottom row: Spatial TPR (blue lines) and FDR (orange lines). The spatial scope of a wave was defined as nodes existing in more than 50% of the wave’s lifespan. **(D)** Accuracy of wave parameter estimation. Top row: Deviation ratio of the estimated amplitude (temporal median of root amplitude). Middle row: Deviation ratio of the estimated frequency (spatiotemporal median of node frequency). Bottom row: Deviation ratio of the estimated velocity (spatiotemporal median of edge velocity). In **(C,D)**, symbols indicate the median and the two ends of the vertical line indicate the 1st and 3rd quartiles of the 10,000 simulated waves in each condition.

To verify the reliability of our graph-based algorithm, we simulated TWs with known parameters on the electrode maps of the two marmosets (Supplementary Figure S3, Supplementary Table S1). Up to four simultaneous waves were generated. To ensure the simulated TWs were sufficiently complex and realistic, the spatial extent of each wave was determined using a probabilistic depth-first search algorithm (pDFS, see Materials and method 5.10). The pDFS allows TWs to propagate anisotropically in different directions, and to change propagation direction over time. TWs were simulated using parameters comparable to those observed in our ECoG recordings (see Materials and method 5.10), and were contaminated with Gaussian white noise at varying levels to test the robustness of the algorithm (Figure 2A, Supplementary Videos S1, S2). For each combination of wave source number and noise level, 10,000 simulated waves were generated.

Figure 2B shows the number of identified waves for each simulated wave (source amplitude = 1) under various conditions (number of wave source: 1–4, noise STD: 10–50% of unit Gaussian noise). At moderate noise levels (≤ 20%), most simulated waves (~80%) were correctly identified as single wave. At higher noise levels, some waves (~20%) were temporally interrupted and identified as multiple fragments. Nevertheless, the ratio of undetected waves remained low (<10%). We examined the temporal (Figure 2C, top row) and spatial (Figure 2C, bottom row) true positive rates (TPRs), and false discovery rates (FDRs). The temporal TPRs were close to 100% in most conditions. The temporal FDR of approximately 20% was mainly attributable to temporal smoothing introduced by the wide-band analytic signal and rather than to the algorithm itself. Temporal smoothing near the onset and offset of waves led to overestimation of wave duration, especially in the absence of intervening activity between consecutive waves. This edge effect was more pronounced for waves with short duration relative to their oscillatory periods. Spatial TPRs remained high (>90%) and the spatial FDRs remained low (<10%) even under the most challenging condition.

Next, we evaluated the estimated amplitude, frequency, and propagation velocity by calculating source amplitude, node frequency, and edge velocity, respectively (see Materials and method 5.10). The median deviation of the estimated amplitude (Figure 2D, top row) remained stable at approximately −15% across all conditions. The high variation within each condition resulted primarily from attenuation by the band-pass filter near the cutoff frequencies (Supplementary Figure S4A) and, to a lesser extent, from the temporal overestimation described above (Supplementary Figure S4B). Accordingly, when quantitatively comparing amplitude across frequencies, correction based on the frequency-response curve of the filter is recommended. Frequency estimation (Figure 2D, middle row) remained highly accurate (median deviation < 1%) across all conditions. The underestimation of wave velocity (Figure 2D, bottom row) increased linearly with noise level but remained relatively low (<10%) even at 30% noise, which represent a comparatively high noise level for most electrophysiological setups.

To further test whether our algorithm captures specific patterns such as spirals, we additionally simulated spiral waves centered around the temporoparietal junction to mimic patterns reported in human ECoG studies (Muller et al., 2016; Das et al., 2026). Our algorithm successfully detected the simulated spiral waves, and both direction and spirality were quantified from their graph representations (Supplementary Video S3).

Overall, the benchmark results demonstrate that the graph-based algorithm provides high temporal and spatial sensitivity, low false detection rates, and accurate estimation of wave parameters at moderate noise levels.

### 2.3 The macroscopic traveling wave from the occipital cortex after saccades

**Figure 3:**
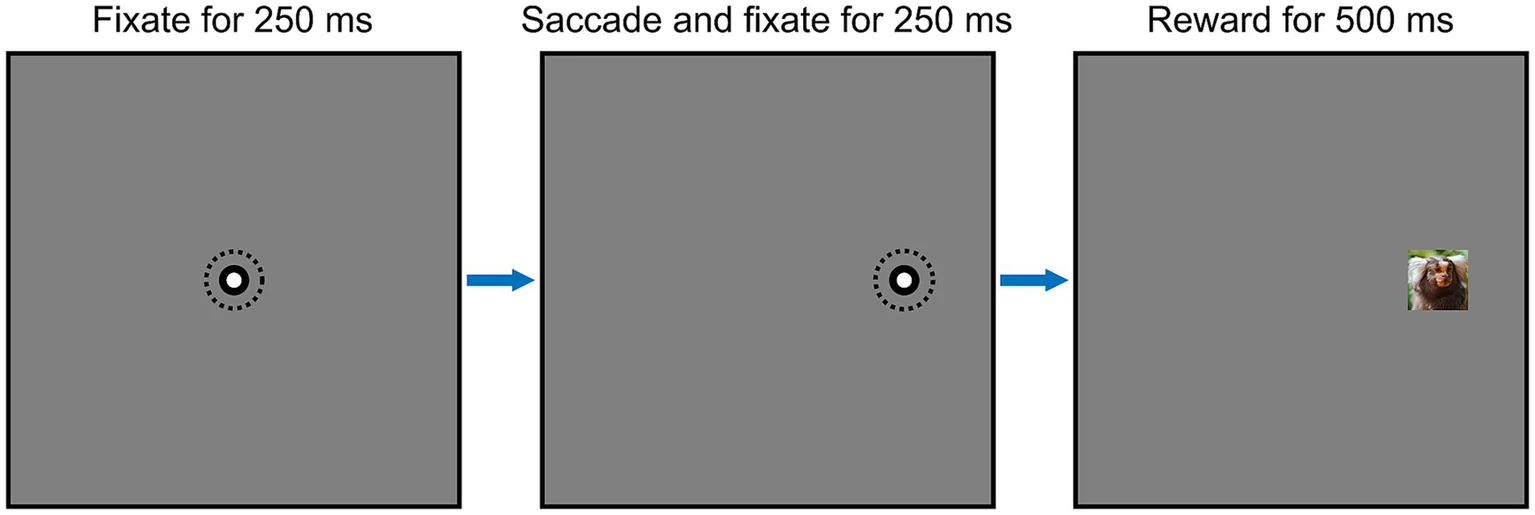
The visually-guided saccade task. Marmosets fixated on an annulus stimulus presented at the monitor center (dotted circle indicates eye window, not visible to the marmosets) for 250 ms. Then the stimulus jumped to a peripheral location (eccentricity: 4°, 5°, 6°, 7°, 8°, 9°; direction: 0°, 45°, 90°, 135°, 180°, 225°, 270°, 315°), and the marmosets made a saccade to and fixate on it for 250 ms. If successful, the stimulus would be replaced by a picture of marmoset face, and a liquid reward would be delivered. Fixation break or response timeout (1,000 ms) would terminate the trial without reward.

**Figure 4:**
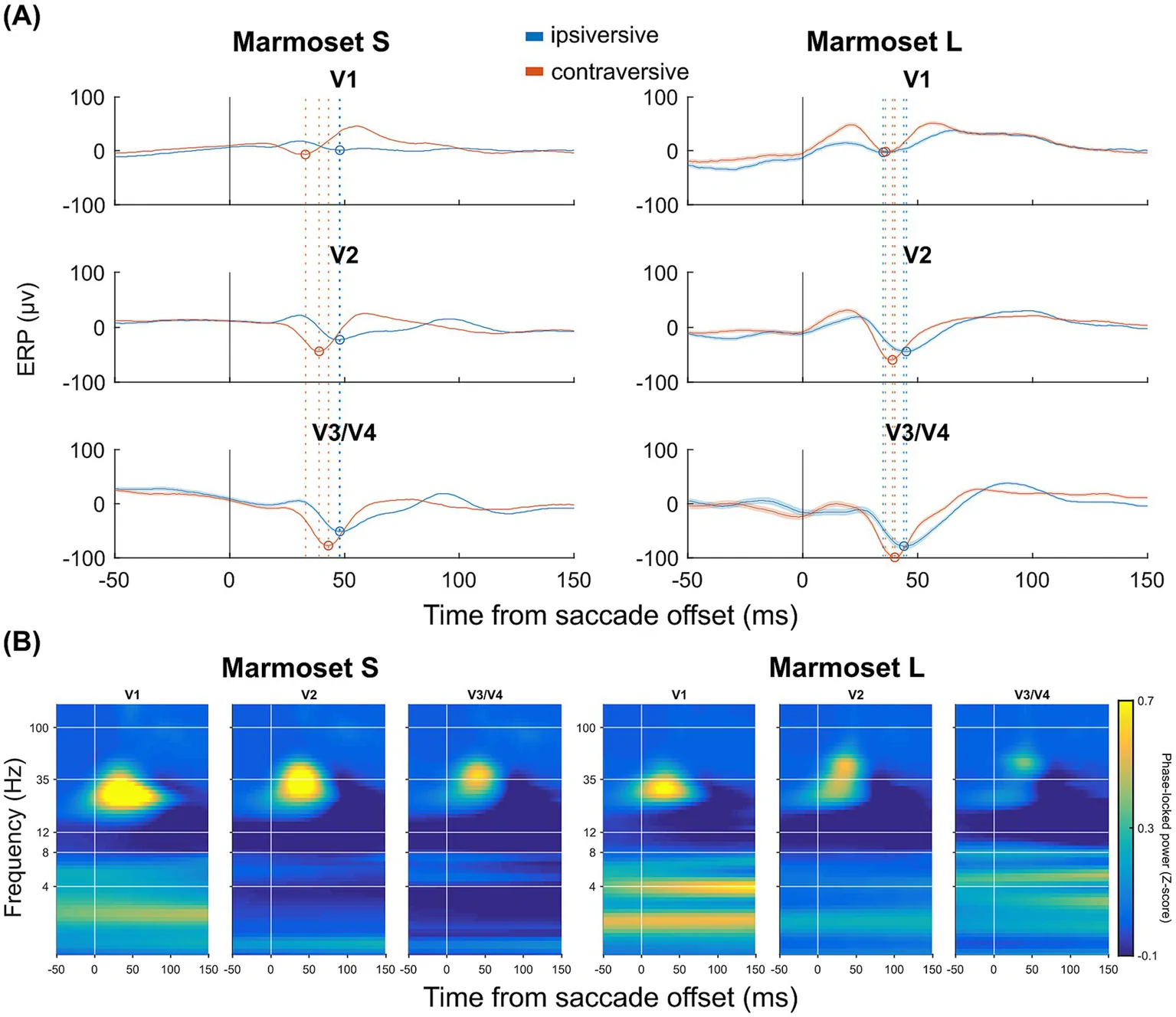
Brain activities around saccade offset. **(A)** The event-related potential (LFP) around the saccade offset. LFP data from trials having the same saccade direction (different eccentricity pooled) were averaged electrode-wise. Data from electrodes on the area of interest (Supplementary Table S1, Supplementary Figure S3, V3/V4 include A19DI, V3, V4, V4T) were averaged and shown for saccades ipsiversive (blue; Marmoset S, *n* = 1,355; Marmoset L, *n* = 1,168) or contraversive (red; Marmoset S, *n* = 1,281; Marmoset L, *n* = 1,046) to the implanted side (Marmoset S: Right, Marmoset L: Left). The shaded area indicates the 99% confidence interval (i.e., mean ± 2.58 × standard error of the mean). Circles and dotted lines indicate ERP troughs. **(B)** Trial-averaged phase-locked power around offset of contraversive saccades. Data was *Z*-scored using the mean and standard deviation of the entire recording session (i.e., one task block). Borders of conventional frequency bands are plotted (Delta: 1–4 Hz, Theta: 4–8 Hz, Alpha: 8–12 Hz, Beta: 12–35 Hz, Gamma: 35–100 Hz, High gamma: 100–160 Hz). Data criteria and sample size are the same as **(A)**.

Two marmosets were implanted with hemispheric ECoG arrays (Supplementary Figure S3, Marmoset S: 82 valid channels, Marmoset L: 122 valid channels) and performed a visually-guided saccade task (Figure 3). Within the first 100 ms following saccade offset, a triphasic response was observed in the event-related potential (ERP) recorded from the occipital cortex (Figure 4A). ERPs associated with saccades ipsiversive to the ECoG array (Marmoset S: 0°, Marmoset L: 180°) were generally weaker than those associated with contraversive saccades (Marmoset S: 180°, Marmoset L: 0°), reflecting higher inter-trial variance in latency (Supplementary Table S2). In addition, ERPs associate with ipsiversive saccades were delayed relative to those associated with contraversive saccades. For contraversive saccades, the averaged trough latencies increased systemically along the caudal-to-rostral axis from V1 (median values; Marmoset S, V1: 33 ms, V2: 39 ms, V3/V4: 43 ms; Marmoset L, V1: 37 ms, V2: 39 ms, V3/V4: 40 ms). We also attempted to align the data to saccade onset, but did not consistently reduce variability in ERP feature timings (Supplementary Table S3). Therefore, we decided to align the data on saccade offset to respect the post-saccadic nature of this activity.

To characterize the frequency components of the ERP, we performed the time-frequency analysis (TFA) and calculated both normalized power and inter-trial phase clustering index (ITPC). ITPC ranges from 0 to 1 and quantifies the consistency of signal phases at specific times relative to an event across trials. The product of normalized power (Supplementary Figure S5B) and ITPC (Supplementary Figure S5C) therefore reflects activity whose power and phase are both time-locked to the event (i.e., the phase-locked power, Figure 4B). As shown in Figure 4B, phase-locked activity in the high-beta to low-gamma range (20–50 Hz) during the first 100 ms after saccade offset exhibited a latency gradient along the caudal-rostral axis. Taking into account the temporal smoothing effect by trial averaging, the peak-to-peak interval of the ERP in V1 (marmosets S: 41 ms; marmoset L: 45 ms) was consistent with the centroid of this activity at approximately 30–35 Hz (period ≈ 30 ms). This activity appeared to “smear upward” in the frequency domain as moving rostrally from V1. However, based on the ERP waveform, the high-frequency power was likely an artifact arising from the non-sinusoidal waveform, in which the troughs deviated more strongly from baseline than the peaks. Based on the latency gradients observed in both the ERP and TFA results, we hypothesized that this 20–50 Hz phase-locked activity represents a traveling wave.

**Figure 5:**
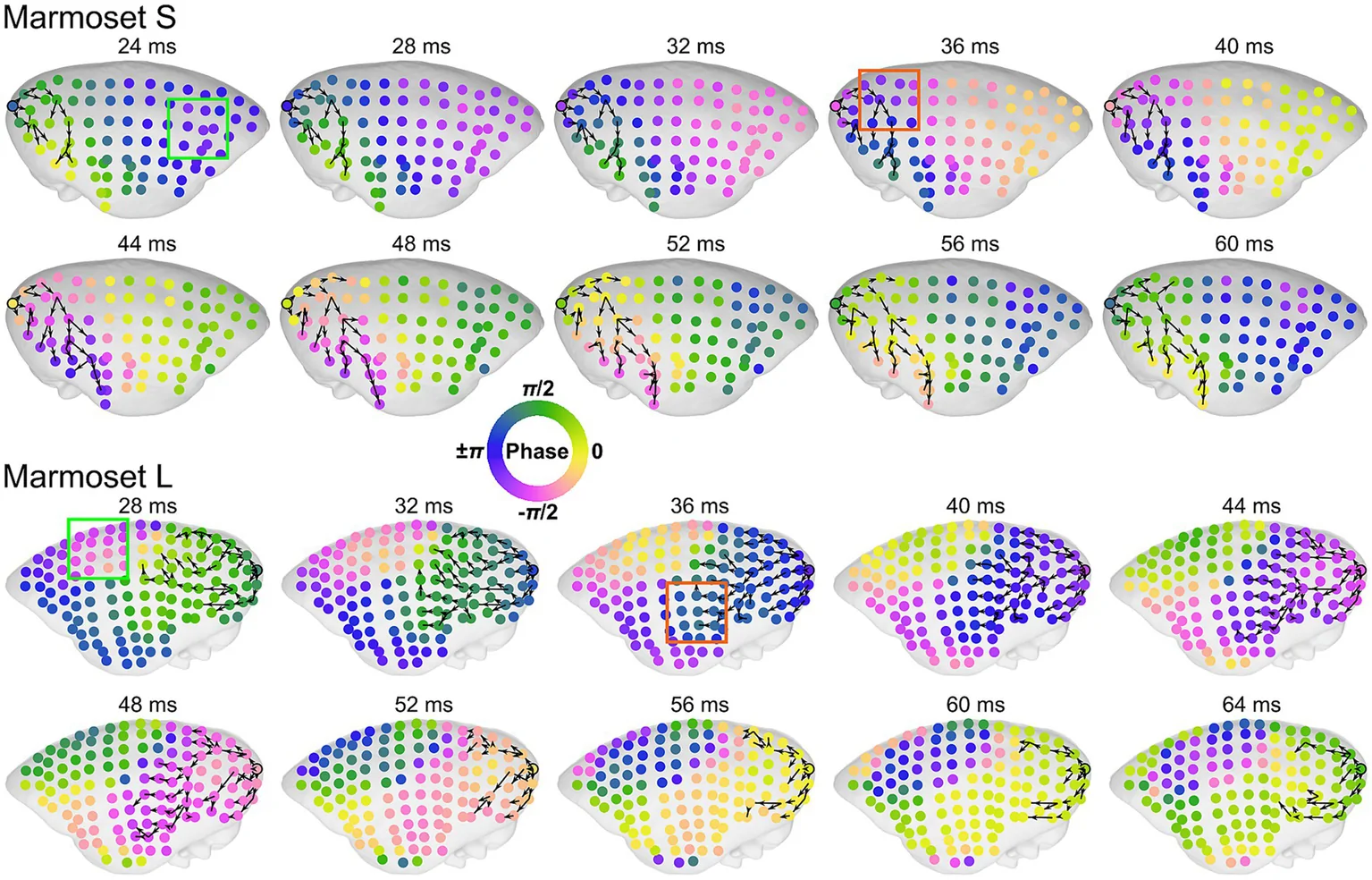
Examples of the post-saccadic TW. Single trial data of each marmoset are shown (above: Marmoset S, below: Marmoset L). Colors indicate the phase of the analytic signal filtered at 20–50 Hz. Black arrows mark the post-saccadic TW from V1 identified by the graph-based algorithm. Green box indicate the source of other TWs. Orange boxes indicate ambiguous areas, which are still well separated by the algorithm.

To test this, we adopted the concept of “generalized phase” proposed by Davis et al. (2020). Briefly, we first applied the Hilbert transform to band-pass filtered (20–50 Hz) single trial LFP data to derive the instantaneous phase. We then removed and interpolated segments where the phase was decreasing (due to high-frequency intrusions) to enforce a monotonically increasing linearized phase time series. Using a wide-band filter captures dominant dynamics while minimizing phase distortion (Davis et al., 2020). The lower cutoff frequency of 20 Hz was chosen to exclude activity at approximately 15 Hz, which exhibited high power (Supplementary Figure S5A) but was not saccade-related (Supplementary Figure S5B). The upper cutoff frequency of 50 Hz excluded non-phase-locked high-gamma activity emerging at approximately 30 ms (Supplementary Figures S5B,C). The upper cutoff additionally minimized potential contamination from local spike-related activities (Zanos et al., 2011), although such contamination was unlikely given the epidural placement of the electrode arrays. When instantaneous phases were plotted across consecutive time points on the electrode map (node color in Figure 5, Supplementary Videos S4, S5), a clear spatiotemporal gradient was observed, suggesting the presence of a TW. This macroscopic TW originated in the occipital cortex, and propagated rostrally toward temporal and parietal areas. Phase maps of the trial-averaged analytic signal exhibited similar gradients (Supplementary Videos S6, S7), indicating that the TW pattern was consistent across trials.

We next applied the graph-based algorithm to data from both marmosets. Arrows in Figure 5 indicate the directions of identified TW from V1. Notably, the algorithm successfully separated this TW from other simultaneously occurring activities (e.g., green boxes at 24 ms for Marmoset S and at 28 ms for Marmoset L in Figure 5). Such concurrent activities varied across trials, making single-trial analysis challenging for many existing methods. Moreover, in boundary regions, the phases of distinct activities occasionally coincided (e.g., orange boxes at 36 ms in Figure 5, comparing the V1-originated wave with waves originating from other cortical regions). Even under such conditions, the algorithm correctly identified the boundaries between activities because information from the preceding and subsequent timepoints was incorporated when determining wave flows. This would not be possible using methods based solely on instantaneous phase maps.

### 2.4 Properties of the post-saccadic TWs

**Figure 6:**
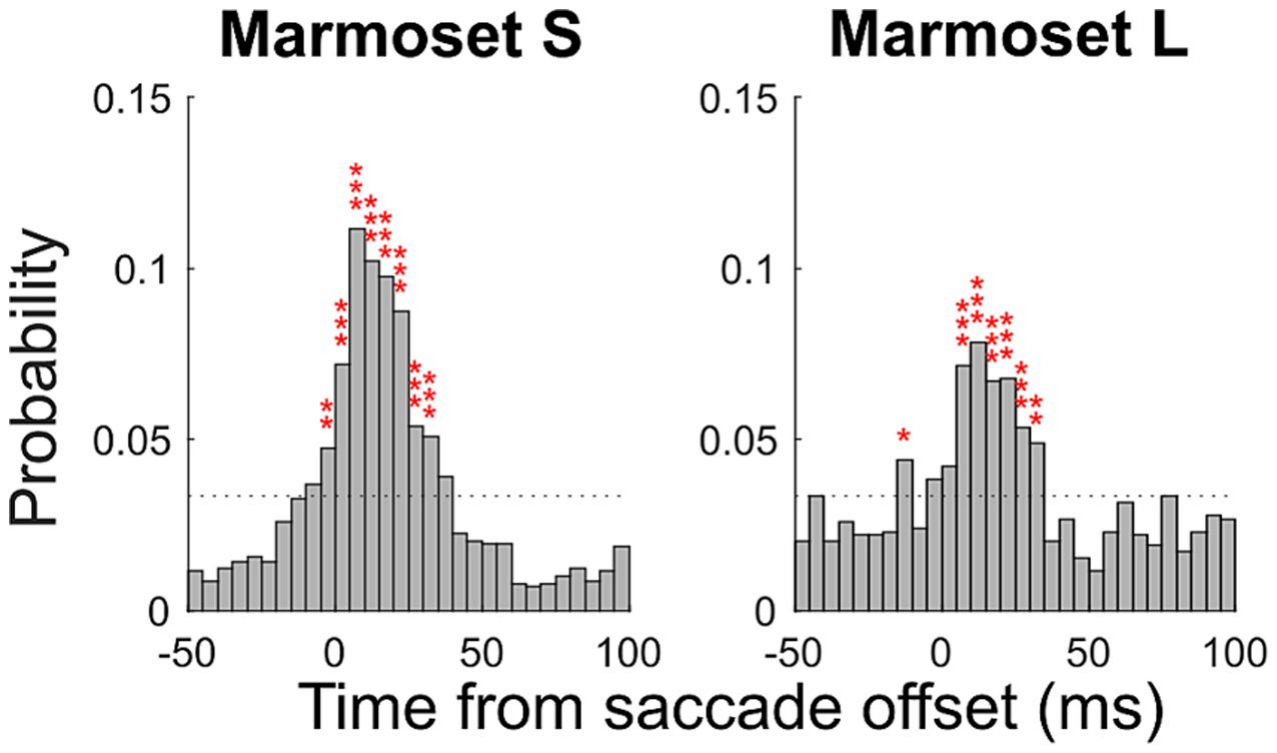
Latency of the largest TW around contraversive saccade offset. Probability histograms of wave latency for contraversive saccades are shown. Only the largest wave over the visual area (see Supplementary Table S1) originating from V1 surrounding saccade offset (−50 to 100 ms) in each trial was included (sample size; Marmoset S: 1281; Marmoset L: 1045). Data were partitioned into 30 bins, each 5 ms wide. Red asterisks indicate the one-tailed *p*-value under the binomial distribution against a chance level of 1/30 (*p < 0.05, **p < 0.01, ***p < 0.001).

To specifically isolate the observed macroscopic TWs, waves originating from area V1 during −50 to 100 ms relative to saccade offset were identified for each trial. The waves exhibiting the largest spatiotemporal extent within the visual areas (see Supplementary Table S1 for definition) were extracted and binned according to their onset timing (Figure 6 and Supplementary Figure S6). In both marmosets, the largest waves occurred more frequently during −10 to 40 ms relative to saccade offset, consistent with the timing of the ERP (Figure 4A) and the phase-locked power (Figure 4B). This tendency was more pronounced for saccades containing a contraversive component (Supplementary Figure S6, central panels). To evaluate the possibility that the TWs were triggered by target onset rather than by saccades, we plotted saccade reaction time (SRT) against TW latency aligned to saccade offset (Supplementary Figure S7, surrounding panels). If the TWs were time-locked to target onset, a negative linear correlation between the two latencies would be expected. However, no such relationship was observed. Furthermore, splitting the data based on median SRT showed no consistent difference in wave latency (Supplementary Figure S7, central panel), further supporting saccade-offset alignment. To be more specific, only the largest TWs occurring between −10 and 40 ms relative to saccade offset were included in the following analyses.

**Figure 7:**
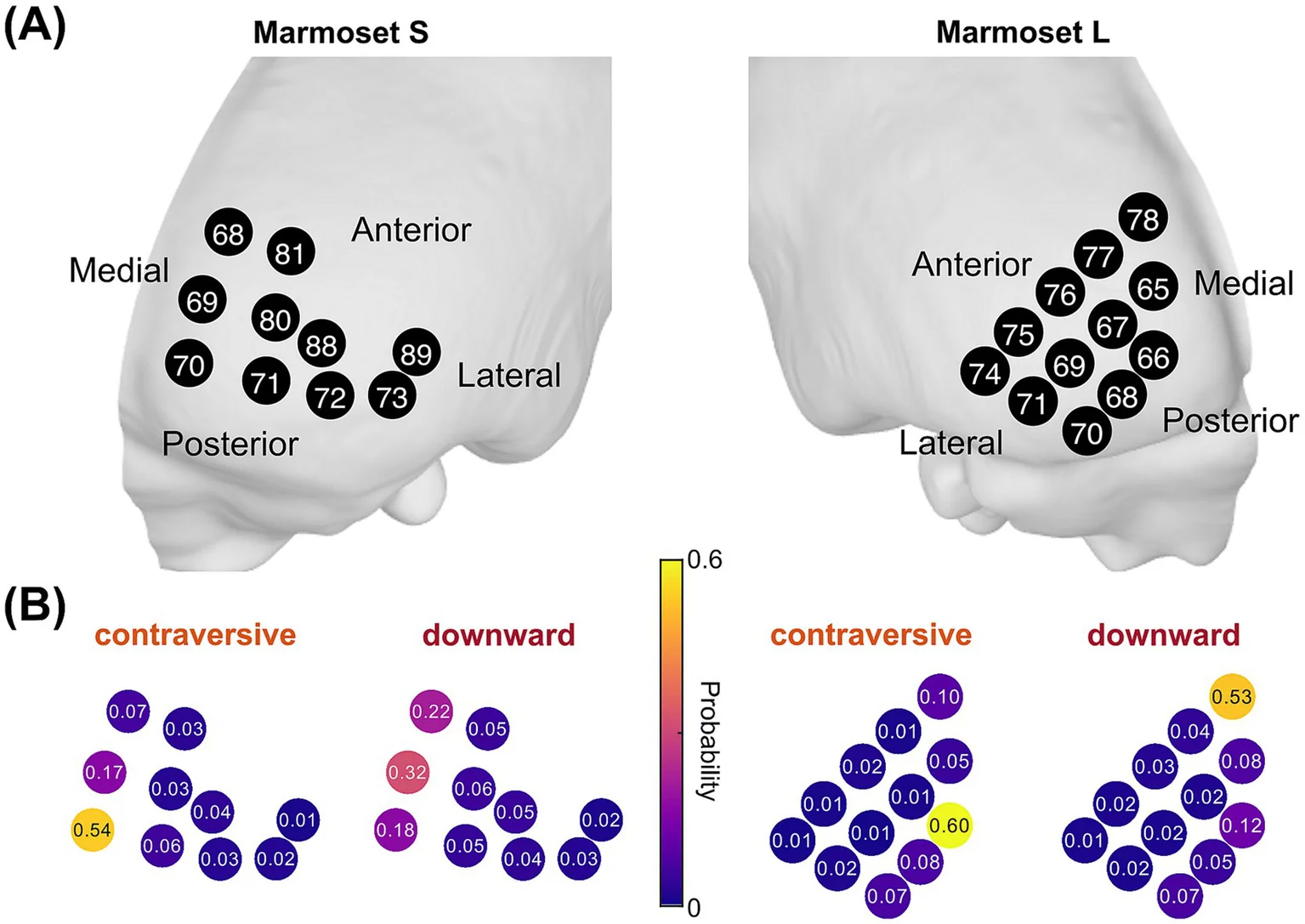
Distribution of wave source on V1. **(A)** Locations of the V1 electrodes on the brains. **(B)** Probability for a TW to originate from each V1 electrode after contraversive and downward saccades (left and right panels of each marmoset, respectively). Only trials with at least one identified TW occurring during −10 to 40 ms surrounding saccade offset were included (sample size; Marmoset S, contra: 1250, down: 999; Marmoset L, contra: 1009, down: 1052). Note that the probabilities do not sum up to 1, because the source of the largest wave can be outside of V1.

To investigate potential modulation of wave properties by saccade directions, we first examined the spatial distribution of wave sources (Figure 7 and Supplementary Figure S8). Following contraversive saccades (Marmoset S: 180°, Marmoset L: 0°), TWs most frequently originated from the most caudomedial electrodes (Marmoset S: 70, Marmoset L: 66), whereas this tendency was less apparent for saccades lacking a contraversive component. After downward saccades (270°), most TWs originated from dorsomedial electrodes (Marmoset S: 69, Marmoset L: 78), a pattern unique for saccades with downward component. Notably, these findings were consistent with the retinotopic representation of the saccade target on V1 (Solomon and Rosa, 2014). Such correspondence was not observed for upward saccades (90°), likely due to a lack of electrode coverage over ventral V1, where the upper visual field is represented. Because marmosets commonly exhibit undershooting saccades, a control analysis was performed using only overshooting saccades to ensure the source differences were not a trivial result of varied retinal locations of the target upon fixation. Qualitatively similar results were still obtained (Supplementary Figure S9), arguing against this explanation. Differences in source distribution between the two marmosets likely reflected slight variation in electrode placement: V1 electrodes in Marmoset S were positioned more anteriorly than Marmoset L. Accordingly, the actual wave source following contraversive saccades in Marmoset S was likely caudal to electrode 70.

**Figure 8:**
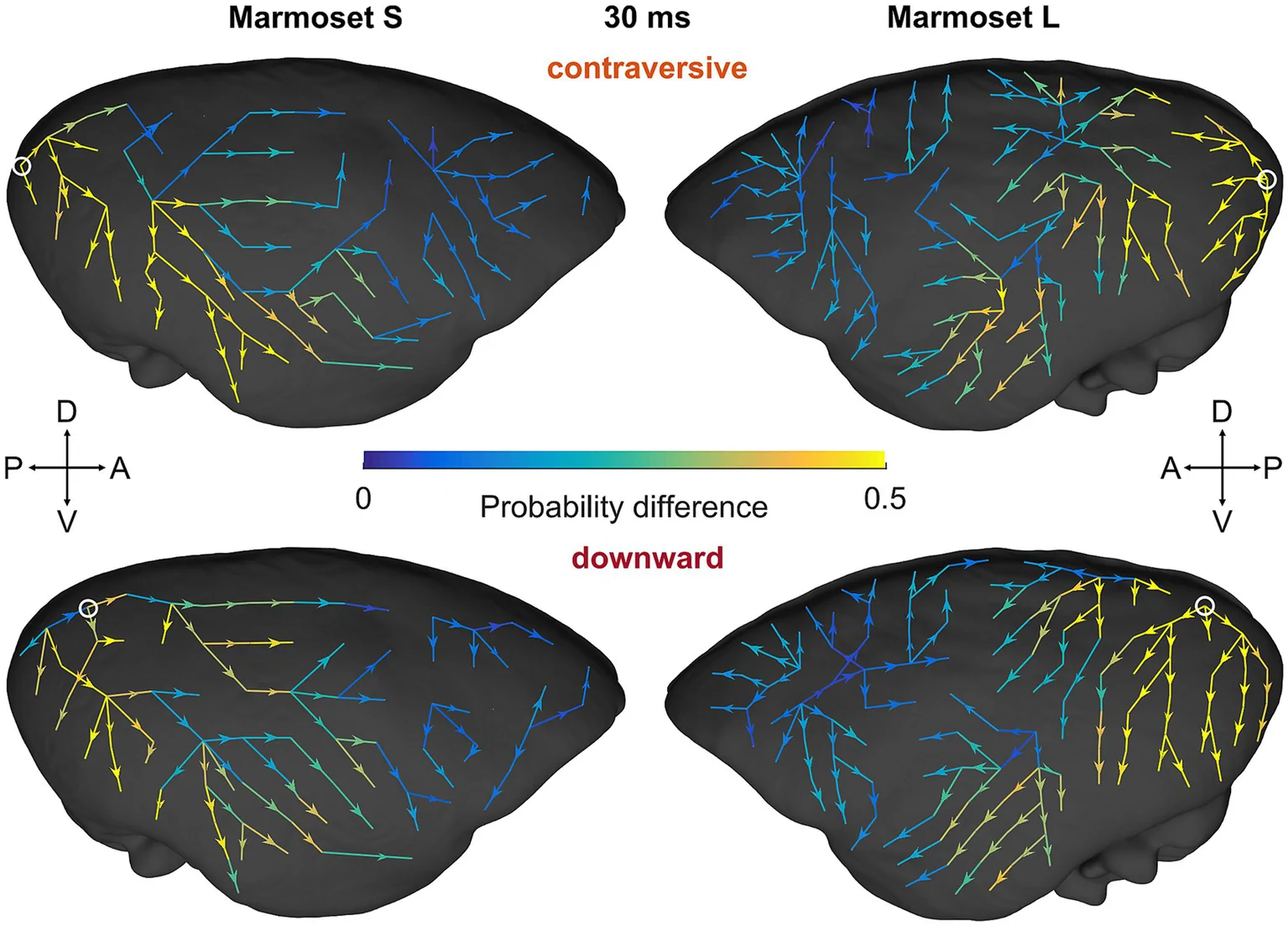
Wave pattern identified by the asymmetry of edge probability. The color indicates the difference in wave probability, which is higher if the wave flows in one direction more frequently than the opposite direction along the same edge. Pearson’s chi-squared test was performed on the probabilities of waves flowing in the two opposite directions, and only edges with *p*-value < 0.001 were kept. For simplicity, only edges of the MDST of the probability difference graph are shown. Data at 30 ms after saccade offset are shown for contraversive and downward saccades (see also Supplementary Videos S4A,B for the evolution of wave pattern of all the eight saccade directions). The white circles indicate the most frequent sources in Figure 7.

To further characterize differences in global wave patterns across saccade directions, we next examined whether a wave flow was present between each neighboring electrode pair (i.e., each edge in the phase gradient graph) at time points surrounding saccade offset for every trial. For each neighboring electrode pair, we then calculated the probability that a wave flowed in one direction over the opposite direction, and constructed a directed graph weighted by this probability difference. Finally, the probability differences were presented on the MDST graph to visualize the dominant wave flows over time (Figure 8, Supplementary Videos S8, S9). A major advantage of this approach over inter-trial phase averaging is that the graph-based algorithm distinguishes TW-occurring regions from non-TW regions, and thereby avoids contamination from unrelated activities during averaging. Following contraversive saccades (Marmoset S: 180°, Marmoset L: 0°), TWs originated from caudal V1, propagated rostrally, and subsequently extended ventrally toward temporal regions. Following downward saccades (270°), TWs originated from dorsal V1 and bifurcated into ventral and dorsal streams, targeting temporal and parietal areas, respectively. The wave sources identified by this pattern analysis (Figure 8, white circles) were consistent with the source probability analysis (Figure 7) and corresponded to the retinotopic representation of the targets in V1.

**Figure 9:**
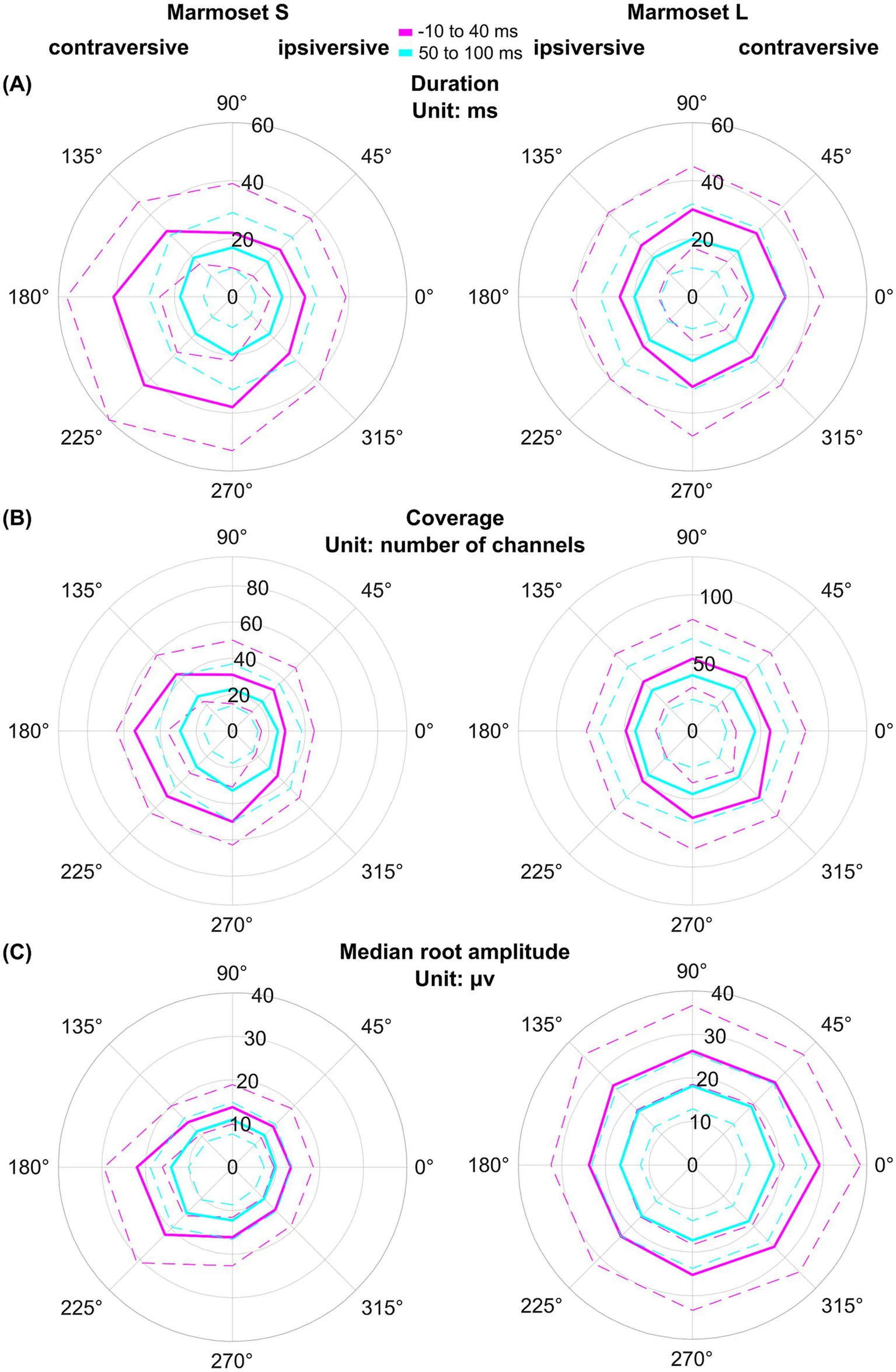
Parameters of the post-saccadic TW. The polar plots show the distribution of wave parameters for each of the eight saccade directions, for the largest TW occurring during −10 to 40 ms (magenta) or 50 to 100 ms (cyan) surrounding saccade offset. The solid lines indicate the median of the parameter, and the dashed lines indicate the 1^st^ and 3^rd^ quartile. **(A)** Wave duration. **(B)** Temporal maximum of covered electrodes. **(C)** Temporal median of root node amplitude.

Finally, we examined the modulation of wave properties by saccade direction, including temporal duration, spatial coverage, amplitude, frequency, and propagation velocity of the largest TW occurring during the −10 to 40 ms interval. These properties were additionally compared with those of the largest V1 TWs (same definition) observed in a later interval from 50 to 100 ms following saccade offset. In brief, TWs occurring during the −10 to 40 ms interval following contraversive and downward saccades exhibited longer duration (Figure 9A and Supplementary Figure S10A), broader spatial coverage (Figure 9B and Supplementary Figure S10B), and greater amplitude (Figure 9C and Supplementary Figure S10C) than those following ipsiversive and upward saccades. TWs observed during the 50 to 100 ms interval were reduced in all three properties (Supplementary Table S4), and showed weaker modulation by saccade direction. Modulation was less pronounced for frequency (Supplementary Figures S10D, S11A) and propagation velocity (Supplementary Figures S10E, S11B). The median duration of the −10 to 40 ms TWs was around 30 ms, shorter than the duration observed in the ERP (Figure 4A) and phase-locked power (Figure 4B), which persisted for approximately 50 ms. This discrepancy likely resulted from partial overlap of TWs with variable latencies during inter-trial averaging, again emphasizing the importance of single trial analysis for accurate quantification of wave properties. The median spatial coverage of the TWs exceeded the number of electrodes covering the visual areas (Supplementary Table S1; Marmoset S: 32, Marmoset L: 45), confirming that the TWs propagated beyond visual cortex into temporal and parietal regions. Median TW frequency remained stable at approximately 30 Hz across all saccade directions and both intervals. At this frequency, amplitude attenuation introduced by the filter was minimal (Supplementary Figure S4A), allowing accurate amplitude estimation without correction. Median propagation velocities was approximately 1,000–2,500 mm/s, consistent with previously reported values for macroscopic TWs (1,000–10,000 mm/s), which were suggested to propagate via myelinated white matter fibers (Muller et al., 2018). These findings further support the validity of the assumed minimum propagation velocity of 500 mm/s used to derive the upper limit of the phase gradient.

In summary, the graph-based algorithm revealed that the observed TWs (1) occurred predominantly during the interval from −10 to 40 ms following saccade offset, (2) were more strongly time-locked and prominent following contraversive and downward saccades, (3) originated from distinct locations within V1 and propagated along different trajectories, depending on saccade direction, and (4) were macroscopic in scale, spanning most visual areas and extending into temporal and parietal regions.

## 3 Discussion

In this study, we developed a graph-based algorithm that effectively identifies multiple, simultaneously occurring TWs recorded by non-uniformly distributed electrode arrays on curved brain surfaces. Using simulated data, we demonstrated that the algorithm provides high sensitivity and specificity, and accurately estimates wave parameters. Applying the algorithm to marmoset ECoG recordings, we revealed that the post-saccadic high-beta to low-gamma activity represents a macroscopic TW. This wave originates within V1 and propagates rostrally, spanning occipital, temporal, and parietal regions. Most TWs occurred during the interval from −10 to 40 ms following saccade offset, and originated from specific locations in V1, depending on saccade direction. In addition, TWs after contraversive and downward saccades exhibited greater duration, spatial coverage, and amplitude.

### 3.1 The graph-based algorithm

#### 3.1.1 Comparison with existing methods

The graph-based algorithm developed in this study has several advantages over existing methods for TW detection and estimation of wave parameters. First, it is applicable to a broader range of recording systems with arbitrary sensor placement. Recent studies identified wave sources by locating the electrode with maximal divergence on the phase map (Muller et al., 2016; Davis et al., 2020), an approach that provides unbiased results only for uniformly distributed electrode arrays. High curvature of the recorded surface further complicates the estimation of wave parameters which depends on the grid-like placement of sensors. By contrast, our method treats arbitrarily positioned electrodes as a collection of neighboring electrode pairs, enabling more flexible estimation of wave sources and higher accuracy of other parameters, at the level of single electrode pair.

Second, our algorithm makes fewer assumptions regarding wave patterns. Conventionally, different TW patterns are identified using distinct models and criteria. For example, circular-linear correlation between phase and distance from a reference line or a putative source has been used to detect planar (Zhang et al., 2018) or radial waves (Davis et al., 2020), respectively. Other approaches fit data to predefined wave equations (Zanos et al., 2015; Zhang et al., 2018) or compare empirical phase maps with model-predicted results (Bhattacharya et al., 2022). However, TWs—especially those recorded by large-scale systems—may not conform to any predefined template. As demonstrated by our data, macroscopic TWs are highly dynamic spatiotemporal structures; their directions and patterns can evolve as they propagate. With minimal prior assumptions, our algorithm can detect TWs of diverse propagation patterns while separating wave detection from wave classification, which can be performed independently in subsequent steps.

Third, methods that fit instantaneous phase maps to smooth models (Townsend et al., 2015, 2017; Townsend and Gong, 2018; Liang et al., 2021, 2023; Das et al., 2023, 2026) may introduce artifactual smoothing that confounds TW detection. By contrast, our approach preserves information from wide-band waveforms by utilizing the generalized phase (Davis et al., 2020). Matching segments of the generalized phase ensures waveform similarity during propagation in addition to linear phase change (Supplementary Figure S2), substantially reducing the risk of false positives.

Fourth, a key feature of the proposed framework is its modularity (similar to the idea of Gutzen et al., 2024). In the present implementation, local propagation between neighboring nodes was determined using the mean absolute phase difference (MAPD) after temporal alignment. We selected MAPD because it provides a computationally efficient estimate of phase similarity between time-lagged signals as well as their delay, which was advantageous given the large number of pairwise comparisons required in the present dataset. However, the framework itself does not depend on a specific measure of pairwise propagation. Alternative edge-defining metrics, including lagged correlation (Mitra et al., 2014), Granger causality (Liang et al., 2021), transfer entropy (Besserve et al., 2015), or dynamic time warping-based similarity measures (Linke et al., 2020), could be substituted depending on the assumptions and requirements of a particular application. Once local propagation relationships have been established, the subsequent graph construction, wave decomposition, and spatiotemporal tracking procedures remain unchanged. Different edge-defining metrics may offer different trade-offs between computational cost, detection sensitivity, and specificity, but all can be incorporated within the same graph-based framework.

Finally, the graph-based algorithm is capable of identifying multiple simultaneously occurring TWs, even when their spatial extents partially overlap—a task that remains challenging for most existing methods.

#### 3.1.2 Limitations

Our algorithm has three primary limitations. First, it assumes that each TW originates from or terminates at a local source or sink within the recorded area. For TWs whose source or sink lie just outside the recording boundary, border electrodes may still serve as a good approximation. However, the “rooted” wave assumption fails for pure planar, rotational, or saddle waves. Such patterns may be more common in recording systems with limited coverage, where radial waves originated from a distant source can appear planar and where interactions between oppositely directed waves may resemble rotational or saddle waves resulting from waves shearing or collision, respectively. Examples of these patterns in the datasets of Townsend et al. (2015) and Das et al. (2026) are often anisotropic, suggesting that they may arise from interactions among multiple waves rather than representing isolated patterns. In such cases, our algorithm can still successfully decompose the global structure into combinations of rooted directed graphs. Nevertheless, because the primary purpose of identifying root nodes is to partition the global phase map into individual TWs, smaller recording systems that typically contain only one dominant TW may skip this step and analyze the global phase gradient graph (and its MDST) directly. Second, like many methods relying on linear-linear or circular-linear correlation, our algorithm assumes constant propagation velocity for each TW. In reality, TWs may propagate anisotropically and change velocity across direction and cortical regions. Among the three major TW models, both the single oscillator and the excitable network models predict that propagation velocity is determined solely by conduction delay of neuronal fibers (Ermentrout and Kleinfeld, 2001). Under this framework, conduction delays of each brain region should remain relatively stable across experimental timescales and could be estimated from repeated TW observations. Because our algorithm provides fine spatial granularity, it could incorporate such information in future implementations, potentially improve the reliability of correlation statistics. Third, our algorithm aggressively maximizes the detection and spatiotemporal extent of TWs whenever theoretically permissible. As shown in Supplementary Videos S1, S2, even pure noise can occasionally generate TW-like patterns that are detected by our algorithm. However, these spurious TWs are limited in spatiotemporal extent and statistical significance. Consequently, it remains essential to evaluate the event-locked inter-trial consistency of detected TWs as well as the biological plausibility of their propagation patterns and wave parameters, as performed in the present study.

Finally, although not a limitation of the algorithm itself, we acknowledge that our implementation in MATLAB may not be easily reproducible for users without its access. Future work could involve porting this framework to open-source alternatives, such as Python or GNU Octave, to broaden accessibility.

### 3.2 The post-saccadic traveling wave

#### 3.2.1 Comparison with previous results

At first glance, our physiological findings may appear similar to those reported previously by Kaneko et al. (2022). However, the two studies differ in several fundamental aspects. First, they focused on theta-band activity (8 Hz) whose phase exhibited the strongest coupling to high-gamma power in their data. By contrast, we focused on high-beta to low-gamma activity (20–50 Hz), which in our data showed the strongest phase-locked power, closely corresponded to the ERP, and was systematically modulated by saccade directions. We additionally examined the spatiotemporal organization of 4–12 Hz phase activity in our recordings but failed to reproduce their observations, potentially due to differences in experimental conditions (visually-guided saccade toward simple stimuli versus free-viewing of natural images). Second, although not explicitly discussed in their study, the propagation velocity of the theta waves can be estimated from snapshots of their phase map to be no more than a few hundred mm/s. This is substantially slower than the velocities observed in our data (1,000–2,000 mm/s) and may therefore reflect distinct propagation mechanisms from those mediated by myelinated white matter fibers. Third, their analysis began from and ended with the averaged phase maps, potentially obscuring wave properties such as onset timing, amplitude, and phase gradient. By contrast, the graph-based algorithm enabled TW identification on a trial-by-trial basis, allowing refined quantification of wave properties.

#### 3.2.2 Physiological interpretations

Many primate studies have reported perisaccadic modulations in cortical visual areas, including V1 (Rajkai et al., 2008; Sato et al., 2012; McFarland et al., 2015), V2 (Purpura et al., 2003), V4 (Zanos et al., 2015), MT (Ibbotson et al., 2007), and temporal area TE (Purpura et al., 2003). However, it remained unclear whether these modulations arise independently within each cortical area or are coordinated across areas through a shared mechanism. Our data suggested that the macroscopic TW originated from V1 may account for a substantial portion of these peri-saccadic modulations reported previously. There are four possible interpretations of the post-saccadic TWs observed in this study: (1) responses to target onset, (2) responses to target foveation, (3) responses to retinal sweep of the target during saccades, or (4) corollary discharge associated with saccade planning. Each possibility is discussed below.

The first interpretation appears unlikely. Given the large variability in SRT (55 to 200 ms), TW latencies aligned to saccade offset would not be as tightly clustered within the observed interval (−10 to 40 ms) if they were time-locked to target onset. Furthermore, the absence of a clear relationship between SRT and TW latency argues against this interpretation (Supplementary Figure S7). The second interpretation predicts similar responses regardless of target location. However, our results showed that TW latency (Supplementary Figure S6), source location (Figure 7 and Supplementary Figure S8), direction of propagation (Figure 8), and other wave properties (Figure 9 and Supplementary Figure S10) were all modulated by saccade direction. To exclude the possibility that these modulations simply reflect variability in saccadic landing location, we repeated the analysis using only overshooting saccades and obtained qualitatively similar results (Supplementary Figure S9). The third interpretation predicts that TWs would originate from the retinotopic representation of the target stimulus and propagate toward the foveal representation as the target is brought onto the fovea during the saccade. This prediction closely matched our observation. Following contraversive and downward saccades, TWs originated from caudomedial and dorsomedial regions of V1, respectively, consistent with the retinotopic representations of target stimuli. In addition, the caudal-to-rostral propagation direction of the TWs corresponded to the peripheral-to-central trajectory of the target’s representation within V1. The response latency of V1 neurons can be as short as 20–25 ms (Bair et al., 2002), which is consistent with the onset timing of the observed TWs given an average saccade duration of ~30 ms. Our findings may appear inconsistent with observations by Zanos et al. in area V4 (Zanos et al., 2015), who reported waves propagating from foveal toward peripheral representations. However, because the foveal representation of marmoset V4 is located posterodorsally whereas peripheral representation is located anteroventrally (Solomon and Rosa, 2014), their observation are in fact compatible with the caudal-to-rostral wave propagation observed in our study. The fourth interpretation involves corollary discharge related perisaccadic modulation. Such inactivation-reactivation peri-saccadic activity has been extensively reported in humans (Skrandies and Laschke, 1997) (referred to as “lambda waves”), macaques (Purpura et al., 2003; Rajkai et al., 2008; McFarland et al., 2015), and marmosets (Kaneko et al., 2022). Despite modulated by visual reafferent signals, this activity persists even under conditions that minimize displacement of retinal input or under total darkness (Rajkai et al., 2008; McFarland et al., 2015). The reduced sensitivity of V1 neurons during the inactivation phase (McFarland et al., 2015), together with subsequent increases of neuronal excitability in V1 (Rajkai et al., 2008) and the middle temporal area (Ibbotson et al., 2007), linked this peri-saccadic activity to saccadic suppression and its recovery. Moreover, previous studies have demonstrated modulation of this peri-saccadic activity by saccade direction (Purpura et al., 2003), consistent with our findings. Nevertheless, both the third and fourth interpretations predict stronger temporal alignment to saccade onset than was observed in our data. Determining whether the observed TWs primarily reflect retinal sweep, corollary discharge, or a combination of both will require more targeted experiments. Regardless of the initiating mechanism, the TW may serve as a means of sequentially resetting cortical excitability across visual areas in a hierarchical order, thereby preparing the visual system for incoming visual input upon fixation.

## 4 Conclusion

The primary contribution of this study is providing a graph-based framework that efficiently decomposes complex large-scale phase patterns into multiple simultaneously occurring TWs in non-planar recording systems. We implemented this framework as an algorithm that successfully identified and quantified macroscopic TWs originating in V1 following saccades in hemispheric ECoG recordings from marmosets. The framework is flexible and modular, and can incorporate alternative measures of local propagation depending on the recording modality and computational requirements. Combined with large-scale electrophysiology or neuroimaging datasets, the graph-based framework introduced here serves as a general approach for investigating dynamic inter-areal communication and coordination mediated by TWs, which may in turn shape perception and behavior.

## 5 Materials and method

### 5.1 Animal preparation

Two adult common marmosets (*Callithrix jacchus*) were chronically and epidurally implanted with hemispheric electrocorticogram (ECoG) arrays. Marmoset S (male, 2y3m, 370 g) was implanted on the right hemisphere with a 96-channel ECoG array developed by Komatsu et al. (2019). Marmoset L (female, 3y1m, 380 g) was implanted on the left hemisphere with a custom-designed 128-channel ECoG array (Cir-Tech Co., Ltd.). Both ECoG arrays were made of polyimide, with 0.8 mm electrode diameter and 2–2.5 mm inter-electrode distance. The surgeries were planned based on computerized tomography (CT) and magnetic resonance (MR, Marmoset S: 3 T, Marmoset L: 7 T) imaging. Due to individual differences in brain and skull shape, some electrodes were cut before surgery for easiness of implantation. During the surgery, two slit-shaped craniotomies were opened, one close to the midline and one close to the border of the temporal muscle. The array arms were inserted into the medial slit and adjusted through the lateral slit to avoid overlapping. The connector box (combined with the headpost) was glued to the skull and seven skull skews by Super-Bond (Sun Medical Co., Ltd) and strengthened by Unifast II dental resin (GC Corporation). Both the connector box and the skull screws were made of polyether ether ketone (PEEK). All surgeries were performed under general anesthesia (isoflurane 1.5%). Analgesics (ketoprofen 1 mg/kg) was administered after surgery.

### 5.2 Electrode localization

CT images (resolution: 0.25×0.25×0.25 mm for Marmoset S, 0.12×0.12×0.12 mm for Marmoset L) were taken after ECoG implantation and aligned with MR images (resolution: 0.30 × 0.30 × 0.30 mm for Marmoset S, 0.18 × 0.18 × 0.18 mm for Marmoset L) taken before surgery. Electrode locations were manually labeled on the CT images. The MR images were registered to the BMA 2019 *Ex Vivo* v1.0.0 atlas (Woodward et al., 2018) using 3D Slicer and the Advanced Normalization Tools (ANTs) (Avants et al., 2011) implemented in 3D Slicer. The resulting transformations were applied to the electrode labels, and the covered brain areas were determined by the vertices of the atlas nearest to the electrode labels.

### 5.3 Data acquisition

Local field potential (LFP) was acquired and digitized at 30 kHz using hardware and software from SpikeGadgets (headstage: UH32; control units: MCU, HCU, ECU; software: Trodes; SpikeGadgets LLC). The LFP data was synchronized with task events by a digital line connecting the ECU and a USB DAQ system (USB1208FS, Measurement Computing Corporation) wired to the experimental computer. LFP was down-sampled to 1,000 Hz by average-based decimation before processing. No further filter or referencing was applied unless specified otherwise. Overlapping, cut, or damaged electrodes were excluded from the analysis, so that 82 and 122 valid electrodes remained for Marmoset S and Marmoset L, respectively.

Eye tracking data was acquired by Eyelink 1000 plus primate mount system (SR Research, Canada) at 500 Hz binocularly in pupil-CR mode. The eye data was synchronized with task events by messages sent to the Eyelink host PC and stored in the EDF file. The eye data was linearly up-sampled to 1,000 Hz before processing.

All data analysis was done using custom MATLAB (R2024a, MathWorks) codes and C/C++ codes implemented through MATLAB MEX functions unless specified otherwise.

### 5.4 Behavioral paradigm

The experimental setup was the same as our previous study (Chen et al., 2021), if not specified otherwise. Experiments were performed in a dark sound-attenuating chamber. Visual stimuli were presented at 60 Hz on a 1,920 × 1,080 pixel LCD monitor (Dell AW2521HF), placed at 410 mm from the subject. The display area of the monitor spans 67.1×40.5 degree visual angles (DVA), but only a 30×30 DVA gray window (luminance: 74.6 cd/m^2^) centered at the monitor center was used as stimulus background (the area outside this window was black). Two photodiodes were placed at the top left and bottom left corners of the monitor, and connected to the experimental computer via the USB DAQ system. All tasks were written in Python using the PsychoPy library (Peirce et al., 2019) for the presentation of visual stimuli. During the task, white squares were presented on both corners, which flashed off for one frame at stimulus onset, so the precise timing could be detected by the photodiodes. The flashed area and the photodiodes were covered by black shrouds and were not visible to the subject. Adaptive Sync technology was applied to minimize display latency and enhance the consistency of frame interval (Poth et al., 2018). The average display latency measured by the photodiodes is about 2.5 ms, and the standard deviation of the frame interval is about 0.1 ms. The marmosets were trained to be head-fixed in a primate chair while staying calm. The chair has 6 degrees of freedom for adjustment of head position. Custom coordinates were used for each marmoset so that they look at the monitor center when relaxed.

In the visually-guided saccade task, a full-contrast, bright-center-dark-surround annulus stimulus (center: 387.2 cd/m^2^, outer diameter: 2 DVA) was shown at the monitor center. The marmosets were required to fixate on the stimulus before reaching a 1,000 ms timeout period, and kept fixation for 250 ms. Then the stimulus jumped to a peripheral location (eccentricity: 4, 5, 6, 7, 8, 9 DVA; direction: 0°, 45°, 90°, 135°, 180°, 225°, 270°, 315°), where the marmosets were required to make a saccade before reaching a 1,000 ms timeout period, and fixate for 250 ms. The inter-trial interval (ITI) was 2,000 ms. If successful, the stimulus was replaced by a picture of a marmoset face (2 × 2 DVA square) staying for the first 500 ms of the ITI, and a liquid reward was delivered. A fixation break or timeout would terminate the trial without reward. An invisible eye mask (diameter: 2 DVA) was used to determine the overlapping of gaze location with the stimulus. Stimulus eccentricity remained constant and stimulus direction was pseudo-randomized in each task session. After each failed trial, the same stimulus location was used in the next trial. Each task session contained 24 required trials (3 for each direction) and was aborted if the number of failed trials reached 24. The length of eye data and ECoG data sessions were the same as the corresponding task sessions.

The same task was used for daily gaze calibration. Calibration was done separately for both eyes and both axes by Equation 1:


$$y=\tan^{-1}(a\cdot x+b)$$ (1)

where *y* is the target location relative to each eye in DVA, *x* is the mean raw gaze location in the last 50 ms of fixation in arbitrary unit, and *a* and *b* are the fitting parameters. Although the maximum tolerated fixation error was 2 DVA by task design, the marmosets usually made precise fixations and the root-mean-square-error of daily gaze calibration was only about 0.6 DVA.

### 5.5 Saccade detection

Saccade periods were labeled using the U′n′Eye deep neural network (Bellet et al., 2019) in Python, trained by manually labeled eye data from three separate marmosets. Eye velocity for the input to this network was calculated by a differentiating odd symmetric FIR filter with a smoothing 3 dB cutoff at 54 Hz (Hafed et al., 2009). Samples of labeled data were manually checked for each marmoset to ensure model generalizability. Only successful trials with a single saccade from the screen center to the target were used for analysis. Trials with saccade reaction time no longer than 55 ms were regarded as anticipatory and excluded from analysis.

### 5.6 Event-related potential (ERP) analysis

LFPs of pure ipsiversive (Marmoset S, *n* = 1,355; Marmoset L, *n* = 1,168) and pure contraversive (Marmoset S, *n* = 1,281; Marmoset L, *n* = 1,046) trials were aligned on saccade offset to calculate the averaged ERP. For feature analysis of the triphasic ERP (Supplementary Table S2), the timing of the trough (Trough), the first peak (Peak 1), and the second peak (Peak 2) were determined for each trial. We defined the minimum during 20 to 60 ms following saccade offset as Trough, the maximum from saccade offset to Trough as Peak 1, and the maximum from Trough to 120 ms following saccade offset as Peak 2. For the same analysis on ERP aligned on saccade onset (Supplementary Table S3), different search windows were used: 50 to 90 ms for Trough, 30 ms to Trough for Peak 1, and Trough to 150 ms for Peak 2. If any of the extrema was not a local extrema (i.e., found at the edges of the search window), the trial was excluded from this analysis.

Statistics were performed to compare ERP features from two conditions (Contra. vs. Ipsi. in Supplementary Table S2; Sacc. On. vs. Sacc. Off in Supplementary Table S3). Median latency and amplitude were compared by the two-tailed Wilcoxon rank sum test. The mean absolute deviation from the median (MADM) of latency was compared by the Brown–Forsythe test.

### 5.7 Time-frequency analysis

Time-frequency representation (TFR) of the LFP signal was calculated by the wavelet convolution method (Equations 2–4) using complex Morlet wavelets (MW) of 64 central frequencies logarithmically spaced between 1 Hz and 160 Hz. The process was applied to the whole recording session, which was zero-padded to 2^18^ samples, before dividing into trials. To achieve a balance between temporal and spectral precision across different frequencies, a fixed wavelet number of 7 was used instead of a fixed time window.


TFR(t,f)=LFP(t)∗MW(t,f) (2)

MW(t,f)=1sπ⋅e−t22s2⋅ei2πft (3)

$$s=\frac{7}{2\pi \cdot f\cdot f_{s}}$$ (4)

where *s* is the standard deviation of the Gaussian taper, *f_s_* is the sampling frequency (here, 1,000 Hz), *t* is time, *f* is the central frequency of the Morlet wavelet, and *to the same symbol as Equation 2. The Morlet wavelets were then normalized by their area under the curve (i.e., summation), so the amplitude of the resulting TFR reflects that of the original LFP.

Power was calculated by squaring the absolute value of the TFRs (Supplementary Figure S5A), which was then *Z*-scored by the mean and standard deviation of each recording session (Supplementary Figure S5B), for each electrode and frequency.

The inter-trial phase clustering index (ITPC, Supplementary Figure S5C) at time *t* and frequency *f* was calculated by Equation 5:


$$\text{ITPC}(t,f)=|\frac{\sum_{k=1}^{n}e^{i\varphi_{k}(t,f)}}{n}|$$ (5)

where *n* is the total trial number, *k* is the trial ID, *i* is the imaginary unit, and *φ_k_*(*t*, *f*) is the phase angle of *TFR*(*t*, *f*) in the *k*th trial. By definition, ITPC ranges between 0 and 1, with 0 indicating random phase distribution and 1 indicating perfect phase locking. Phase-locked power was calculated as the product of normalized power and ITPC.

### 5.8 Instantaneous amplitude, phase, and frequency

For each electrode in the ECoG array, its original LFP time series was temporally filtered with a zero-lag 8th-order digital Butterworth band-pass filter (20–50 Hz). The complex analytic signal *X(t)* can be derived from from Equation 6:


X(t)=x(t)+iH[x(t)]

=A(t)(cos(φ(t))+isin(φ(t))) (6)

where *i* is the imaginary unit, *H*[*x*(*t*)] denotes the Hilbert transform of the filtered signal *x*(*t*), *A*(*t*) and *φ*(*t*) denote its instantaneous amplitude and phase, respectively. *φ*(*t*) segments with negative frequency were interpolated by a similar procedure as Davis et al. (2020), so that the linearized phase increases monotonically.

For each *t*, find *t_before_* and *t_after_* by linear interpolation to satisfy Equation 7:


{φ(tbefore)=φ(t)−π4φ(tafter)=φ(t)+π4 (7)

, so *t_after_* - *t_before_* is roughly a quarter of a period (*π*/2), over which we assume the wave is stationary. We define the instantaneous period *p_i_*(*t*) by Equation 8:


$$p_{i}(t)=4(t_{\text{after}}-t_{\text{before}})$$ (8)

Instantaneous period values beyond the theoretical limits (20 and 50 ms, based on cutoff frequencies of the filter) were replaced by the limits when deriving algorithm parameters, but not when calculating the instantaneous frequency.

### 5.9 Algorithm for wave detection

The proposed framework consists of four stages:

#### 5.9.1 Stage 1: neighbor graph construction

This stage builds a sparse graph connecting nearby electrodes. A weighted neighbor graph *G_n_* = (*V*(*G_n_*), *E*(*G_n_*)) was constructed for a given electrode map. Each node in *V*(*G_n_*) represents a valid electrode, and each edge in *E*(*G_n_*) connects a neighboring electrode pair, weighted by the geodesic distance on the brain surface mesh (hereinafter, “distance”) between the electrodes. To connect only immediately neighboring electrodes, we designed an algorithm as follows:

(1) Construct an edge list including all electrode pairs whose distances were less than 6 mm. Sort the edge list in order of decreasing weight.
(2) For each edge *e_i_* in the sorted list, do the following:
(3) Check for each of its adjacent edges *e_j_*. If the angle of *e_i_* and *e_j_* in the Euclidean space was smaller than 22.5°, and their weight ratio (larger divided by smaller) was greater than 1.1, remove edge *e_i_* from the list (see Supplementary material 1.2.1). By this, if two edges are adjacent (i.e., share a vertex), have a similar orientation, and differ substantially in length, then the longer edge is regarded as geometrically redundant and removed.
(4) Put all the remaining edges in the list to *E*(*G_n_*).

The above procedures effectively reduced the number of edges to be processed in later stages by over 90% (Supplementary Table S5).

#### 5.9.2 Stage 2: phase-gradient graph construction

This stage estimates local propagation relationships between neighboring electrodes and encodes them as directed edges. For the data at time point *t*, the instantaneous phase and period of each electrode were calculated using the above-mentioned method (Equations 6–8). Then, a directed weighted phase gradient graph *G_pg_*(*t*) = (*V*(*G_pg_*(*t*)), *E*(*G_pg_*(*t*))) was constructed. The node set V(*G_pg_*(*t*)) was the same as *V*(*G_n_*) and remained constant throughout this stage (i.e., including all valid electrodes, connected or not). Each directed edge in *E*(*G_pg_*(*t*)) represents wave flow between a neighboring electrode pair, determined by screening through each edge *e*(*a*, *b*) in *G_n_* by the following steps:

1) If both of the following criteria were met (Equations 9–14), proceed; otherwise, skip this edge:

$$\mathrm{PR}(t,a,b)<PR_{\max}$$ (9)

$$\mathrm{PR}(t,a,b)=\frac{\max(p_{i}(t,a),p_{i}(t,b))}{\min(p_{i}(t,a),p_{i}(t,b))}$$ (10)

$$PR_{\max}=1.25$$ (11)

where *PR*(*t*, *a*, *b*) is the period ratio of the electrode pair and *PR_max_* is the maximum period ratio allowed (see Supplementary material 1.2.2).


$$\mathrm{PG}(t,a,b)<PG_{\max}(t)$$ (12)

$$\mathrm{PG}(t,a,b)=\frac{|\arg(X(t,a)\bar{X}(t,b))|}{\text{dist}(a,b)}$$ (13)

$$PG_{\max}(t)=\frac{2\pi \cdot f_{s}}{\min(p_{i}(t,a),p_{i}(t,b))\cdot V_{\min}}$$ (14)

where *PG*(*t*, *a*, *b*) is the phase gradient of the electrode pair, the bar above *X*(*t*, *b*) denotes its complex conjugate, *dist*(*a*, *b*) is the distance between the electrode pair, and *PG_max_*(*t*) is the maximum phase gradient allowed, with *V_min_* = 500 mm/s (see Supplementary material 1.2.3).

2) Calculate the mean absolute phase difference (MAPD) between the fragment of *φ_a_*(*t*) centered at time *t*, whose length (2*w* + 1) was around a quarter of the period, and a fragment of *φ_b_*(*t*) with the same length but time-shifted by *d_max_* + 1 samples at most (Equations 15–17), where *d_max_* is the maximal delay in sample, determined by the distance of the pair and minimal wave velocity (Equation 18) (see Supplementary material 1.2.3 and Supplementary Figure S2):

$$\mathrm{MAP}D_{\mathrm{ba}}(d)=\frac{\sum_{u=t-w}^{t+w}|\arg(X(u,a)\bar{X}(u+d,b))|}{2w+1}$$ (15)

$$w=\text{round}(\max(p_{i}(t,a),p_{i}(t,b))/8)$$ (16)

$$d\in N,|d|\leq d_{\max}+1$$ (17)

$$d_{\max}=\text{ceil}(\frac{\text{dist}(a,b)\cdot f_{s}}{V_{\min}})$$ (18)

Similarly, calculate *MAPD_ab_*. The time shifts that give the minimal *MAPD_ba_* and *MAPD_ab_* were *d_ba_* and *d_ab_* respectively (Equations 19, 20):


$$d_{\mathrm{ba}}=\text{argmin}(\mathrm{MAP}D_{\mathrm{ba}})$$ (19)

$$d_{\mathrm{ab}}=\text{argmin}(\mathrm{MAP}D_{\mathrm{ab}})$$ (20)

3) If all of the following criteria were met, proceed; otherwise, skip this edge:

I *MAPD_ba_*(*d_ba_*) and *MAPD_ab_*(*d_ab_*) must also be local minima in the search range (Equation 21):

$$d_{\mathrm{ba}},d_{\mathrm{ab}}\neq \pm (d_{\max}+1)$$ (21)

II *d_ba_* and *d_ab_* must be symmetrical around zero, with a tolerance of 1 sample (Equation 22):

$$|d_{\mathrm{ba}}+d_{\mathrm{ab}}|\leq 1$$ (22)

III Both MAPDs must be no greater than a threshold, determined by the smaller period and a multiplier (Equations 23, 24) (see Supplementary material 1.2.4):

$$\mathrm{MAP}D_{\mathrm{ba}}(d_{\mathrm{ba}}),\mathrm{MAP}D_{\mathrm{ab}}(d_{\mathrm{ab}})\leq \mathrm{MAP}D_{\max}$$ (23)

$$\mathrm{MAP}D_{\max}=\frac{1.75\pi }{\min(p_{i}(t,a),p_{i}(t,b))}$$ (24)

4) Add edges to *E*(*G_pg_*) according to Equations 25–27:

I If

$$\left\{ \begin{array}{c} d_{\mathrm{ab}}<0\\ d_{\mathrm{ba}}>0 \end{array}\right.$$ (25)

the past phase of electrode *a* fits well with the current phase of electrode *b*, and the current phase of electrode *a* fits well with the future phase of electrode *b*. Add *e*(*a*, *b*), weighted by *PG*(*t*, *a*, *b*).

II Similarly, if

$$\left\{ \begin{array}{c} d_{\mathrm{ab}}>0\\ d_{\mathrm{ba}}<0 \end{array}\right.$$ (26)

add *e*(*b*, *a*), weighted by *PG*(*t*, *b*, *a*).

- III If

$$\left\{ \begin{array}{c} d_{\mathrm{ab}}=0\\ d_{\mathrm{ba}}=0 \end{array}\right.$$ (27)

the delay of the putative wave flow between electrodes *a* and *b* is less than 1 sample. In this case, both *d_ab_* and *d_ba_* were replaced by 1 or −1, whichever gave a smaller MAPD. Then check the criteria above again.

#### 5.9.3 Stage 3: wave parcellation

This stage extracts connected propagation structures, and retains statistically coherent traveling waves. Find the root node set *R*(*t*) ⊆ *V*(*G_pg_*(*t*)), which contains nodes that have no inedge and at least one outedge. For each *r* ∈ *R*(*t*), construct an induced subgraph *G_sub_*(*t*, *r*) of *G_pg_*(*t*) containing root node *r* and nodes that *r* has a directed path to. *F*(*t*) is the set containing all such rooted subgraphs at time *t*. For each *G_sub_*(*t*, *r*) in *F*(*t*), do the following:

1) We assume a wave involves at least 3 electrodes, so remove Gsub(t,r) if Equation 28:

$$|V(G_{\mathrm{sub}}(t,r))|<3$$ (28)

2) Calculate the maximum directed spanning tree *MDST*(*t*, *r*) of *G_sub_*(*t*, *r*) by the Chu–Liu/Edmonds’ algorithm (Edmonds, 1967). Find the unique path from *r* to each node in *V*(*G_sub_*(*t*, *r*)). The weight of such a path in *G_n_* represents the traveling distance from the source electrode. The cumulative phase difference of such a path can be derived by summing up the phase difference of its constituent edges, which can be derived by multiplying their weights in *G_pg_*(*t*) and *G_n_*. Then, calculate the *p*-value of Pearson correlation between the traveling distance and the cumulative phase difference. If the *p*-value is greater than 0.005 (see Supplementary material 1.2.5), remove *G_sub_*(*t*, *r*).

#### 5.9.4 Stage 4: temporal tracking

This stage associates wave structures across adjacent time points to obtain complete spatiotemporal TW representations.

Create a list *TW* to store the spatiotemporal representation of the identified finished waves, and a list *TW_temp_* to store the ongoing waves. Each of such representations is formed by concatenating *G_sub_*(*t*, *r*) representing the same wave across consecutive time points. For each *F*(*t*), do the following:

1) Mark the latest subgraph of each wave in *TW_temp_* as active. Sort *F*(*t*) in order of decreasing |*V*(*G_sub_*(*t*, *r*))|. For each *G_sub_*(*t*, *r*) in *F*(*t*), compare with the active subgraphs *G_sub_*(*t’*, *r’*) in *TW_temp_* by the following rules Equations 29–32:
  Case 1: If

$$\left\{ \begin{array}{c} t-t^{\prime }=1\\ r=r^{\prime } \end{array}\right.$$ (29)

append *G_sub_*(*t*, *r*) after *G_sub_*(*t’*, *r’*) as the last frame of the wave, and mark *G_sub_*(*t’*, *r’*) as inactive.

Case 2: If


$$\left\{ \begin{array}{c} t-t^{\prime }>1\\ r=r^{\prime } \end{array}\right.$$ (30)

or


$$\left\{ \begin{array}{c} t-t^{\prime }=1\\ e(r,r^{\prime })\in E(\mathrm{Gn}) \end{array}\right.$$ (31)

calculate the similarity index (SI) between *G_sub_*(*t*, *r*) and *G_sub_*(*t’*, *r’*) as follows:


$$\mathrm{SI}=\frac{|V(G_{\mathrm{sub}}(t,r))\cap V(G_{\mathrm{sub}}(t^{\prime },r^{\prime }))|}{\min(|V(G_{\mathrm{sub}}(t,r))|,|V(G_{\mathrm{sub}}(t^{\prime },r^{\prime }))|)}$$ (32)

If *SI* ≥ 0.75 (see Supplementary material 1.2.7), append *G_sub_*(*t*, *r*) after *G_sub_*(*t’*, *r’*) as the latest subgraph of the wave, and mark *G_sub_*(*t’*, *r’*) as inactive.

Case 3: If not appended to any wave in *TW_temp_*, let *G_sub_*(*t*, *r*) be the first subgraph of a new wave in *TW_temp_*.

3) Find waves in *TW_temp_* that had not been renewed for longer than a maximal interruption threshold of 3 ms (see Supplementary material 1.2.6), and move them to *TW*.

### 5.10 Wave simulation

Traveling waves of known parameters were simulated on the electrode maps of the two marmosets. First, 1–4 source electrodes located on occipital, frontal, parietal, and temporal areas were manually selected (see Supplementary Figure S3; electrode map of Marmoset S: 80, 60, 30, 10; electrode map of Marmoset L: 69, 58, 45, 31; only the first two electrodes in the list were used in the two source condition, and so on). Each of them independently generated traveling waves with a random duration (uniformly distributed in 2–4 cycles) at random intervals (uniformly distributed in 2–4 cycles). For the wave from electrode i , its source signal is given by Equation 33:


$$s_{i}(t)=A\cos(\frac{2\pi \omega_{i}t}{f_{s}}+\theta_{i}),t_{0}\leq t\leq t_{\mathrm{end}}$$ (33)

where *A*, *ω_i_*, and *θ_i_* denote the amplitude (unity for all waves), frequency (uniformly distributed in 20–50 Hz), and phase shift (uniformly distributed in 0-2π) of the signal, respectively; *fs* denotes the sampling frequency (1,000 Hz), *t_0_* and *t_end_* denote the onset and offset timing of the wave. 10,000 waves were generated for each combination of source number and noise level.

To determine the spatial scope of each simulated wave, the shortest path tree from each source node was constructed on the neighbor graph. Then a probabilistic depth-first search (pDFS) algorithm was conducted on the shortest path tree from the source node. During the pDFS, the probability for a newly encountered node to be pushed to the stack is described by a Gaussian cumulative distribution function (GCDF) based on its graph distance from the source (Equation 34):


P(i,a)=1−GCDF(μ,σ,dist(i,a)) (34)

where *μ* (here, 10 mm) and *σ* (here, 5 mm) denote the mean and standard deviation of the Gaussian distribution respectively, and *dist*(*i*, *a*) denotes the graph distance between source node *i* and node *a* in the neighbor graph. The parameters of pDFS were selected to make the spatial scope of the simulated waves resemble our real data. Nodes whose graphic distances were shorter than a threshold (here, 3.5 mm) were pushed to the stack regardless of the probability, to ensure a minimum number of member nodes around the source.

The signal at electrode *a* is given by (Equation 35):


$$x_{a}(t)=\sigma_{G}\varepsilon (t)+\sum_{i=1}^{n}s_{i}(t-\frac{\text{dist}(a,i)}{v_{i}})\cdot \gamma^{\text{dist}(i,a)}$$ (35)

where *n* denotes the number of simultaneously occurring waves at time *t* that encompass *a*, *v_i_* denotes the traveling velocity (uniformly distributed in 600–3,000 mm/s) of the wave, *dist*(*a*, *i*) denotes the graph distance between electrode *a* and source *i* in the neighbor graph, *σ_G_* denotes the standard deviation (10–50%) to be applied on a unit Gaussian noise *ε*(*t*), and *γ* denotes the attenuation factor of the signal (here, 0.99 mm^−1^). When *n* = 0, the noise standard deviation becomes 50% to represent the amplitude of non-TW dynamics during inter-wave-intervals.

Then, the graph-based algorithm was applied to the simulated data. For each simulated wave, we extracted the identified waves temporally overlapping with the simulated wave, whose main root node matched the source electrode of the simulated wave. The labeled timepoints of these identified waves were compared to the ground truth to calculate the temporal true positive rate (TPR) and false discovery rate (FDR). Member nodes existing in more than 50% of the labeled timepoints were defined as the spatial scope of the wave and compared to the ground truth to calculate the spatial TPR and FDR. Source amplitude was defined as the temporal median of the amplitude of the analytic signal at the root node over the lifespan of the wave. To calculate node frequency, instantaneous frequency of member nodes (based on the spatial scope defined above) at each timepoint over the lifespan of the wave were concatenated as an array, whose median was used. Edge velocity is calculated in a similar manner, replacing node frequency with the velocity estimated on each edge using Equation 36.


$$V(t,a,b)=\frac{2\pi \cdot f_{s}}{\text{mean}(p_{i}(t,a),p_{i}(t,b))\cdot \mathrm{PG}(t,a,b)}$$ (36)

### 5.11 Statistical analysis of wave parameters

For the ERP analysis, time-frequency analysis, phase map, and edge asymmetry analysis, all successful trials with single answering saccade and reaction time no less than 55 ms were included (sample size; Marmoset S, 0°: 1355, 45°: 1350, 90°: 1238, 135°: 1248, 180°: 1281, 225°: 1357, 270°: 1024, 315°: 1377; Marmoset L, 0°: 1046, 45°: 1027, 90°: 1063, 135°: 1104, 180°: 1168, 225°: 1190, 270°: 1104, 315°: 1129).

For analysis on latency distribution (Figure 6 and Supplementary Figure S5), only waves originating from V1 surrounding saccade offset (−50 to 100 ms) with the largest spatiotemporal scope over the visual areas (see Supplementary Table S1) in each trial were included (sample size; Marmoset S, 0°: 1355, 45°: 1350, 90°: 1238, 135°: 1248, 180°: 1281, 225°: 1357, 270°: 1024, 315°: 1377; Marmoset L, 0°: 1045, 45°: 1027, 90°: 1063, 135°: 1104, 180°: 1168, 225°: 1190, 270°: 1104, 315°: 1129). The latency data was divided into 30 bins, each 5 ms wide. One-tailed binomial test against the chance level of 1/30 was performed for each bin. The probability of the largest waves occurring in −10 to 40 ms surrounding saccade offset was compared between pairs of saccade directions (0° vs. 180°, 90° vs. 270°, 45° vs. 135°, 315° vs. 225°) by Pearson’s chi-squared test.

For analysis on wave source (Figure 7, Supplementary Figures S8, S9), wave pattern (Figure 8, Supplementary Videos S8, S9), and other parameters (Figure 9, Supplementary Figures S10, S11), only trials with at least one identified TW occurring during −10 to 40 ms surrounding saccade offset were included (sample size; Marmoset S, 0°: 1301, 45°: 1290, 90°: 1194, 135°: 1209, 180°: 1250, 225°: 1329, 270°: 999, 315°: 1324; Marmoset L, 0°: 1009, 45°: 978, 90°: 1022, 135°: 1059, 180°: 1112, 225°: 1132, 270°: 1052, 315°: 1096). The source probability of each electrode between ipsiversive/contraversive and upward/downward conditions was compared by Pearson’s chi-squared test. To acquire the edge asymmetry, the probability for a wave flow to exist on each edge of the neighbor graph was calculated for each moment around the saccade offset from the phase gradient graphs. Then the probability of edges connecting the same two nodes with reversed directions was compared by Pearson’s chi-squared test. Edges with *p*-values below 0.001 were used to calculate the MDST shown in Figure 8. The median of wave duration, maximum of the covered electrode, median of source amplitude, spatiotemporal median of node frequency, and spatiotemporal median of edge velocity between pairs of saccade directions (0° vs. 180°, 90° vs. 270°, 45° vs. 135°, 315° vs. 225°) were calculated by the same method as the simulated waves and compared by the two-tailed Wilcoxon rank sum test.

## Data Availability

The datasets presented in this study can be found on Zenodo (DOI: 10.5281/zenodo.14020930).

## Glossary

TW: traveling wave
V1: primary visual cortex
LFP: local field potential
EEG: electroencephalography
ECoG: electrocorticography
MDST: maximum directed spanning tree
IP: instantaneous phase
MAPD: mean absolute phase difference
pDFS: probabilistic depth-first search
STD: standard error
TPR: true positive rate
FDR: false discovery rate
ERP: event-related potential
TFA: time-frequency analysis
ITPC: inter-trial phase clustering index
SRT: saccade reaction time
CT: computerized tomography
MR: magnetic resonance
DVA: degree visual angle
RGD: relative graph density
SI: similarity index
ICA: independent component analysis
