# Supplementary material for: Quantifying cortex-wide traveling brain waves of complex patterns with a graph-based algorithm
Source: Front Comput Neurosci. 2026 Aug 24;20:1844662. doi: 10.3389/fncom.2026.1844662 (PMC13547507; doi:10.3389/fncom.2026.1844662)
Supplement: Supplementary file 1 [file Data_Sheet_1.PDF]

Supplementary Material

1 Supplementary method

1.1 Pseudocode of the wave detection algorithm

1.1.1 Stage 1: Neighbor graph construction

Initialize graph G_n with given nodes and an empty set of edges

Identify all close neighbors

For each unique pair of nodes (a, b):

If the distance between a and b is less than 6 mm:

Add an undirected edge between a and b with a weight equal to their distance

Prune spatially redundant edges

Sort the edges of G_n in descending order of their weights

For each edge e_i in the sorted list:

For each subsequent edge e_j in the list:

If e_i and e_j share a node (are adjacent):

Let ang be the spatial angle between the two edge vectors in degrees

Let dr be the weight (i.e., distance) ratio of e_i to ej

Eliminate the longer edge if they point in a similar direction

If ($ang < 22.5$) and ($dr > 1.1$):

Remove e_i from the graph G_n

Break out of the inner loop (move to next edge e_i)

1.1.2 Stage 2: Phase-gradient graph construction

Initialize a directed graph $G_{pg}(t)$ with the same nodes as G_n and an empty set of edges

For each edge in G_n with end nodes [a, b]:

Quality control filtering

Calculate Period Ratio (*PR*(*t*, *a*, *b*)) between the instantaneous period values of *a* and *b* at time *t*

Calculate Phase Gradient (*PG*(*t*, *a*, *b*)) between *a* and *b* at time *t* as the phase difference divided by the geodesic distance

Calculate the dynamic threshold PG_{max} for the current pair (see *Supplementary Material 1.2*)

If (*PR*(*t*, *a*, *b*) >= *1.25*) or (*PG*(*t*, *a*, *b*) >= PG_{max}):

  Skip this edge and move to the next

Estimate optimal propagation delay via sliding window

Determine the maximum allowable delay value d_{max} based on geodesic distance and minimum propagation speed

For each integer delay *d* from ($-d_{max}$ - 1) to (d_{max} + 1):

  Compute the Mean Absolute Phase Difference (*MAPD*) over a temporal window 2**w*+1, centered at time *t*

  $MAPD_{ba}$: tracking signal from node *a* to delayed node *b*

  $MAPD_{ab}$: tracking signal from node *b* to delayed node *a*

Find the optimal delays (d_{ba} and d_{ab}) that minimize $MAPD_{ba}$ and $MAPD_{ab}$ respectively

Validation and boundary checks

Calculate the maximum allowable *MAPD* threshold

If either optimal delay hits the boundary ($-d_{max}$-1 or d_{max}+1)

or the delays are asymmetric around zero (abs(d_{ba} + d_{ab}) > 1)

or either minimum *MAPD* exceeds the threshold:

  Skip this edge and move to the next

Resolve zero-delay

If both d_{ba} and d_{ab} are exactly 0:

Change d_{ba} and d_{ab} to -1 or 1, whichever gives smaller *MAPD*

Build directed graph based on wave direction

Set edge weight to *PG*(*t*, *a*, *b*)

If wave flows from *a* to *b* ($d_{ab} < 0$ and $d_{ba} > 0$):

Add directed edge (*a* → *b*) to $G_{pg}(t)$

Else if wave flows from *b* to *a* ($d_{ab} > 0$ and $d_{ba} < 0$):

Add directed edge (*b* → *a*) to $G_{pg}(t)$

1.1.3 Stage 3: Wave parcellation

Identify all root nodes (sources) *R*(*t*) in $G_{pg}(t)$

Initialize an empty list *F*(*t*) to store verified subgraphs

For each root node *r* in *R*(*t*):

Find the subgraph $G_{sub}(t, r)$ reachable from *r* using a standard graph search

We only care about substantial waves

If G_{sub} contains 3 or more nodes:

Find the Maximum Directed Spanning Tree (*MDST*) of $G_{sub}(t, r)$

Calculate traveling distance (*TD*) of each node in *MDST*

Calculate cumulative phase difference (*CPD*) of each node in *MDST*

Verify if the wave propagation is linearly coherent

Let *P* be the Pearson correlation coefficient between *TD* and *CPD*

If $P < 0.005$:

Record the creation timestamp *t* and root *r* inside $G_{sub}(t, r)$

Append $G_{sub}(t, r)$ to list *F*(*t*)

1.1.4 Stage 4: Temporal tracking

Sort the current subgraphs $F(t)$ in descending order based on their node counts

Let TW and TW_{temp} be lists to store finished and ongoing waves respectively, whose elements are list of subgraphs representing waves time series

Mark the latest subgraphs of each wave in TW_{temp} as active

For each subgraph $G_{sub}(t, r)$ in $F(t)$:

  For each active subgraphs $G_{sub}(t', r')$ in TW_{temp}:

    # Case 1: Perfect consecutive time and root continuation

    If $(t == (t' + 1))$ and $(r == r')$:

      Append $G_{sub}(t, r)$ after $G_{sub}(t', r')$

      Mark $G_{sub}(t', r')$ as inactive for further matching in this step

      Break to process the next subgraph in $F(t)$

    # Case 2: Waves with minor spatial/temporal shifts but high overlap

    Else if $((t > (t' + 1))$ and $(r == r'))$ or $((t == (t' + 1))$ and IsEdge(G_n , $[r, r']$)):

      Calculate the similarity index (SI) between $G_{sub}(t, r)$ and $G_{sub}(t', r')$ (see *Supplementary Material 1.2*)

      If $SI >= 0.75$:

        Append $G_{sub}(t, r)$ after $G_{sub}(t', r')$

        Mark $G_{sub}(t', r')$ as inactive for further matching in this step

        Break to process the next subgraph in $F(t)$

    # Case 3: If $G_{sub}(t, r)$ matches no tracked wave after checking all active subgraphs in TW_{temp}, it becomes the start of a brand new wave

    Else:

      Append a new list started with $G_{sub}(t, r)$ to TW_{temp}

Clean up and archive finished waves

For each tracked wave in TW_{temp}:

If it has been inactive for more than 3 time steps:

Move it out of TW_{temp} and append it to the final traveling waves archive *TW*

1.2 Parameter selection

This section explains in detail how the parameters in the algorithm were designed and how the threshold values were selected. Most of the thresholds were determined based on knowledge about the waves of interest (e.g., frequency, velocity…). We further verified the robustness of the thresholds using real (-500 to 500 ms surrounding saccade offset, 100 trials randomly chosen for each marmoset) and shuffled (phase-randomized and spatially shuffled) data, assuming that real data contains much more TW-like patterns than shuffled data, which would have higher relative graph density (RGD, the number of edges with flow divided by the number of edges in the neighbor graph) with proper parameter set. Overall, our selection of parameters achieved ~7 times RGD contrast between real and shuffled data (Figure S12, Marmoset S: 0.53 vs. 0.08, Marmoset L: 0.57 vs. 0.08). The results were robust over different choices of trials. Although our empirical selection of parameters can serve as a good start point, we encourage users to perform similar selection procedure to find optimal parameters for their dataset and verify the robustness of those parameters.

1.2.1 When restricted to a small region, the alignment of electrodes is roughly grid-like, which means each of the eight neighboring electrodes spans ~45° of the central electrode, or 22.5° in clockwise and counterclockwise directions. Therefore, an electrode cannot be an immediate neighbor of the central electrode if there is another electrode in a similar direction but closer to the central electrode.

1.2.2 The maximal period ratio (PR_{max}) of 1.25 was empirically selected to well separate RGD between the real and shuffled datasets (Figure S12B, Marmoset S: 0.68 vs. 0.48, Marmoset L: 0.69 vs. 0.46).

1.2.3 The maximal phase gradient (PG_{max}) and maximal inter-electrode delay (d_{max}) were determined based on the assumption that the apparent wave velocity is never below 500 mm/s, which corresponds to a real wave velocity of up to 541 mm/s given a maximal direction deviation of 22.5° and well below the empirical velocity of macroscopic traveling waves(Hughes, 1995; Muller et al., 2018)(Hughes, 1995; Muller et al., 2018) (1000-10000 mm/s). These thresholds well separate RGD between the real and shuffled datasets (PG_{max} in

Figure S12A, Marmoset S: 0.88 vs. 0.42, Marmoset L: 0.85 vs. 0.38; d_{max} in Figure S12D, Marmoset S: 0.85 vs. 0.36, Marmoset L: 0.83 vs. 0.33).

1.2.4 Ideally, the minimal MAPD results only from the discrete sampling points not matching the actual delay (e.g., the sampling interval is 1 ms while the actual delay is 1.5 ms). In this case, the maximum of minimal MAPD happens when the real delay deviates from the nearest sample point by 0.5 sampling interval, resulting in an MAPD of:

$$2\pi \cdot f_i \cdot \frac{0.5}{f_s} = \frac{\pi}{p_i} \tag{1}$$

where f_i is the instantaneous frequency in Hz, f_s is the sampling frequency in Hz, and p_i is the instantaneous period in sample. By nature, waves with shorter periods tend to give higher minimal MAPD. Therefore, the $MAPD_{max}$ thresholds were dynamically determined to be a multiple of the theoretical maximum based on the period. A multiplier of 1.75 was empirically selected to well separate RGD between the real and shuffled datasets (Figure S12C Fig, Marmoset S: 0.79 vs. 0.39, Marmoset L: 0.79 vs. 0.37).

1.2.5 The Pearson P-value threshold of 0.005 was empirically selected to well separate RGD between the real and shuffled datasets (Figure S12F, Marmoset S: 0.64 vs. 0.37, Marmoset L: 0.71 vs. 0.40).

1.2.6 The interruption threshold was set to rescue occasional detection misses during the lifespan of a wave. When an interruption is not allowed, many short wave fragments (< 5 ms) were found near (≤ 3 ms) the preceding or succeeding fragments of the same roots (Figure S13). Given the temporal smoothing effect of the filter, it is unlikely for two independent 20-50 Hz waves to occur with such short intervals. Therefore, we set the interruption threshold to be 3 ms and view the fragments as a single wave.

1.2.7 Allowing subgraphs to append to waves with neighboring roots or non-continuous time improves flexibility, but can also give erroneous results. To quantify the similarity between the preceding and succeeding subgraphs, we designed the similarity index (SI) as the intersection size divided by the smaller size of the 2 subgraphs. Unlike the Jaccard index (intersection size divided by union size), our SI remains high when a wave grows or shrinks quickly, and therefore is more suitable for our purpose. For preceding subgraphs with the same root and continuous time, only a few (~0.5%) of the SI is below 0.75, but the ratio is much higher in cases of neighboring root or non-continuous time, which is even more obvious for the shuffled data (Figure S14).

1.3 Spiral wave simulation

Spiral-out waves are simulated on the brain surface mesh of the two marmosets. Vertex *c* near the temporoparietal junction is chosen as the spiral center. The spiral center *c* is not at any electrode position, because the phase at this singularity is undefined. Instantaneous phase of each remaining vertex *i* is determined by the following formula:

$$IP(i,t) = m \cdot ang(i,c) - pg \cdot dist(i,c) + 2\pi ft \quad (2)$$

where *m* is the spiral cycle number, *ang*(*i*,*c*) is the angle of vertex *i* around *c* in rad, *pg* is the radial phase gradient in rad/mm, *dist*(*i*,*c*) is the geodesic distance between *i* and *c*, *f* is the temporal frequency in cycle/ms, and *t* is time in ms.

Angles around a point can only be defined on a plane. To generalize the concept to a surface mesh, we do the following steps (see Figure S15A):

1) Find the tangent plane *PT*(*c*) of the surface mesh at vertex *c* (by MATLAB function "vertexNormal").
2) Let *i'* be the projection of *i* on *PT*(*c*), and $V_{ci'}$ be the vector from *c* to *i'*.
3) Let V_r be a reference vector, manually chosen to approximate the projection of the Sylvian fissure on *PT*(*c*) pointing caudodorsally, to mimic possible phase discontinuity caused by the fissure when *m* is not a whole number.
4) *ang*(*i*,*c*) is defined as the signed angle between V_{ci} and V_r.

In Video S3, we chose pg = 0.125 and f = 1/30 (~33.3 Hz), similar to empirical values in our ECoG data. We generated data for 200 ms, with gradually varying *m* from 1 to -1 (in 200 even steps), to simulate a transition from a clockwise spiral wave to a pure expanding wave and eventually to a counter-clockwise spiral wave (or -1 to 1 for the reversed order).

1.4 Detection of simulated spiral waves

The vertices closest to each electrode were identified, and their phases were fed to the graph algorithm, with same parameters used for our ECoG data. Let *G* be the maximum directed spanning tree of an identified TW at time *t*. We define the spiral index (SPI) as follows:

$$SPI(G) = \frac{\sum_{I=1}^{N} AD(I,R)}{0.5\pi N} \quad (3)$$

where *R* is the root node, and *N* is the number of child nodes in *G*. The angle of deviation *AD*(*I*, *R*) is calculated by the following steps (see Figure S15B):

1) Let *R* be the root node, *I* be a child node, and *J* be the immediate predecessor of *I* in *G*; *r, i, j* be vertices of the surface mesh closest to *R, I, J*, respectively
2) Find the tangent plane *PT*(*i*) of the surface mesh at vertex *i* (by MATLAB function "vertexNormal").
3) Let *r'* be the projection of *r* on *PT*(*i*), and $V_{r'i}$ be the vector from *r'* to *i*; let *j'* be the projection of *j* on *PT*(*i*), and $V_{j'i}$ be the vector from *j'* to *i*.
4) *AD*(*I*, *R*) is defined as the signed angle between $V_{j'i}$ and $V_{r'i}$.

The angle of deviation for spiral-out waves typically does not exceed 90°. When defining SPI, we rescale it by 0.5π, so that the value ranges from -1 to 1. A positive SPI indicates clockwise spirality, and a negative SPI indicates counter-clockwise spirality. A large absolute value of SPI indicates strong and consistent spirality, and a small absolute value of SPI indicates weak or random spirality.

2 Supplementary Tables

Abbreviation	Description	Marmoset_S	Marmoset_L
A1/2	Areas 1 and 2 of cortex	38 39 45	40 48 113 114
A19DI	Area 19 of cortex; dorsointermediate part	86	11 82
A3a	Area 3a of cortex (primary somatosensory)	41 43 48	37 39 51 121
A3b	Area 3b of cortex (primary somatosensory)	40 44	35 36 49 50 112
A46V	Area 46 of cortex; ventral part		60
A4ab	Area 4 of cortex; parts a and b (primary motor)	42	38 52 53 54
A6DC	Area 6 of cortex; dorsocaudal part	50 51	118 119
A6DR	Area 6 of cortex; dorsorostral part	53	64 126
A6M	Area 6 of cortex; medial (supplementary motor) part		117 128
A6Va	Area 6 of cortex; ventral; part a	49 55	124 125
A6Vb	Area 6 of cortex; ventral; part b	59	
A8C	Area 8 of cortex; caudal part	54	120
A8aD	Area 8a of cortex; dorsal part	61	56 62
A8aV	Area 8a of cortex; ventral part	60	57 58
A8b	Area 8b of cortex	52 64	55
A9	Area 9 of cortex	63	
AIP	Anterior intraparietal area of cortex		
AuA1	Auditory cortex; primary area	27 28 37	100 110
AuAL	Auditory cortex; anterolateral area		109
AuCL	Auditory cortex; caudolateral area		
AuCM	Auditory cortex; caudomedial area		
AuCPB	Auditory cortex; caudal parabelt area		102
AuML	Auditory cortex; middle lateral area		101
AuR	Auditory cortex; rostral area		41
AuRPB	Auditory cortex; rostral parabelt	36	43
AuRT	Auditory cortex; rostrotemporal part	46	42
AuRTL	Auditory cortex; rostrotemporal lateral area	35	47
FST	Fundus of superior temporal sulcus area of cortex	15 18 94	26 27 87
LIP	Lateral intrapartietal area of cortex	11	15 34 91
MIP	Medial intraparietal area of cortex	10	16 33
MST	Medial superior temporal area of cortex	19 20	28
OPt	Occipito-parietal transitional area of cortex	12	19 90
PE	Parietal area PE	23 24 25	97 98 99
PEC	Parietal area PE; caudal part		17 31 32
PF	Parietal area PF		115
PFG	Parietal area PFG	26	95 96
PG	Parietal area PG	21	29 30
ProM	Proisocortical motor region (precentral opercular cortex)	47 56 57	122 123
S2E	Secondary somatosensory cortex; external part		111 116
S2PR	Secondary somatosensory cortex; parietal rostral area		
STR	Superior temporal rostral area	32	46
TE2	Temporal area TE2		45 105 106
TE3	Temporal area TE3	16 17 30 93	23 24 25 103 104 107
TEO	Temporal area TE; occipital part	92	
TPO	Temporo-parieto-occipital association area (superior temporal polysensory cortex)	7 29	44 108
V1	Visual area 1	68 69 70 71 72 73 80 81 88 89	65 66 67 68 69 70 71 74 75 76 77 78
V2	Visual area 2	67 74 77 82 87 90	1 2 3 4 5 6 12 13 14 72 73
V3	Visual area 3 (ventrolateral posterior area)	75 85 91	8 9 10 83 84
V3A	Visual area 3A (dorsoanterior area)	84	92 94
V4	Visual area 4 (ventrolatereral anterior area)	95 96	85
V4T	Visual area 4; transitional part		20 22 88 89
V5	Visual area 5 (middle temporal area)	3 13 14	21
V6	Visual area 6 (dorsomedial area)	65 66 83	79 80 81 93
V6A	Visual area 6A (posterior parietal medial area)		18

Table S1. Abbreviations for brain areas.

The definition of brain areas follows the BMA 2019 Ex Vivo v1.0.0 atlas. The third and fourth columns show electrodes located in each area for Marmoset S and Marmoset L, respectively. The purple background indicates areas of interest to calculate the spatiotemporal scope in the visual area.

V1		Marmoset S			Marmoset L		
		Contra	Ipsi	p-value	Contra	Ipsi	p-value
Trough latency	Median	33	46	3.0×10^{-114}	37	37	0.84
	MADM	5.08	9.04	4.0×10^{-55}	5.68	8.01	5.3×10^{-24}
Peak 1 latency	Median	14	24	1.8×10^{-36}	20	17	1.8×10^{-6}
	MADM	7.20	10.13	7.2×10^{-27}	6.07	7.84	2.1×10^{-12}
Peak 2 latency	Median	55	89	2.2×10^{-142}	65	74	4.5×10^{-11}
	MADM	7.63	21.67	2.1×10^{-154}	15.62	15.75	0.79
Amplitude 1		59.96	62.53	0.016	121.32	116.17	0.056
Amplitude 2		93.37	57.98	1.6×10^{-110}	127.45	141.16	5.1×10^{-7}

V2		Marmoset S			Marmoset L		
		Contra	Ipsi	p-value	Contra	Ipsi	p-value
Trough latency	Median	39	46	3.6×10^{-131}	39	42	1.3×10^{-33}
	MADM	3.91	6.67	1.8×10^{-40}	4.20	6.64	1.9×10^{-32}
Peak 1 latency	Median	14	25	7.3×10^{-48}	18	21	3.8×10^{-9}
	MADM	8.27	9.96	1.6×10^{-10}	6.60	8.30	5.9×10^{-13}
Peak 2 latency	Median	62	92	1.1×10^{-131}	88	92	0.068
	MADM	11.38	14.56	7.6×10^{-10}	16.18	12.85	3.8×10^{-12}
Amplitude 1		102.69	106.58	0.22	153.92	153.82	0.68
Amplitude 2		110.36	92.38	1.7×10^{-36}	146.00	144.51	0.99

V3/V4		Marmoset S			Marmoset L		
		Contra	Ipsi	p-value	Contra	Ipsi	p-value
Trough latency	Median	43	47	9.9×10^{-49}	40	42	5.2×10^{-10}
	MADM	4.15	6.68	1.3×10^{-29}	4.30	7.22	3.3×10^{-40}
Peak 1 latency	Median	16	20	9.3×10^{-12}	15	20	1.0×10^{-11}
	MADM	9.14	10.30	1.4×10^{-6}	7.04	8.76	1.5×10^{-12}
Peak 2 latency	Median	80	92	9.5×10^{-46}	87	88	0.021
	MADM	13.74	10.67	3.9×10^{-12}	15.78	13.20	4.3×10^{-7}
Amplitude 1		142.91	147.85	0.082	193.60	252.60	6.0×10^{-31}
Amplitude 2		141.74	129.79	1.6×10^{-15}	204.65	219.68	9.9×10^{-8}

Table S2. Statistics on ERP features

Trough is the minimum between 20 and 60 ms. Peak 1 is the maximum between 0 ms and Trough. Peak 2 is the maximum between Trough and 120 ms. Amplitude 1 is the median difference between Peak 1 and Trough. Amplitude 2 is the median difference between Peak 2 and Trough. Medians between contraversive and ipsiversive saccades

were compared by the two-tailed Wilcoxon rank-sum test, and mean absolute deviations from the median (MADM) were compared by the Brown–Forsythe test. The gray background indicates conditions with shorter median latency, smaller MADM of latency, or larger amplitude. P-values < 0.01 were colored red.

V1		Marmoset S, Contra			Marmoset L, Contra		
		Sacc. Off	Sacc. On	p-value	Sacc. Off	Sacc. On	p-value
Trough latency	Median	33	66		37	65	
	MADM	5.08	5.74	8.3×10^{-4}	5.69	5.55	0.54
Peak 1 latency	Median	14	47		20	48	
	MADM	7.20	7.89	0.0034	6.07	5.59	0.047
Peak 2 latency	Median	55	89		65	93	
	MADM	7.63	8.08	0.31	15.62	15.41	0.71
Amplitude 1		59.96	60.90	0.36	121.32	118.50	0.55
Amplitude 2		93.37	93.93	0.52	127.45	127.34	0.87

V2		Marmoset S, Contra			Marmoset L, Contra		
		Sacc. Off	Sacc. On	p-value	Sacc. Off	Sacc. On	p-value
Trough latency	Median	39	73		39	66	
	MADM	3.91	5.09	9.8×10^{-12}	4.20	3.51	8.8×10^{-5}
Peak 1 latency	Median	14	46		18	47	
	MAD	8.27	9.49	3.6×10^{-8}	6.60	5.90	8.1×10^{-4}
Peak 2 latency	Median	62	97		88	118	
	MADM	11.38	11.31	0.89	16.18	15.97	0.64
Amplitude 1		102.69	105.13	0.093	153.92	152.84	0.65
Amplitude 2		110.36	111.26	0.45	145.99	146.09	0.99

V3/V4		Marmoset S, Contra			Marmoset L, Contra		
		Sacc. Off	Sacc. On	p-value	Sacc. Off	Sacc. On	p-value
Trough latency	Median	43	77		40	68	
	MADM	4.15	5.00	1.0×10^{-5}	4.30	3.48	6.5×10^{-6}
Peak 1 latency	Median	16	46		15	45	
	MADM	9.14	10.22	5.1×10^{-6}	7.04	6.68	0.091
Peak 2 latency	Median	80	113		87	117	
	MADM	13.74	12.86	0.039	15.78	16.27	0.28
Amplitude 1		142.91	144.53	0.15	193.60	189.78	0.30
Amplitude 2		141.74	141.49	0.98	204.65	204.23	0.76

Table S3. Comparison of ERP features between 2 data alignments

Only contraversive data are shown. Data aligned on saccade offset (Sacc. Off) is the same as Table S2. For ERP aligned on saccade onset (Sacc. On), different time windows were used: Trough is the minimum between 50 and 90 ms, Peak 1 is the maximum between 30 ms and Trough, and Peak 2 is the maximum between Trough

and 150 ms. Amplitude 1 is the median difference between Peak 1 and Trough. Amplitude 2 is the median difference between Peak 2 and Trough. Medians between contraversive and ipsiversive saccades were compared by the two-tailed Wilcoxon rank-sum test, and mean absolute deviations from the median (MADM) were compared by the Brown–Forsythe test. The gray background indicates conditions with a smaller MADM of latency. P-values < 0.01 were colored red.

Marmoset S

Duration	0°	45°	90°	135°	180°	225°	270°	315°
-10 to 40 median	25	23	22	32	41	43	38	27.5
50 to 100 median	17	17	17	19	18	18	20	18
p-value	2.08E-25	1.23E-14	1.58E-09	9.09E-44	1.07E-128	2.07E-147	3.64E-63	3.69E-26
**Coverage**	**0°**	**45°**	**90°**	**135°**	**180°**	**225°**	**270°**	**315°**
-10 to 40 median	29	32	31	44	54	51	50	35
50 to 100 median	25	23	23	27	29	28	33	29
p-value	1.26E-10	6.58E-17	4.51E-14	8.94E-48	2.13E-111	5.21E-101	1.34E-39	1.06E-08
**Amplitude**	**0°**	**45°**	**90°**	**135°**	**180°**	**225°**	**270°**	**315°**
-10 to 40 median	13.33294	13.11092	13.73837	14.54694	21.9735	21.94766	16.06463	13.87335
50 to 100 median	9.934719	10.29737	10.89576	11.56237	14.16779	14.89204	12.25304	10.2421
p-value	8.28E-49	5.56E-39	1.00E-33	2.27E-35	2.31E-109	3.69E-95	5.62E-38	7.86E-51
**Frequency**	**0°**	**45°**	**90°**	**135°**	**180°**	**225°**	**270°**	**315°**
-10 to 40 median	30.10508	30.65877	30.50952	29.9357	29.32768	28.67333	29.14479	29.52182
50 to 100 median	30.35354	31.54304	31.18429	30.73058	29.7366	29.08819	29.5095	30.14134
p-value	0.031106	9.70E-10	1.08E-07	7.50E-08	0.027488	0.004547	0.007599	2.81E-06
**Velocity**	**0°**	**45°**	**90°**	**135°**	**180°**	**225°**	**270°**	**315°**
-10 to 40 median	1556.536	1654.547	1499.704	1367.691	1267.374	1273.5	1362.081	1526.031
50 to 100 median	1589.089	1616.705	1572.83	1516.144	1526.537	1500.722	1505.235	1486.642
p-value	0.610775	0.006745	0.045435	4.47E-10	2.39E-38	8.71E-40	9.58E-08	0.725524

Marmoset L

Duration	0°	45°	90°	135°	180°	225°	270°	315°
-10 to 40 median	32	31	30	25	25	24	31	29
50 to 100 median	21	22	20	19	20	21	22	21
p-value	2.53E-37	1.83E-20	6.81E-28	4.43E-11	4.33E-10	0.010249	1.91E-23	1.24E-22
**Coverage**	**0°**	**45°**	**90°**	**135°**	**180°**	**225°**	**270°**	**315°**
-10 to 40 median	57	55	53	51	49	52	64	69
50 to 100 median	46	43	41	42	42	46	46.5	48
p-value	1.18E-11	1.13E-09	1.01E-15	5.10E-10	1.62E-05	0.001225	1.08E-23	1.22E-28
**Amplitude**	**0°**	**45°**	**90°**	**135°**	**180°**	**225°**	**270°**	**315°**
-10 to 40 median	29.15256	26.81114	26.15141	25.79342	23.70747	23.19347	25.20506	26.53977
50 to 100 median	18.72541	18.9791	18.18317	17.52101	16.58766	16.52538	17.29695	18.26649
p-value	1.63E-75	3.83E-45	1.98E-52	1.98E-57	2.55E-54	9.77E-56	9.94E-64	9.61E-68
**Frequency**	**0°**	**45°**	**90°**	**135°**	**180°**	**225°**	**270°**	**315°**
-10 to 40 median	29.53447	29.5896	29.36431	29.10268	28.81649	28.61387	28.86094	29.3103
50 to 100 median	29.85777	30.31649	29.85162	29.95556	30.02509	29.90409	29.76757	29.8897
p-value	0.010861	9.47E-06	0.004728	1.66E-06	1.49E-12	7.34E-18	1.06E-10	0.003052
**Velocity**	**0°**	**45°**	**90°**	**135°**	**180°**	**225°**	**270°**	**315°**
-10 to 40 median	1608.329	1615.538	1587.903	1551.603	1558.955	1516.75	1397.655	1483.927
50 to 100 median	1723.042	1747.206	1693.166	1676.984	1683.476	1684.628	1677.987	1688.034
p-value	0.000104	3.51E-07	0.002613	8.23E-05	5.24E-06	9.11E-09	3.67E-27	9.79E-18

Table S4. Comparison of wave parameters between the -10 to 40 ms and 50 to 100 ms TWs

Statistics were done with the two-sided Wilcoxon rank sum test.

	Electrode count	All combinations	Neighbors only	Immediate neighbors only
Marmoset S	82	3321	537 (16.2%)	269 (8.1%)
Marmoset L	122	7381	1134 (15.4%)	473 (6.4%)

Table S5. Number of edges during neighbor graph construction

"Electrode count" includes only valid electrodes. "All combinations" counts all possible pairs of valid electrodes (i.e., N(N-1)/2). "Neighbors only" includes only pairs whose geodesic distance is less than 6 mm. "Immediate neighbors only" further excludes geometrically redundant edges (see Material and Methods 5.9.1) on the basis of "Neighbors only". Percentages in the parentheses are relative to "All combinations". The algorithm successfully reduced the number of edges to compute by over 90%.

3 Supplementary Figures

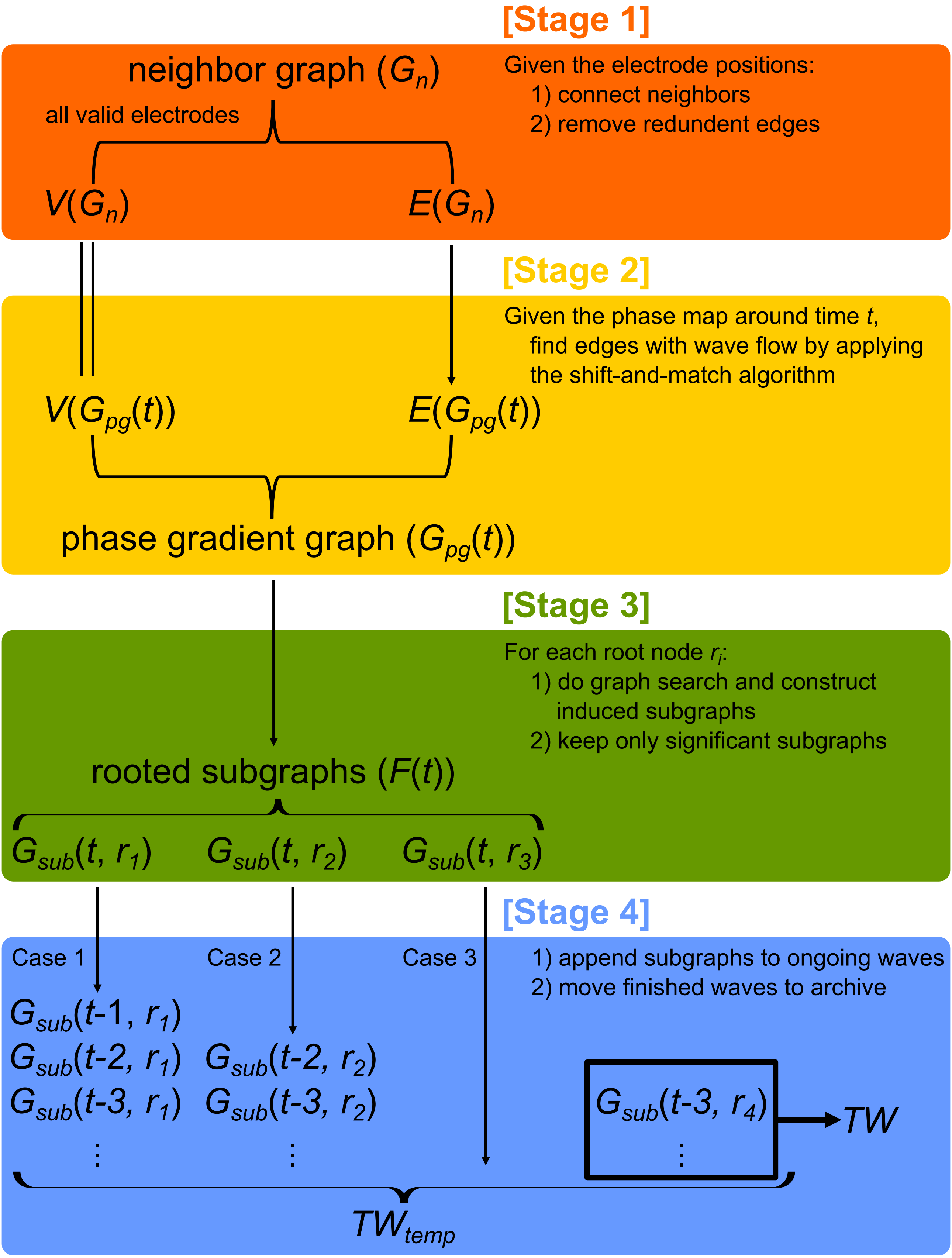
[Stage 1]
neighbor graph (G_n)
all valid electrodes
V(G_n)
E(G_n)
Given the electrode positions:
1) connect neighbors
2) remove redundent edges
[Stage 2]
Given the phase map around time t,
find edges with wave flow by applying
the shift-and-match algorithm
V(G_pg(t))
E(G_pg(t))
phase gradient graph (G_pg(t))
[Stage 3]
For each root node r_i:
1) do graph search and construct
induced subgraphs
2) keep only significant subgraphs
rooted subgraphs (F(t))
G_sub(t, r_1)
G_sub(t, r_2)
G_sub(t, r_3)
[Stage 4]
Case 1
Case 2
Case 3
1) append subgraphs to ongoing waves
2) move finished waves to archive
G_sub(t-1, r_1)
G_sub(t-2, r_1)
G_sub(t-3, r_1)
G_sub(t-2, r_2)
G_sub(t-3, r_2)
G_sub(t-3, r_4)
TW
TW_temp

Figure S1. Flow diagram of the graph-based algorithm

The diagram summarizes functionalities of each of the 4 stages and their relationships. See Material and Methods 5.9 and Supplementary Material 1.1 for details.

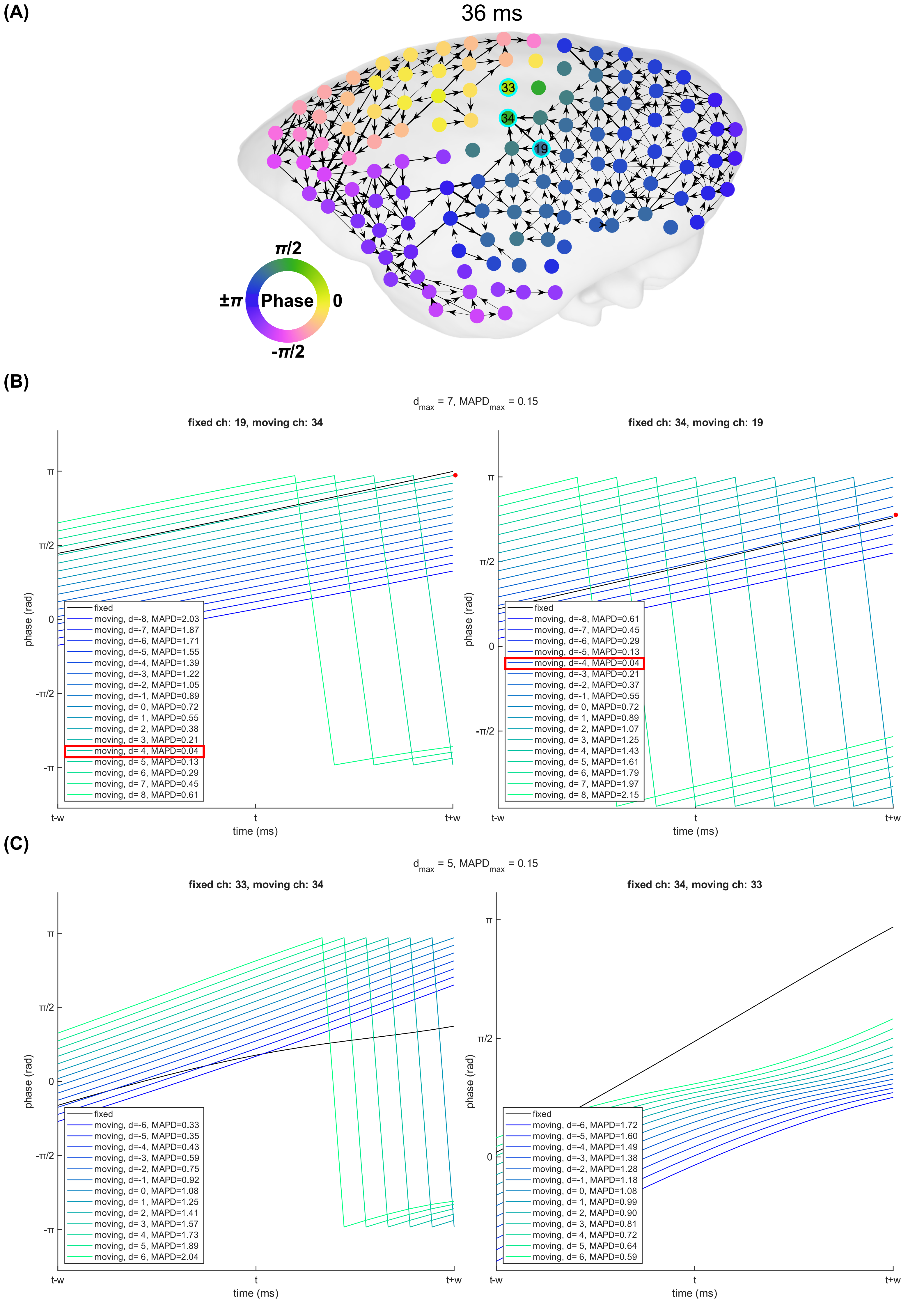

(A)
36 ms
33
34
19
π/2
±π
Phase
0
-π/2
(B)
d_max = 7, MAPD_max = 0.15
fixed ch: 19, moving ch: 34
fixed ch: 34, moving ch: 19
π
π/2
0
-π/2
-π
phase (rad)
t-w
t
t+w
time (ms)
fixed
moving, d=-8, MAPD=2.03
moving, d=-7, MAPD=1.87
moving, d=-6, MAPD=1.71
moving, d=-5, MAPD=1.55
moving, d=-4, MAPD=1.39
moving, d=-3, MAPD=1.22
moving, d=-2, MAPD=1.05
moving, d=-1, MAPD=0.89
moving, d= 0, MAPD=0.72
moving, d= 1, MAPD=0.55
moving, d= 2, MAPD=0.38
moving, d= 3, MAPD=0.21
moving, d= 4, MAPD=0.04
moving, d= 5, MAPD=0.13
moving, d= 6, MAPD=0.29
moving, d= 7, MAPD=0.45
moving, d= 8, MAPD=0.61
fixed
moving, d=-8, MAPD=0.61
moving, d=-7, MAPD=0.45
moving, d=-6, MAPD=0.29
moving, d=-5, MAPD=0.13
moving, d=-4, MAPD=0.04
moving, d=-3, MAPD=0.21
moving, d=-2, MAPD=0.37
moving, d=-1, MAPD=0.55
moving, d= 0, MAPD=0.72
moving, d= 1, MAPD=0.89
moving, d= 2, MAPD=1.07
moving, d= 3, MAPD=1.25
moving, d= 4, MAPD=1.43
moving, d= 5, MAPD=1.61
moving, d= 6, MAPD=1.79
moving, d= 7, MAPD=1.97
moving, d= 8, MAPD=2.15
(C)
d_max = 5, MAPD_max = 0.15
fixed ch: 33, moving ch: 34
fixed ch: 34, moving ch: 33
fixed
moving, d=-6, MAPD=0.33
moving, d=-5, MAPD=0.35
moving, d=-4, MAPD=0.43
moving, d=-3, MAPD=0.59
moving, d=-2, MAPD=0.75
moving, d=-1, MAPD=0.92
moving, d= 0, MAPD=1.08
moving, d= 1, MAPD=1.25
moving, d= 2, MAPD=1.41
moving, d= 3, MAPD=1.57
moving, d= 4, MAPD=1.73
moving, d= 5, MAPD=1.89
moving, d= 6, MAPD=2.04
fixed
moving, d=-6, MAPD=1.72
moving, d=-5, MAPD=1.60
moving, d=-4, MAPD=1.49
moving, d=-3, MAPD=1.38
moving, d=-2, MAPD=1.28
moving, d=-1, MAPD=1.18
moving, d= 0, MAPD=1.08
moving, d= 1, MAPD=0.99
moving, d= 2, MAPD=0.90
moving, d= 3, MAPD=0.81
moving, d= 4, MAPD=0.72
moving, d= 5, MAPD=0.64
moving, d= 6, MAPD=0.59

Figure S2. The shift-and-match algorithm for determining edges with wave flow

(A) Same as Figure 1B. Data from three electrodes (electrode 19, 34, and 33, highlighted with cyan circles) are shown in the following panels as examples.

(B) Segments of instantaneous phase time series from electrode 19 (black) and 34 (shifting from green to blue, with increasing delay value d). Search range d_{max} and threshold of mean absolute phase difference ($MAPD_{max}$) are shown on the top. In this case, with symmetrical delays (red boxes and dots; 19 fixed, 34 moving: 4 ms; 34 fixed, 19 moving: -4 ms) the minimal MAPDs (19 fixed, 34 moving: 0.04 rad; 34 fixed, 19 moving: 0.04 rad) are both below the threshold. Therefore, we consider a wave flow from electrode 19 to electrode 34. Note that MAPD is calculated using the analytic signals (see Equation 9-1), so the sharp numerical transition of phase at $\pm\pi$ does not influence the result.

(C) Similar to (B) but for electrode 33 and electrode 34. In this case, MAPDs are all above the threshold over the entire search range of d. Therefore, we consider no wave flow between electrode 33 and electrode 34. Note that due to differences in frequency component (slope in this figure), searching beyond d_{max} does not change the decision, demonstrating the robustness of the algorithm.

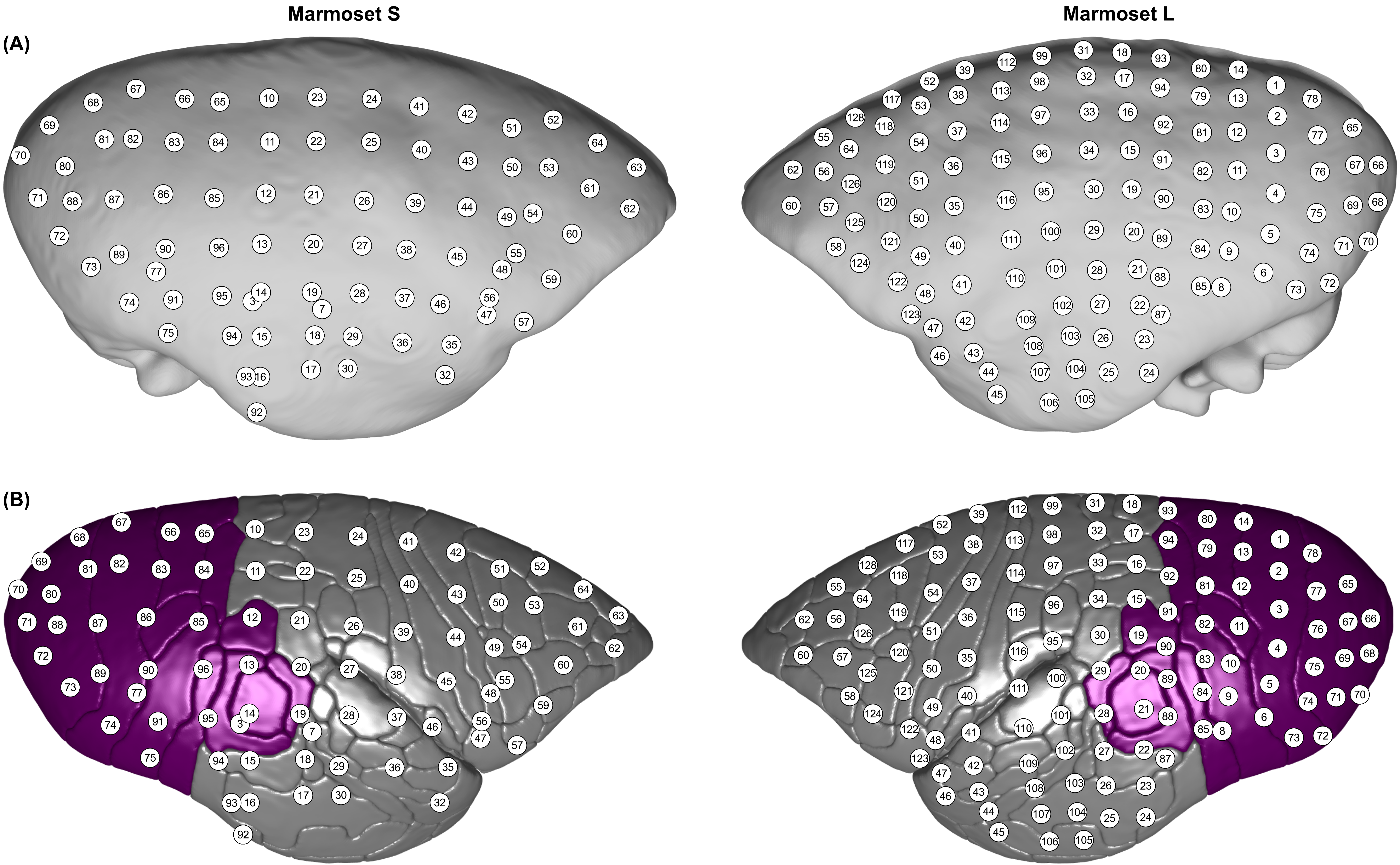
Marmoset S
Marmoset L
(A)
(B)

Figure S3. Electrode location of the two marmosets

Labels on the electrode indicate electrode ID (see Table S1 for covered brain area). Areas colored in purple are visual areas used to calculate the spatiotemporal scope.

(A) Electrodes of the ECoG array were plotted on 3-D brain models of Marmoset S (left) and Marmoset L (right).

(B) Registered electrode locations on the BMA 2019 Ex Vivo v1.0.0 atlas (see Material and Methods) for Marmoset S (left) and Marmoset L (right).

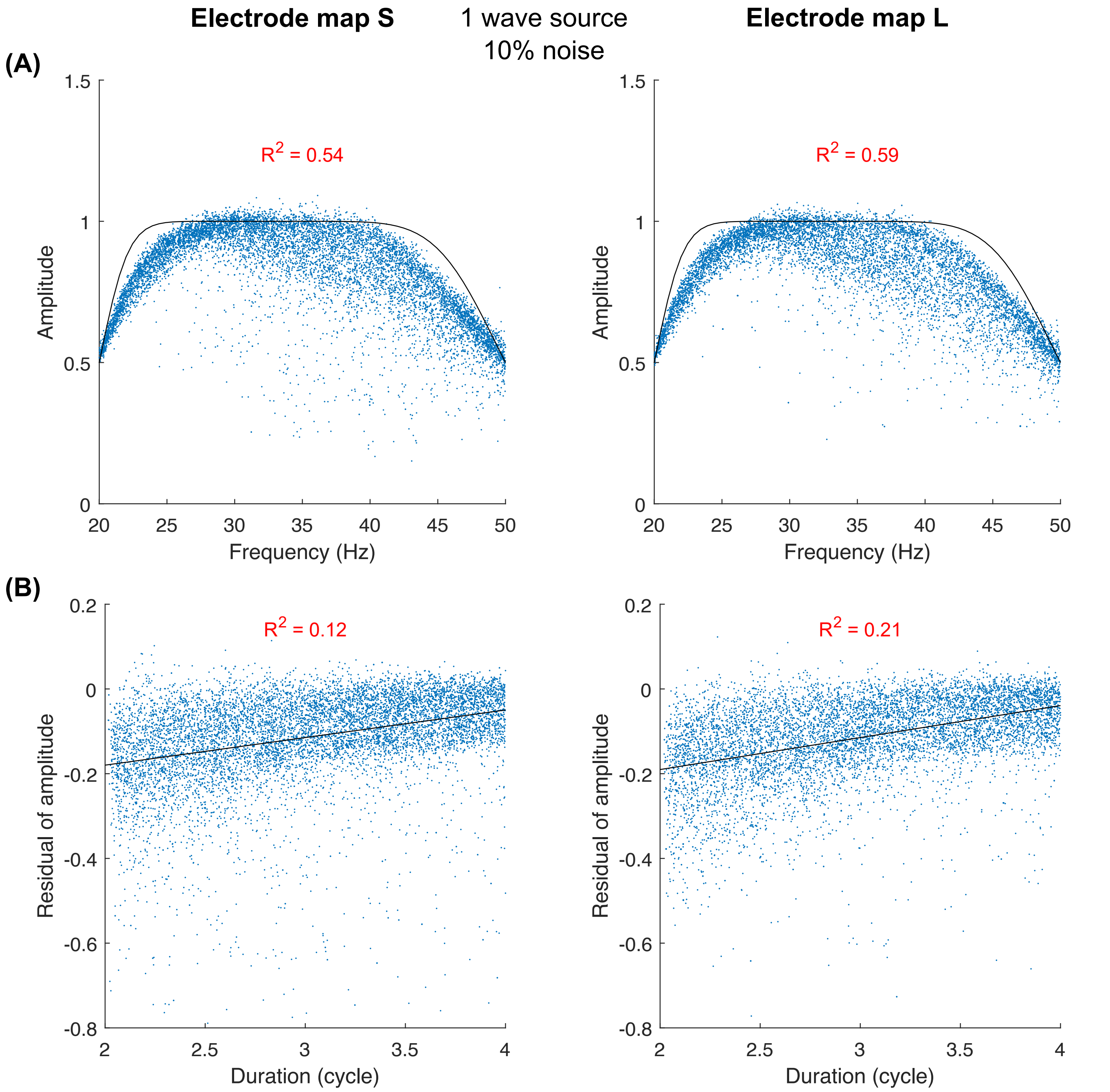

Electrode map S
1 wave source
10% noise
Electrode map L
(A)
R^2 = 0.54
R^2 = 0.59
Amplitude
Frequency (Hz)
(B)
R^2 = 0.12
R^2 = 0.21
Residual of amplitude
Duration (cycle)

Figure S4. Factors affecting the estimated amplitude

Each figure shows the 10,000 simulated waves in the one source, 10% noise condition.

(A) Dependence of amplitude on frequency. The black curve is the frequency response of the bandpass filter used.

(B) After subtracting the frequency response curve of the filter, the residual amplitude linearly depends on wave duration in number of cycles.

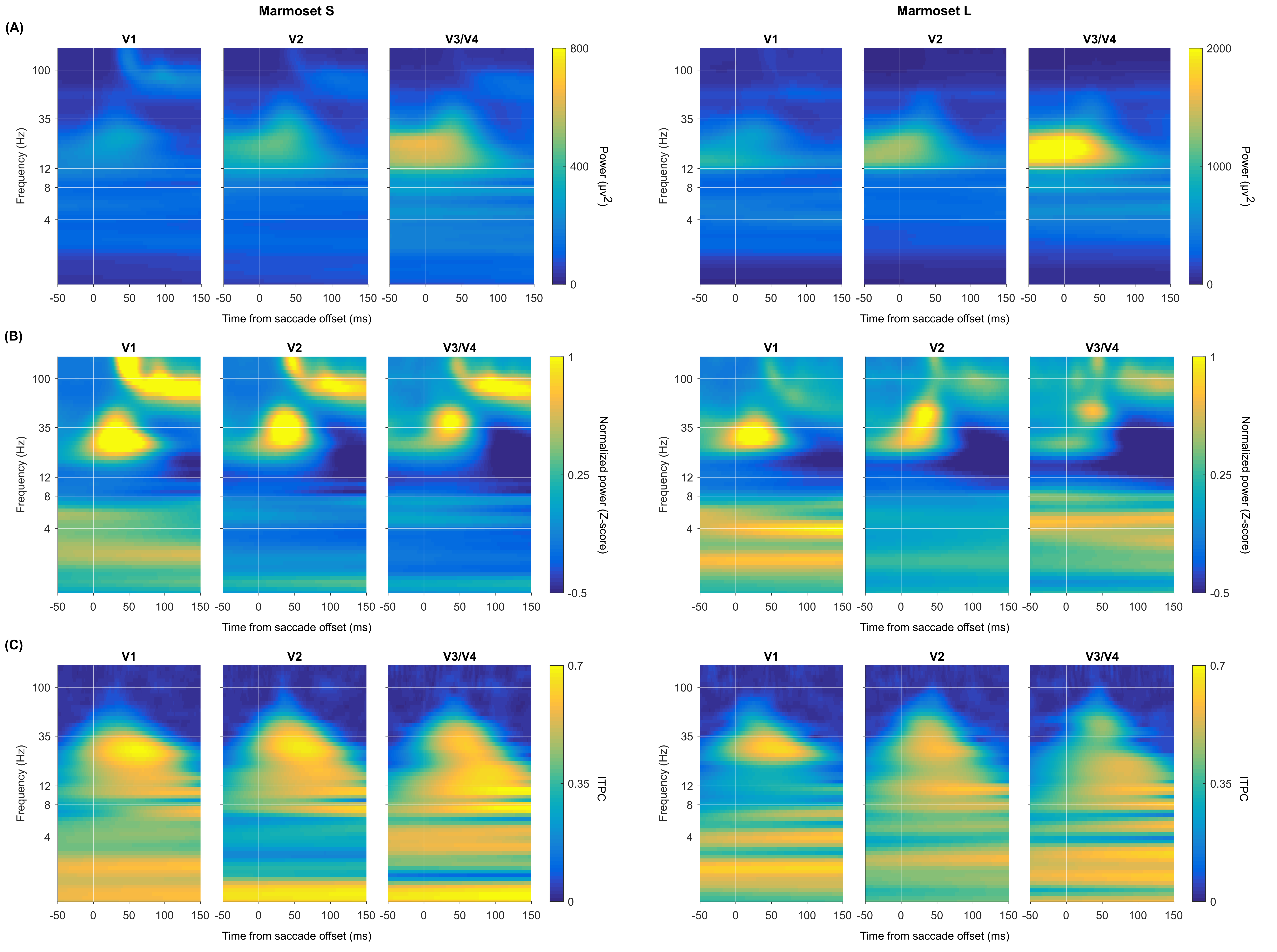

Marmoset S
Marmoset L
(A)
(B)
(C)
V1
V2
V3/V4
Frequency (Hz)
Time from saccade offset (ms)
Power (µv^2)
Normalized power (Z-score)
ITPC

Figure S5. Additional results of the time-frequency analysis

Data criteria are the same as in Figure 4.

(A) Trial-averaged raw power. Color bar labels in the parenthesis are for Marmoset L.

(B) Trial-averaged Z-scored power.

(C) Inter-trial phase clustering index (ITPC).

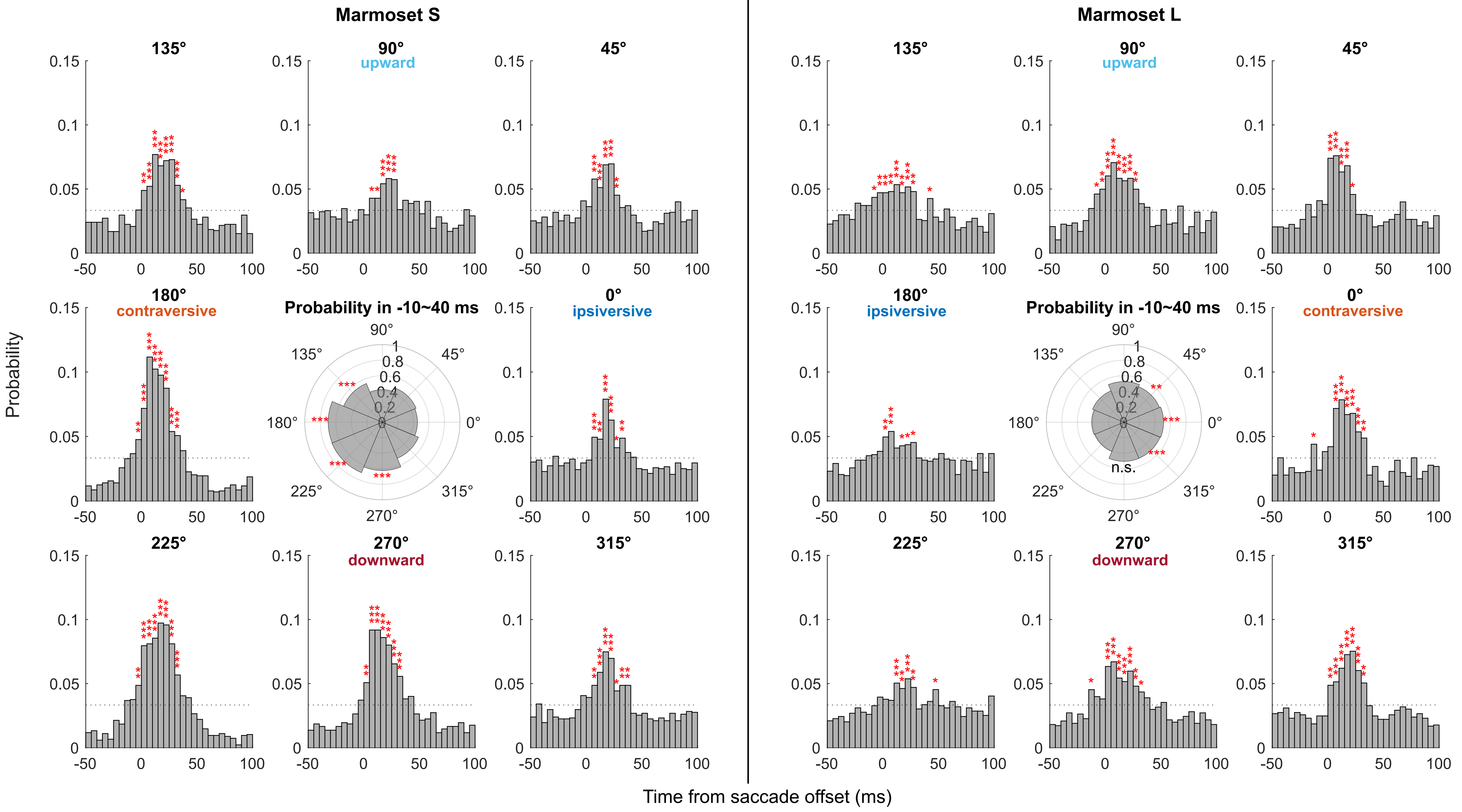
Marmoset S
Marmoset L
135°
90°
upward
45°
180°
contraversive
ipsiversive
Probability in -10~40 ms
0°
225°
270°
downward
315°
n.s.
Probability
Time from saccade offset (ms)

Figure S6. Latency of the largest TW around the saccade offset for all saccadic directions

Each surrounding panel shows the probability histogram of wave latency for one of the eight saccade directions. Only the largest wave over the visual area (see Figure S3 and Table S1) originating from V1 around saccade offset (-50 to 100 ms) in each trial was included (sample size; Marmoset S, 0°: 1355, 45°: 1350, 90°: 1238, 135°: 1248, 180°: 1281, 225°: 1357, 270°: 1024, 315°: 1377; Marmoset L, 0°: 1045, 45°: 1027, 90°: 1063, 135°: 1104, 180°: 1168, 225°: 1190, 270°: 1104, 315°: 1129). Data were partitioned into 30 bins, each 5 ms wide. Red asterisks indicate the one-tailed P-value under the binomial distribution against a chance level of 1/30 (*: $p < 0.05$, **: $p < 0.01$, ***: $p < 0.001$). The central panels show the probability for the largest waves to occur during -10 to 40 ms surrounding saccade offset for the eight saccade directions. Probability between direction pairs were compared (0° vs. 180°, 90° vs. 270°, 45° vs. 135°, 315° vs. 225°) by the Pearson's chi-squared test (*: $p < 0.05$, **: $p < 0.01$, ***: $p < 0.001$).

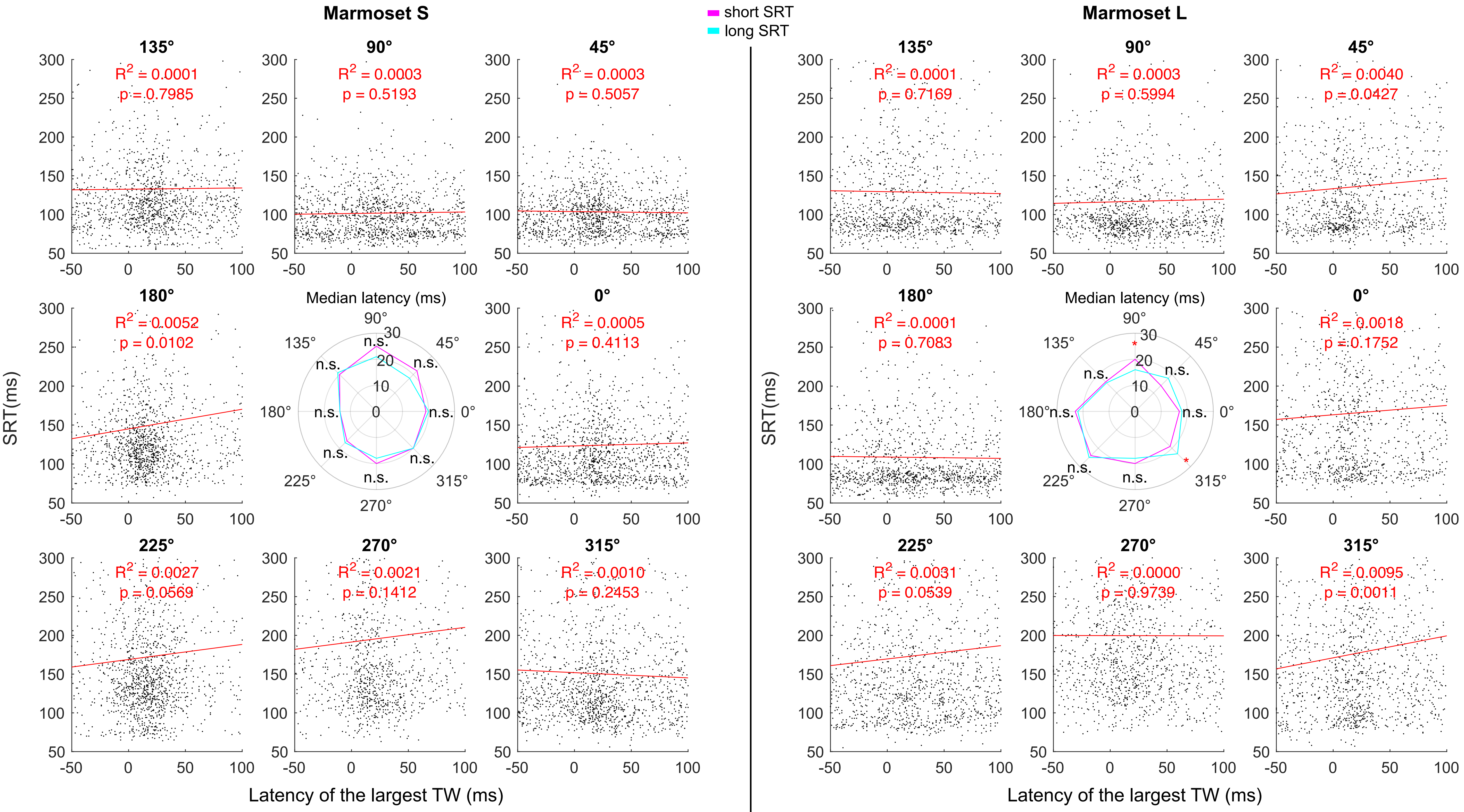

Marmoset S
Marmoset L
short SRT
long SRT
135°
R^2 = 0.0001
p = 0.7985
90°
R^2 = 0.0003
p = 0.5193
45°
R^2 = 0.0003
p = 0.5057
180°
R^2 = 0.0052
p = 0.0102
Median latency (ms)
0°
R^2 = 0.0005
p = 0.4113
225°
R^2 = 0.0027
p = 0.0569
270°
R^2 = 0.0021
p = 0.1412
315°
R^2 = 0.0010
p = 0.2453
135°
R^2 = 0.0001
p = 0.7169
90°
R^2 = 0.0003
p = 0.5994
45°
R^2 = 0.0040
p = 0.0427
180°
R^2 = 0.0001
p = 0.7083
Median latency (ms)
0°
R^2 = 0.0018
p = 0.1752
225°
R^2 = 0.0031
p = 0.0539
270°
R^2 = 0.0000
p = 0.9739
315°
R^2 = 0.0095
p = 0.0011
n.s.
SRT(ms)
Latency of the largest TW (ms)

Figure S7. Saccade reaction time (SRT) does not affect wave latency

Each surrounding panel shows the scatter plot of SRT against wave latency, and their linear fit (red line), for each of the eight saccade directions. The central panels compare the median latencies (in ms) between short SRT and long SRT trials (median split, short SRT: magenta, long SRT: cyan).

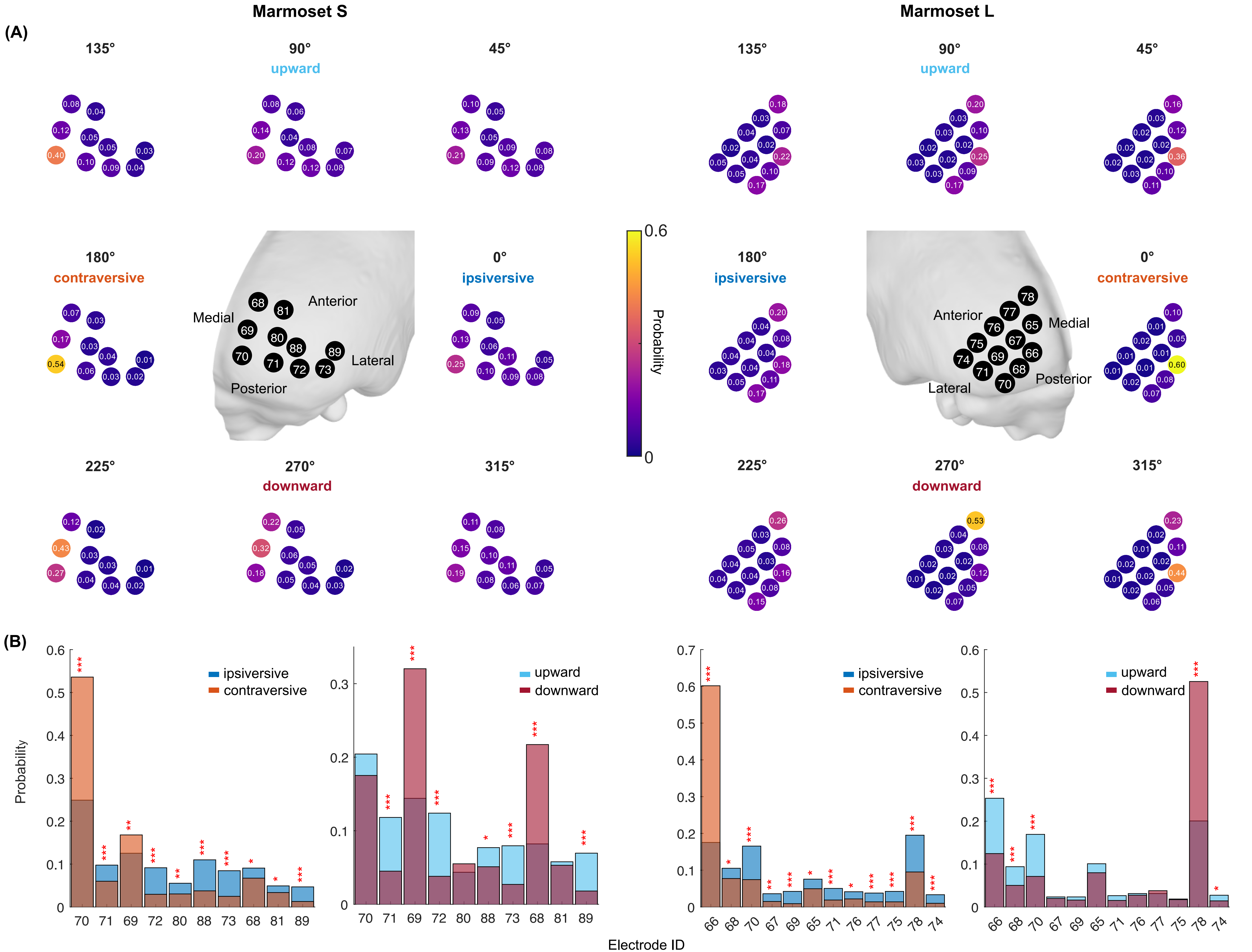

Marmoset S
Marmoset L
(A)
135°
90°
upward
45°
180°
contraversive
0°
ipsiversive
Medial
Anterior
Lateral
Posterior
225°
270°
downward
315°
Probability
(B)
ipsiversive
contraversive
upward
downward
Probability
Electrode ID

Figure S8. Distribution of wave source on V1 for all saccadic directions

Only trials with at least one identified TW occurring during -10 to 40 ms surrounding saccade offset were included (sample size; Marmoset S, 0°: 1301, 45°: 1290, 90°: 1194, 135°: 1209, 180°: 1250, 225°: 1329, 270°: 999, 315°: 1324; Marmoset L, 0°: 1009, 45°: 978, 90°: 1022, 135°: 1059, 180°: 1112, 225°: 1132, 270°: 1052, 315°: 1096)

(A) The central panels show the locations of the V1 electrodes on the brains. The surrounding panels show the probability for a TW to originate from each V1 electrode. Note that the probabilities do not sum up to 1, because the source of the largest wave can be outside of V1.

(B) Same data as (A), but comparing ipsiversive vs. contraversive saccades, and upward vs. downward saccades. Colors indicate saccade direction. Statistics was done using the Pearson's chi-squared test (*: $p < 0.05$, **: $p < 0.01$, ***: $p < 0.001$).

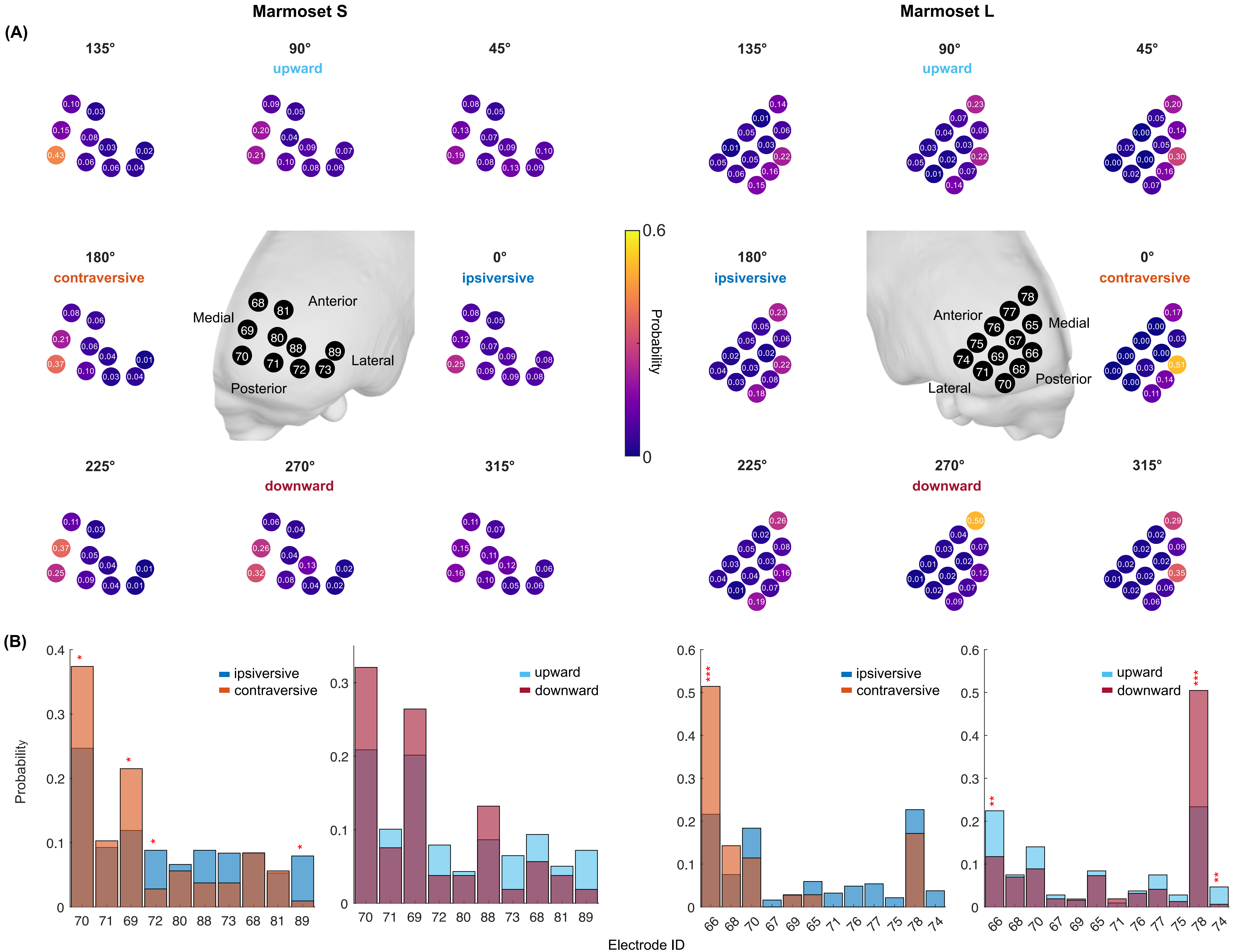

Marmoset S
Marmoset L
(A)
135°
90°
upward
45°
180°
contraversive
0°
ipsiversive
225°
270°
downward
315°
Anterior
Medial
Lateral
Posterior
Probability
(B)
ipsiversive
contraversive
upward
downward
Probability
Electrode ID

Figure S9. Distribution of wave source on V1 for overshooting saccades

Same as Figure S8, but only overshooting saccades were included (sample size; Marmoset S, 0°: 227, 45°: 356, 90°: 278, 135°: 211, 180°: 107, 225°: 271, 270°: 53, 315°: 360; Marmoset L, 0°: 35, 45°: 44, 90°: 107, 135°: 110, 180°: 185, 225°: 366, 270°: 315, 315°: 339).

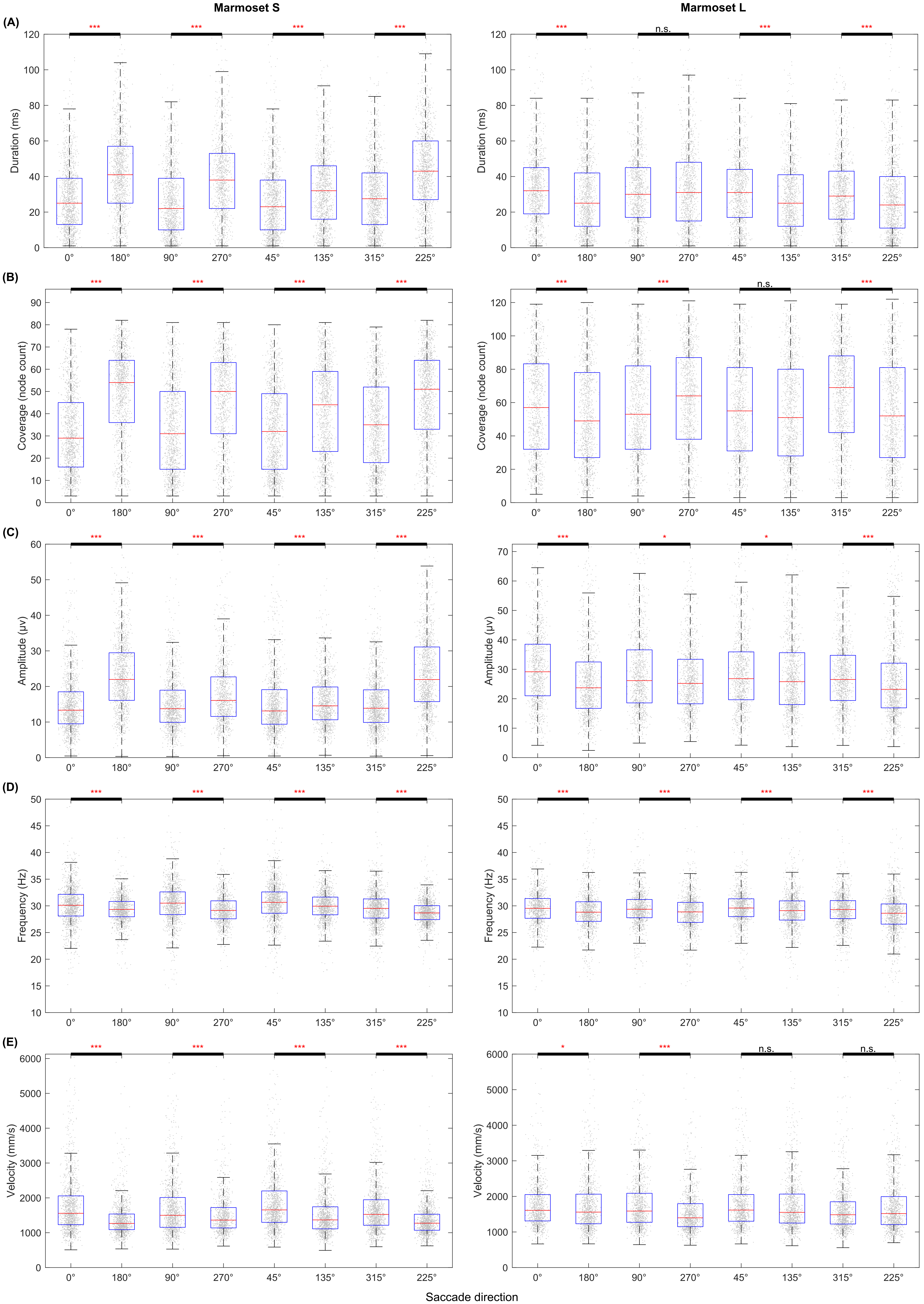

Marmoset S
Marmoset L
(A)
(B)
(C)
(D)
(E)
Duration (ms)
Coverage (node count)
Amplitude (μv)
Frequency (Hz)
Velocity (mm/s)
n.s.
***
*
0° 180° 90° 270° 45° 135° 315° 225°
Saccade direction

Figure S10. Pair-wise comparison of TW parameters

Gray dots indicate data from individual trials. In (A) and (B), dots were vertically jittered for up to 0.5 units for visibility. Box plots show the 25th, 50th, and 75th percentile of the data. The proportion of data outside the plotted range was less than 1% for each column. Whiskers indicate median ± 1.5 x inter-quartile interval or extrema, whichever is closer to the median. Statistics were done using the two-sided Wilcoxon rank sum test (*: $p < 0.05$, **: $p < 0.01$, ***: $p < 0.001$).

(A) Wave duration.

(B) Temporal maximum of covered electrodes.

(C) Temporal median of root node amplitude.

(D) Spatiotemporal median of node frequency.

(E) Spatiotemporal median of wave velocity.

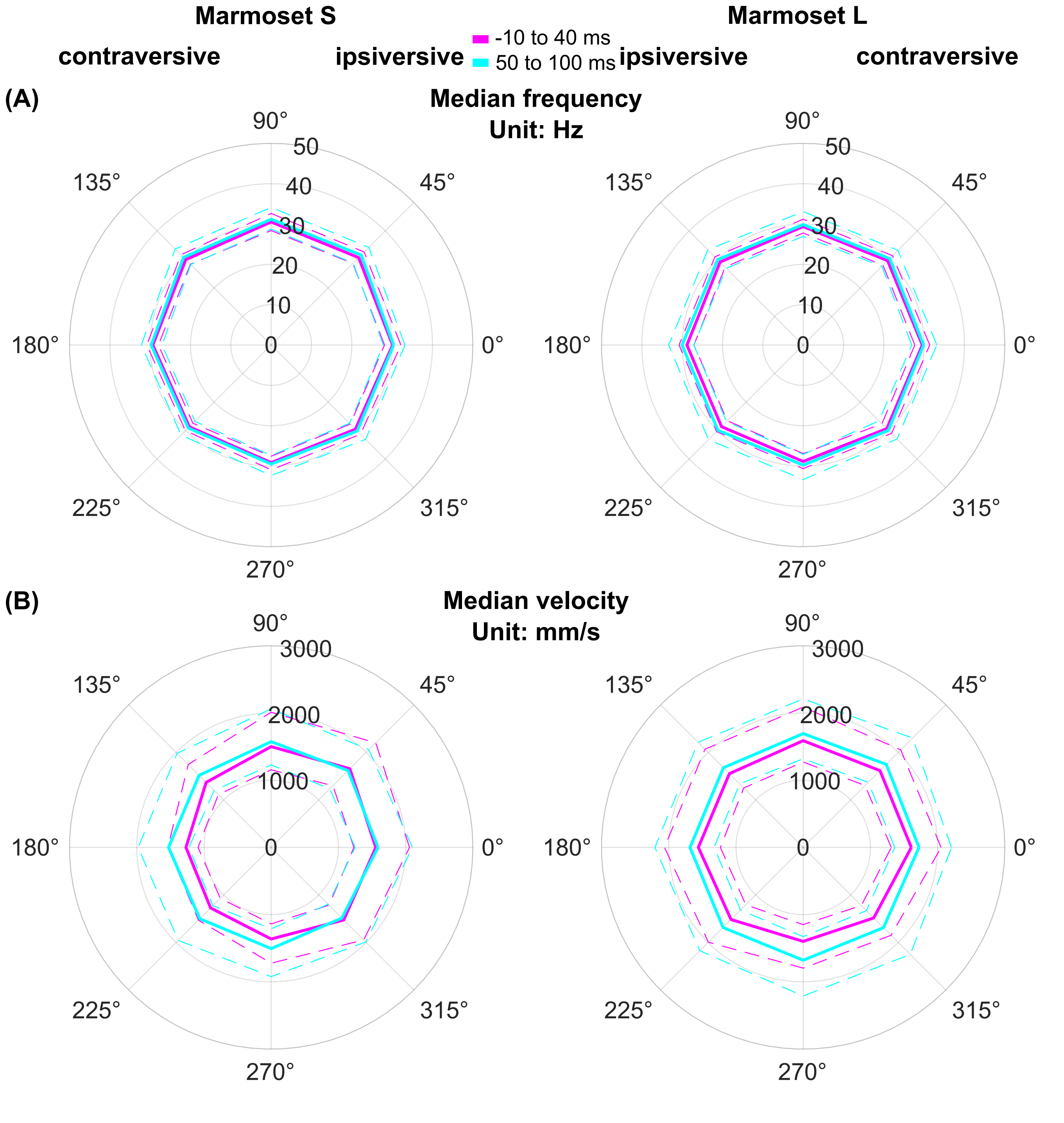

Marmoset S
Marmoset L
-10 to 40 ms
50 to 100 ms
contraversive
ipsiversive
ipsiversive
contraversive
(A)
Median frequency
Unit: Hz
90°
45°
0°
315°
270°
225°
180°
135°
0
10
20
30
40
50
(B)
Median velocity
Unit: mm/s
0
1000
2000
3000

Figure S11. Frequency and velocity of the post-saccadic TW

Similar to Figure 9, but for (A) Spatiotemporal median of node frequency (B) Spatiotemporal median of wave velocity.

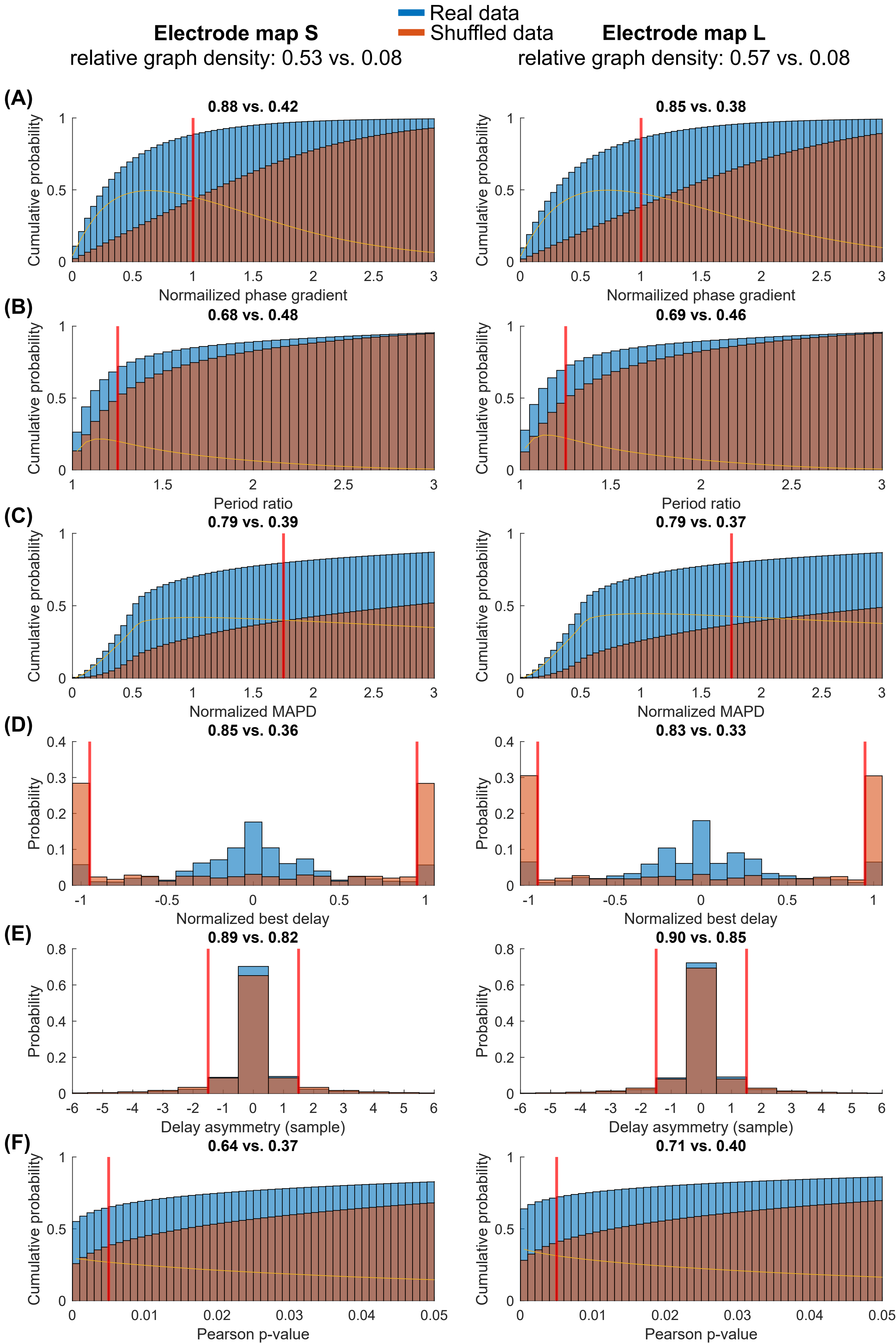

Real data
Shuffled data
Electrode map S
relative graph density: 0.53 vs. 0.08
Electrode map L
relative graph density: 0.57 vs. 0.08
(A)
0.88 vs. 0.42
0.85 vs. 0.38
Cumulative probability
Normailized phase gradient
(B)
0.68 vs. 0.48
0.69 vs. 0.46
Period ratio
(C)
0.79 vs. 0.39
0.79 vs. 0.37
Normalized MAPD
(D)
0.85 vs. 0.36
0.83 vs. 0.33
Probability
Normalized best delay
(E)
0.89 vs. 0.82
0.90 vs. 0.85
Delay asymmetry (sample)
(F)
0.64 vs. 0.37
0.71 vs. 0.40
Pearson p-value

Figure S12. Selection of fundamental parameters for the algorithm

Each panel shows the resulting relative graph density, defined as the number of edges with flow divided by the number of edges in the neighbor graph. The vertical red lines indicate the threshold values used. The numbers on top of each panel and the figure indicate the resulting relative graph density (real vs. shuffled) after applying every single threshold and all the thresholds, respectively. In panels showing cumulative probability, the yellow curves indicate the difference between real and shuffled data.

(A) Phase gradient of edges was normalized by PG_{max}.

(B) Period ratio of neighboring nodes: $max(pi_a(t), pi_b(t))$ / $min(pi_a(t), pi_b(t))$.

(C) Normalized MAPD is defined as the minimal MAPD of each edge divided by the corresponding theoretical maximum.

(D) Normalized best delay defined as the delay that gives the minimal MAPD divided by $d_{max} + 1$.

(E) Delay asymmetry calculated as $d_{ba} + d_{ab}$.

(F) P-value of the Pearson correlation between the cumulative distance and phase. Data in this panel are based on thresholds in (A)-(F).

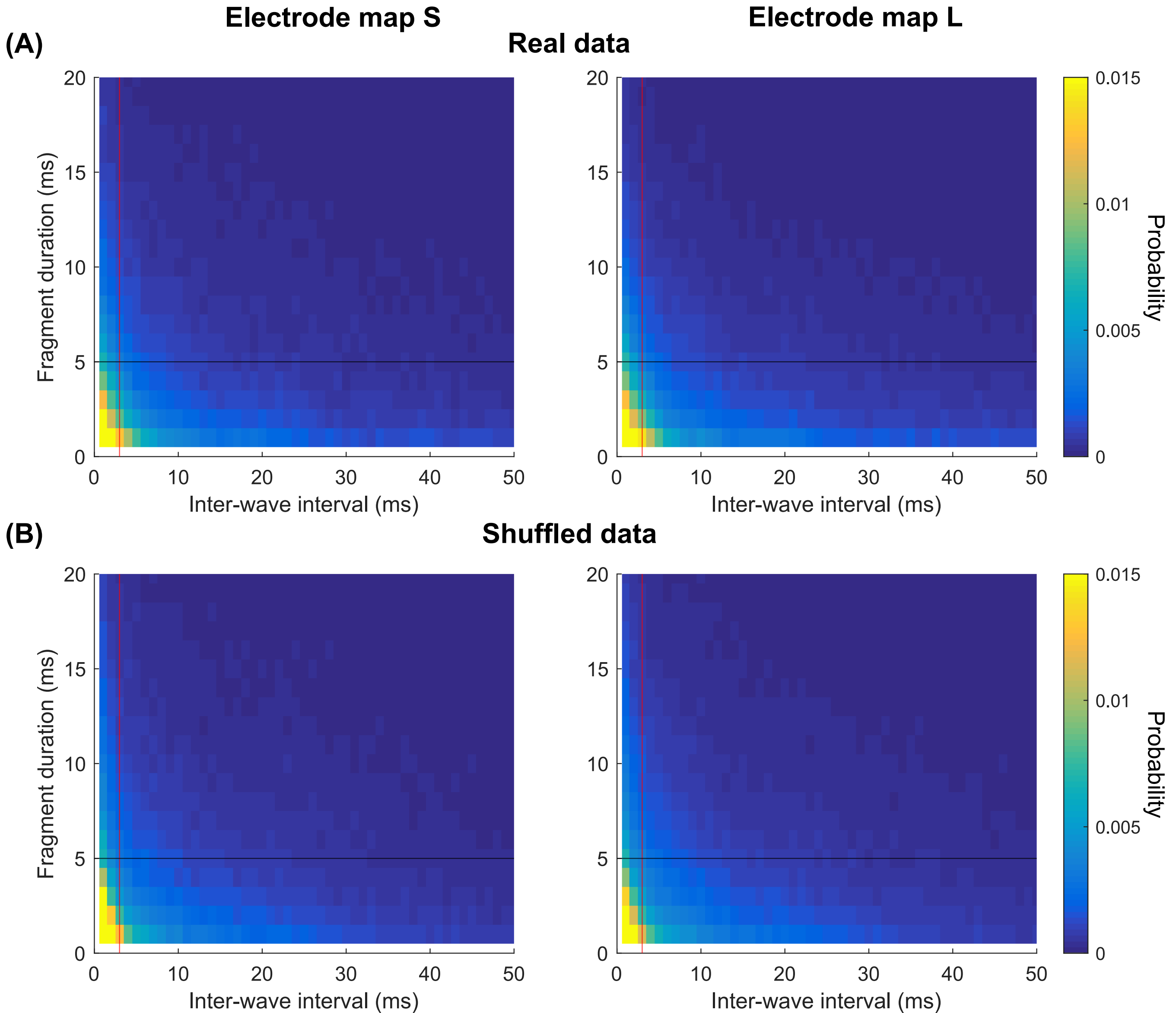

Electrode map S
Electrode map L
(A)
Real data
(B)
Shuffled data
Fragment duration (ms)
Inter-wave interval (ms)
Probability
0
5
10
15
20
30
40
50
0.005
0.01
0.015

Figure S13. Determine the maximal allowed interruption period

Color-coded 2D probability histogram showing the duration of identified wave fragment and inter-wave interval, defined as the time period from the preceding or succeeding wave fragment, whichever is shorter. The vertical red line indicates the interruption threshold used (3 ms).

(A) Real data.

(B) Shuffled data.

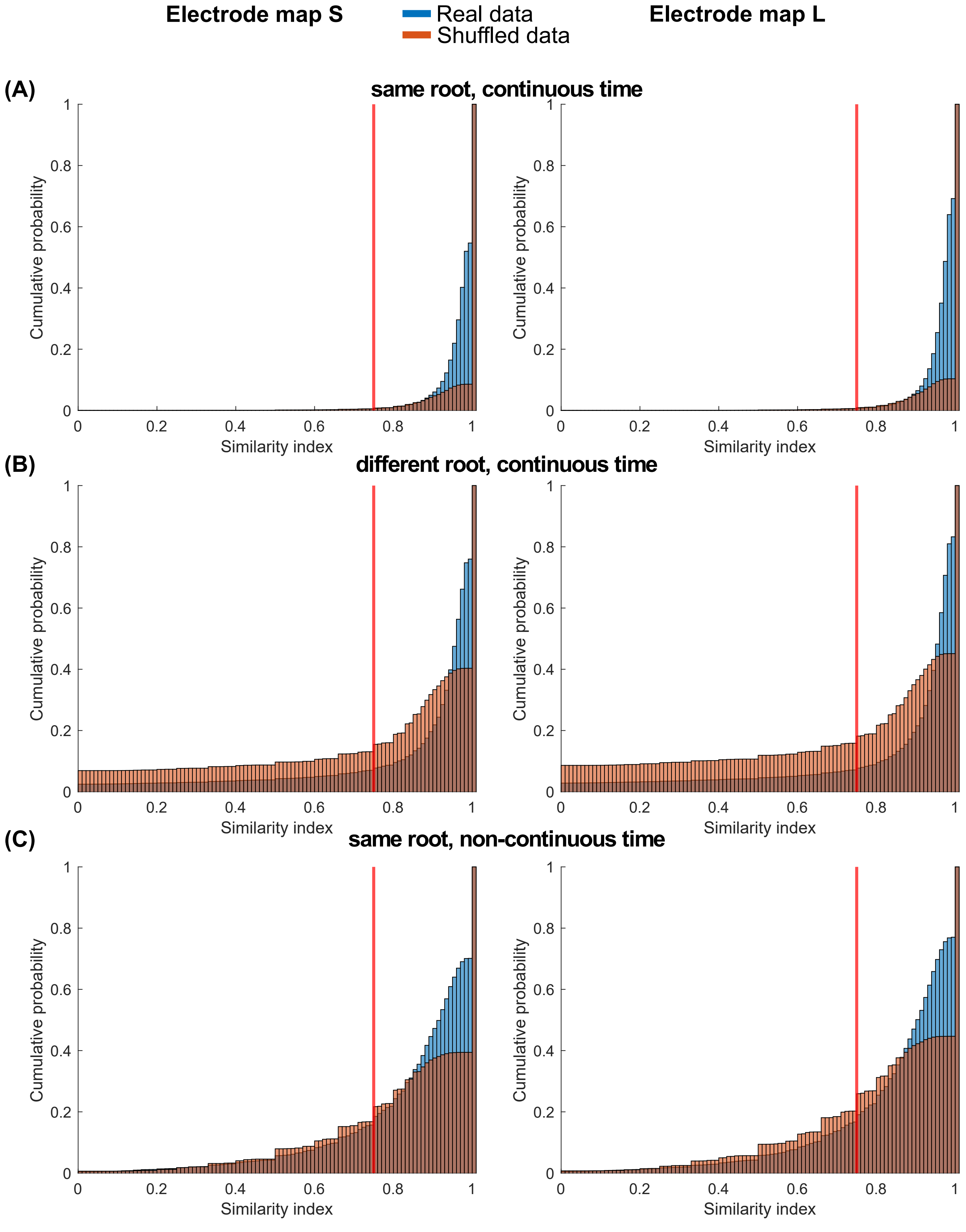
Electrode map S
Real data
Shuffled data
Electrode map L
(A)
same root, continuous time
(B)
different root, continuous time
(C)
same root, non-continuous time
Cumulative probability
Similarity index
0
0.2
0.4
0.6
0.8
1

Figure S14. Determine the similarity index threshold

Each panel shows the cumulative distribution of the similarity index (SI) for different conditions during concatenation. Vertical red lines indicate the SI threshold used (0.75).

(A) Same root and continuous time.

(B) Neighboring root and continuous time

(C) Same root and non-continuous time

(A)

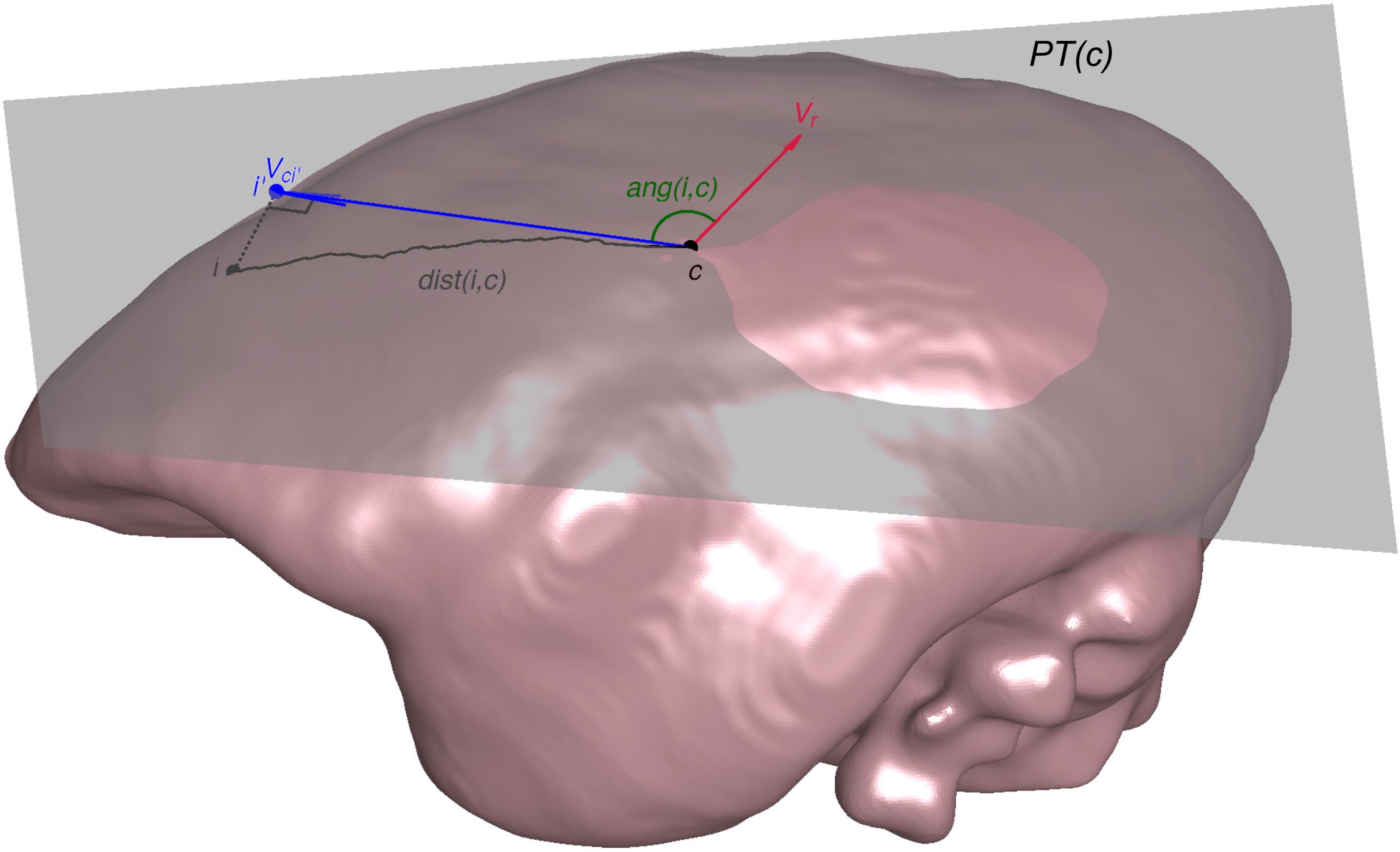

(B)

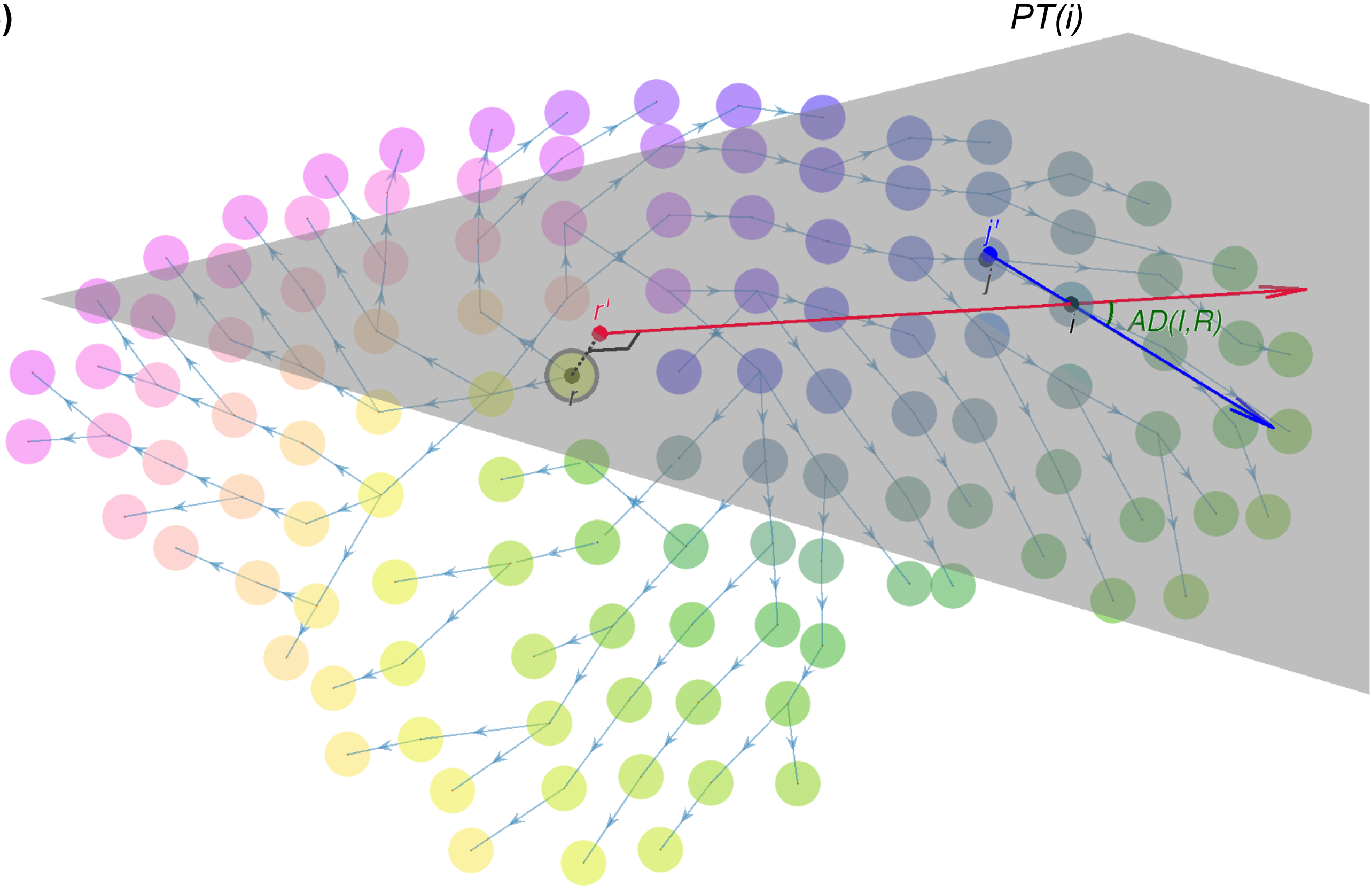

Figure S15. Illustration of definitions in spiral wave simulation

(A) The surrounding angle. *c*: spiral wave center; *i*: vertex of interest; *PT*(*c*): tangent plane at c; *i'*: projection of *i* on *PT*(*c*); *dist*(*i*, *c*): geodesic distance between *i* and *c*; *ang*(*i*, *c*): the surrounding angle at *i*.

(B) The angle of deviation. *r*: vertex closest to the identified root node *R*; *i*: vertex closest to the node of interest *I*; *j*: vertex closest to the preceding node *J*; *PT*(*i*): tangent plane at *i*; *r'*: projection of *r* on *PT*(*i*); *j'*: projection of *j* on *PT*(*i*); *AD*(*I*, *R*): the angle of deviation of *I* with regard to *R*.

4 Supplementary Videos

Video S1. Examples of the simulated waves (Marmoset S)

Phase maps of simulated data (4 sources, 50% noise) are shown on the electrode map of Marmoset S (Electrode map S). Black circles and colored arrows indicate the source and path of simulated (left) or identified (right) waves. Edges of the same wave have the same color.

Video S2. Examples of the simulated waves (Marmoset L)

Similar to Video S1, but for Electrode map L.

Video S3. Spiral wave simulation

Top row: phase of the simulated spiral waves plotted on the brain surface mesh of the two marmosets (electrode map S: left; electrode map L: right). Black dots indicate wave source. Bottom row: maximal directed spanning tree of the identified waves, given the phase at each electrode location. Black dots, black circles, and colored arrows indicate the true source, identified source, and path of the identified waves, respectively. Edges of the same wave have the same color. SPI: spiral index of the largest TW (see Supplementary method). As the spirality of the simulated wave changes, the electrode with the earliest phase also changes, and so is the identified source. Note that at around 200 ms in the bottom left panel, two electrodes are detected as source, with no neighboring electrode having preceding phase. This is reasonable given only data from the electrode positions, and is not an artifact caused by the algorithm. With higher electrode density, true wave sources are more likely to be identified.

Video S4. Example traveling waves identified by the graph-based algorithm (Marmoset S)

Example trial of Marmoset S shown in Figure 5 were processed by the graph-based algorithm, and the resulting MDSTs were plotted. Time is relative to saccade offset.

Video S5. Example traveling waves identified by the graph-based algorithm (Marmoset L)

Similar to Video S4, but for Marmoset L.

Video S6. Evolution of phase maps for different saccade directions around saccade offset (Marmoset S)

Data from Marmoset S are shown. Data criteria are the same as Figure 4 (sample size; 0°: 1355, 45°: 1350, 90°: 1238, 135°: 1248, 180°: 1281, 225°: 1357, 270°: 1024, 315°: 1377). Colors indicate the phase of the trial-averaged analytic signal and opacity indicates the ITPC, as shown in the central penal. Time is relative to saccade offset.

Video S7. Evolution of phase maps for different saccade directions around saccade offset (Marmoset L)

Similar to Video S6, but for Marmoset L (sample size; 0°: 1046, 45°: 1027, 90°: 1063, 135°: 1104, 180°: 1168, 225°: 1190, 270°: 1104, 315°: 1129).

Video S8. Evolution of edge asymmetry (Marmoset S)

Similar to Figure 8, but includes data of all eight directions during -50 to 100 ms surrounding saccade offset. Data from Marmoset S are shown. Sample size is the same as Video S6.

Video S9. Evolution of edge asymmetry (Marmoset L)

Similar to Video S8, but for Marmoset L. Sample size is the same as Video S7.
